# The Evolution and Ecology of Host Manipulation in Helminth Parasites: A Phylogenetic Meta‐Analysis

**DOI:** 10.1111/ele.70340

**Published:** 2026-02-18

**Authors:** Nina Hafer‐Hahmann

**Affiliations:** ^1^ Department of Biology University of Konstanz Konstanz Germany

**Keywords:** complex life cycle, evolution, extended phenotype, helminths, host behaviour, host manipulation, host–parasite interactions, meta‐analysis, trophic transmission

## Abstract

Parasites have repeatedly evolved the ability to modify their host's behaviour to enhance transmission. However, the conditions shaping such host manipulation remain unclear. I conducted a phylogenetic meta‐analysis of 207 studies comprising 1635 observations from 82 parasite and 80 host taxa to identify patterns in host manipulation and test ecological and evolutionary drivers of host manipulation in trophically transmitted helminths. Manipulation reliably increased host susceptibility to predation and effects depended strongly on parasite stage: mature parasites consistently enhanced predation susceptibility, whereas immature stages showed little or inconsistent impact. In mixed infections, mature parasites dominated, and manipulation was stronger in the presence of the correct predator. Phylogenetic constraints were minimal, in line with repeated independent origins. The ecological and evolutionary drivers and constraints tested here had only weak or inconsistent effects.

## Graphical abstract

Host manipulation by parasites is wide spread but the conditions shaping it remain unclear. In a phylogenetic meta‐analysis on trophically transmitted helminths, manipulation reliably increased host predation susceptibility to predation in mature parasites, while the effect of immature parasites remained unclear. No clear ecological and evolutionary drivers emerged.

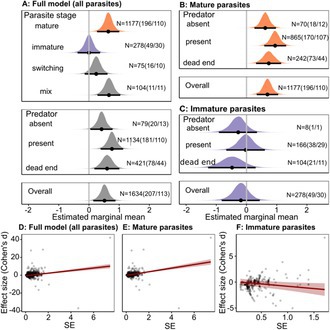

## 1 Introduction

Grasshoppers leaping into water, mice drawn to the scent of cat urine and ants masquerading as berries perched in plain view of birds—these behaviours seem maladaptive, even suicidal. And they are, for the host exhibiting them, but not for the parasites that cause them. These parasites alter their hosts' phenotypes to complete their own life cycles: nematomorphs compelling their insect hosts into water to reproduce (Thomas et al. 2002), the protozoan *Toxoplasma gondii* ensuring successful transmission to its final host, cats (da Silva and Langoni 2009) and a nematode that enhances transmission to its final host, birds (Poinar and Yanoviak 2008; Verble et al. 2012) respectively. While fatal for the host, these behaviours increase parasite fitness by ensuring that the parasite can complete its life cycle. This phenomenon—host manipulation—represents one of the prime examples of an extended phenotype—a trait encoded in one organism (in this case the parasite) but expressed elsewhere (the host) (Poulin 1994b). It has evolved repeatedly across diverse lineages and can take diverse forms such as increasing the host's predation susceptibility, protecting the host from predation, or inducing it to seek out a different habitat (Bhattarai et al. 2021; Hafer‐Hahmann 2024; Heil 2016; Maure et al. 2013; Poulin 1994a, 2010; Poulin and Maure 2015). Such host manipulation can have profound ecological, economic and epidemiological consequences, influencing food‐web stability, community dynamics and disease transmission (Hafer 2016; Hasik et al. 2023; Labaude, Rigaud, and Frank 2015; Lafferty and Kuris 2013; Sato et al. 2019).

Host manipulation is especially well studied in trophically transmitted parasites with complex life cycles, which must circumvent their intermediate host's innate predator avoidance to reach their next host. To do so, parasites change activity levels, social behaviours, anti‐predator behaviour, habitat preferences, appearance, physiology and impair various traits such as vision or motion, often changing multiple phenotypic traits simultaneously (Cezilly et al. 2013; Thomas et al. 2010). Manipulation can vary within species (Franceschi, Cornet, et al. 2010; Hafer 2018; Labaude, Cézilly, et al. 2015) and can converge across distantly related taxa with similar life cycles (Hafer and Milinski 2016; Ponton et al. 2006), highlighting the likely role of ecology in shaping host manipulation both over ecological and evolutionary time. At the same time, such complex life cycle parasites often seem to reduce their host's predation susceptibility prior to reaching maturity and infectivity to their subsequent host to prevent fatal premature predation (Fayard et al. 2020; Hammerschmidt et al. 2009; Maure et al. 2013).

A number of theoretical models has investigated the ecological conditions under which host manipulation should evolve (Oliver and Best 2024; Parker et al. 2009; Poulin 1994b; Seppälä and Jokela 2008; Vickery and Poulin 2009; de Vries and van Langevelde 2018). For instance, manipulation by mature parasites should be disfavored under high levels of baseline predation by suitable hosts, where passive transmission already ensures life‐cycle completion, but favored when such predation is rare and/or life span is short limiting the time for successful transmission (Oliver and Best 2024; Poulin 1994b; Seppälä and Jokela 2008; Vickery and Poulin 2009; de Vries and van Langevelde 2018). Host manipulation may carry a cost from increased dead‐end predation, that is, predation by predators unsuitable as hosts for the parasite that is hence fatal for the parasite. Hence, high rates of dead‐end predation should select against host manipulation that enhances predation or for host manipulation that specifically targets the correct subsequent host (Seppälä and Jokela 2008). In the only empirical investigation of these factors to date, Fayard et al. (2019) indeed observed a tendency for lower host manipulation by acantahcephalan parasites in a gammarid host in populations with higher fish (i.e., predator) bio mass. Conversely, host manipulation by immature parasites to suppress predation should be favored under high predation risk as any predation would be fatal to them (Parker et al. 2009; de Vries and van Langevelde 2018). Energetic costs of host manipulation are usually assumed by theoretical models and may generate trade‐offs with other fitness components (Poulin 1994b).

Previous meta‐analyses on how parasites affect host behaviour have synthesised patterns across systems (Fayard et al. 2020; Hasik et al. 2023; Lafferty and Shaw 2013; McElroy and de Buron 2014; Mrugała et al. 2023; Nakagawa et al. 2015; Poulin 1994a, 2000). These studies, for example, document widespread manipulation, variation across taxa and differences in host manipulation between parasite stages, for example, for Acanthocephalans (Fayard et al. 2020) or with regards to host performance (McElroy and de Buron 2014). Yet, evolutionary and ecological drivers and constraints of this variation remain unclear. Here, I present a phylogenetic meta‐analysis based on 1635 observations from 82 trophically transmitted helminth parasite and 80 host taxa, aiming to clarify not only where and when manipulation occurs but why it evolves.

## 2 Material and Methods

### 2.1 Literature Search

**FIGURE 1:**
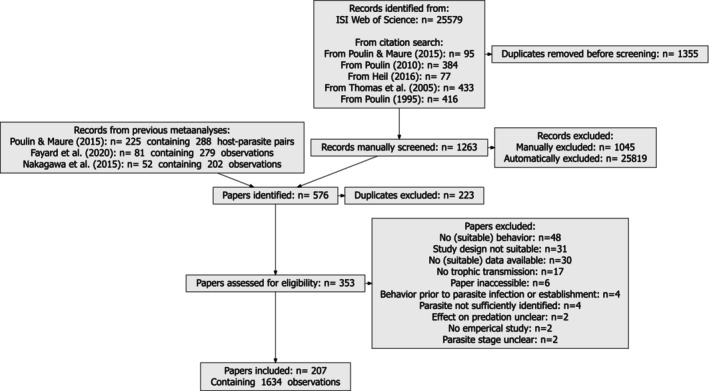
PRISMA diagram.

To keep the amount of data manageable, I focused on host manipulation by trophically transmitted helminths. These make up a large majority of trophically transmitted parasite species for which host manipulation has been studied. To identify appropriate studies I used a list of host manipulation studies compiled by Poulin and Maure (2015) for publications prior to 2015. For newer studies (2015–2023), I searched Web of Science using the same search terms (i.e., (parasite OR helminth* OR acanthoceph* OR nematode* OR digenea* OR trematod* OR cestod*) AND (behav* OR color* OR colour* OR phenotyp* OR respon* OR pigment*) AND (manipula* OR effect* OR change* OR alter* OR modif* OR transmi* OR predat*)). Additionally, I screened articles citing key reviews on host manipulation (Heil 2016; Poulin 1995, 2010; Poulin and Maure 2015; Thomas et al. 2005). All references were imported into Rayyan (Ouzzani et al. 2016) and inclusion/exclusion decisions were based on titles and abstracts with iterative scoring until only likely exclusions remained. I retained only empirical studies that: (1) examined behaviour or appearance in hosts from which parasites are trophically transmitted, (2) provided sufficient data for effect size calculation, (3) included uninfected controls, (4) identified parasites at least to genus level, (5) measured behaviour after the infection had established and allowed the identification of parasite stage and (6) allowed determination of likely effects on predation susceptibility. Studies focusing on reproduction, mate choice, or purely physiological responses (e.g., growth) were excluded. Because distinguishing true host manipulation from incidental side effects is contentious (Hafer‐Hahmann 2024; Milinski 1990; Nadler et al. 2023; Poulin 2010; Thomas et al. 2005), I included any behavioural change plausibly increasing parasite transmission via altered predation susceptibility. Data from two recent meta‐analyses (Fayard et al. 2020; Nakagawa et al. 2015) were integrated. This resulted in 1635 observations from 82 parasite and 80 host taxa from 207 papers (see Figure 1 for PRISMA workflow and Table S1 for a list of included studies and host–parasite pairs).

### 2.2 Data Extraction and Preparation

#### 2.2.1 Extraction of Response Variables and Effect Size Calculation

Effect sizes were expressed as Cohen's *d* comparing infected versus uninfected hosts. Cohen's *d* is a standardised measure of effect size that quantifies the difference between two group means in units of their pooled standard deviation, allowing comparisons across studies and measurement scales. Cohen's *d* was extracted directly from studies or previous meta‐analyses when available; otherwise, I calculated *d* from means and errors, raw data, or digitised plots (using *digitise* (Poisot 2011) or *metaDigitise* (Pick et al. 2019) in R) using the R package esc (Lüdecke 2018). If only medians and IQR were available, mean was approximated as median and SD as *IQR/1.35*. When necessary, test statistics (*F*, *t*, *χ* ^2^, *z*, *r*) were transformed to *d* using the effectsize package (Ben‐Shachar et al. 2020) or, when only *U* was available, an online resource (Lenhard and Lenhard 2022). The standard error of *d* was calculated as: ${\mathrm{SE}}_{d}=\sqrt{\frac{\left(n_{U}+n_{I}\right)}{n_{U}n_{i}}+\frac{d^{2}}{2\left(n_{U}+n_{I}\right)}}$ (Borenstein et al. 2009), where *U* and *I* are uninfected and infected hosts respectively.

Behavioural responses were classified into six categories: activity, microhabitat choice, predation‐related responses (excluding anything that could be attributed to any of the other categories), social and aggressive behaviour and impairment/appearance. For each observation, I recorded parasite stage (mature, immature, switching or mixed), predator context (correct subsequent host or generic predator stimulus, dead‐end predator, or no predator) and infection procedure (natural vs. experimental), host and parasite identity, publication year and publication identity.

The direction of *d* was standardised based on predation susceptibility and positive when infected hosts were assumed to be more susceptible to predation and negative when they appeared less susceptible.

#### 2.2.2 Additional Host and Parasite Traits

Potential factors that might drive or constrain host manipulation were obtained for each species included in the data set. For hosts, I obtained different potential proxys for predation susceptibility (host size, trophic level and the proportion of trophic interactions as predator) and longevity. For parasites, I obtained adult body size (proxy for fitness) and infection site. Additionally, I obtained habitat, next host type and trophic link (i.e., a combination of current and next host). Numeric traits were transformed as appropriate prior to analysis. See Table S2 for details on each trait. This data was obtained from various sources, most importantly fish base and sea live base (Boettiger et al. 2012; Froese and Pauly 2025; Palomares and Pauly 2025), anage (Tacutu et al. 2012), the Global Biotic Interactions data base (Poelen et al. 2014) and data previously compiled by Benesh (2016) and Benesh et al. (2017).

Host and parasite phylogenies were constructed using the Open Tree of Life (Redelings et al. 2019) and completed using rotl (Michonneau et al. 2016), phytools (Revell 2024) and ape (Paradis and Schliep 2019) in R. Missing taxa were substituted by close relatives. Two species were manually relocated based on taxonomy. See Figure S1 for phylogenetic trees. Since alternative arthropod phylogenies to the one used by Open tree of life have been proposed (e.g., Chang and Lai 2018; GIGA Community of Scientists 2014; Thomas et al. 2020) and arthropods made up the majority of hosts in my study, I reran some models with an alternative host phylogeny (see Methods S1 and Results S1).

### 2.3 Statistical Analysis

All analyses were performed using R 4.1.3 (R Core Team 2023). Plots were created using ggplot2 (Wickham 2016).

I fitted Bayesian hierarchical meta‐analytic models using the brms package (Bürkner 2017), with cmdstanr as backend (Gabry et al. 2025). Models incorporated multiple sources of non‐independence, that is, host and parasite phylogeny, host–parasite interaction and study identity. Models assumed a Student's *t*‐test distribution for likelihood to account for outliers and heavy‐tailed data. Fixed effects (regression coefficients) had weakly informative Student's *t*‐test priors (3 degrees of freedom, centered at 0, scale = 1). The intercept prior was Student's *t*‐test, scaled to the mean effect size. Random effect standard deviations had Student's *t*‐test (3, 0, 0.5) priors. The degrees of freedom parameter (ν) of the Student's *t*‐test was assigned a Gamma (2, 0.1) prior. When models lacked fixed effects, priors on regression coefficients were omitted. I ran all models with 4 chains, 10,000 iterations (4000 warm‐up), an adaptation target delta of 0.999, and a maximum tree depth of 15 to ensure stable convergence and effective sampling. I confirmed the validity of each model through R‐hat, ESS (Effective sample size) and pareto *k* values (see Tables S3–S6) and by visually inspecting diagnostic plots (see Figure S2).

The meta‐analytic response variable was Cohen's *d* with known standard errors, allowing propagation of sampling uncertainty. Fixed effects included standard error (to account for publication bias; Bayesian analog to Egger's regression; Nakagawa et al. 2022), parasite stage (immature, mature, switching, or mixed), publication year, experimental procedure (natural vs. experimental infection), behaviour type and predator type, and the interaction between parasite stage and predator type. I fitted one model on the full data set (including all parasite stages) and separate models for mature and immature parasites (without parasite stage). Because the ultimate aim of host manipulation considered here is to increase (mature parasites) or decrease (immature parasites) predation, I also analysed models that included only data from direct measures of predation susceptibility, but were otherwise the same (without type of behaviour). To test whether factors suspected to drive or constrain the evolution of host manipulation affected the extent of host manipulation, I added these factors individually to the previously identified best models on mature and on immature parasites (see above and Table S2 for a list of these factors). Rather than modulating the strength of host manipulation, some of these factors may modify which type of behaviour is altered. Hence, I additionally ran models that included the type of behaviour and its interaction with any of these factors that was categorical.

Model selection followed a stepwise approach using leave‐one‐out cross‐validation (LOO) to compare predictive performance of a model with each fixed factor added individually to the previous best model without this factor via expected log predictive density (ELPD), with more complex models favored only when the difference in ELPD (ΔELPD, i.e., difference in expected log predictive density—a measure for how well a model predicts new data) exceeded twice the standard error using the loo package (Vehtari et al. 2017). Post hoc comparisons were conducted using emmeans to estimate marginal means and pairwise contrasts (Lenth 2019).

To assess robustness, I repeated analyses excluding extreme effect sizes (*d* < −5 or *d* > 5) and outliers. Outliers were identified from posterior residuals and summarised per observation (mean and 95% credible interval) as observations whose intervals excluded zero.

## 3 Results

### 3.1 Magnitude and Variability of Host Manipulation

#### 3.1.1 Difference in Host Manipulation Between Parasite Stages

**FIGURE 2:**
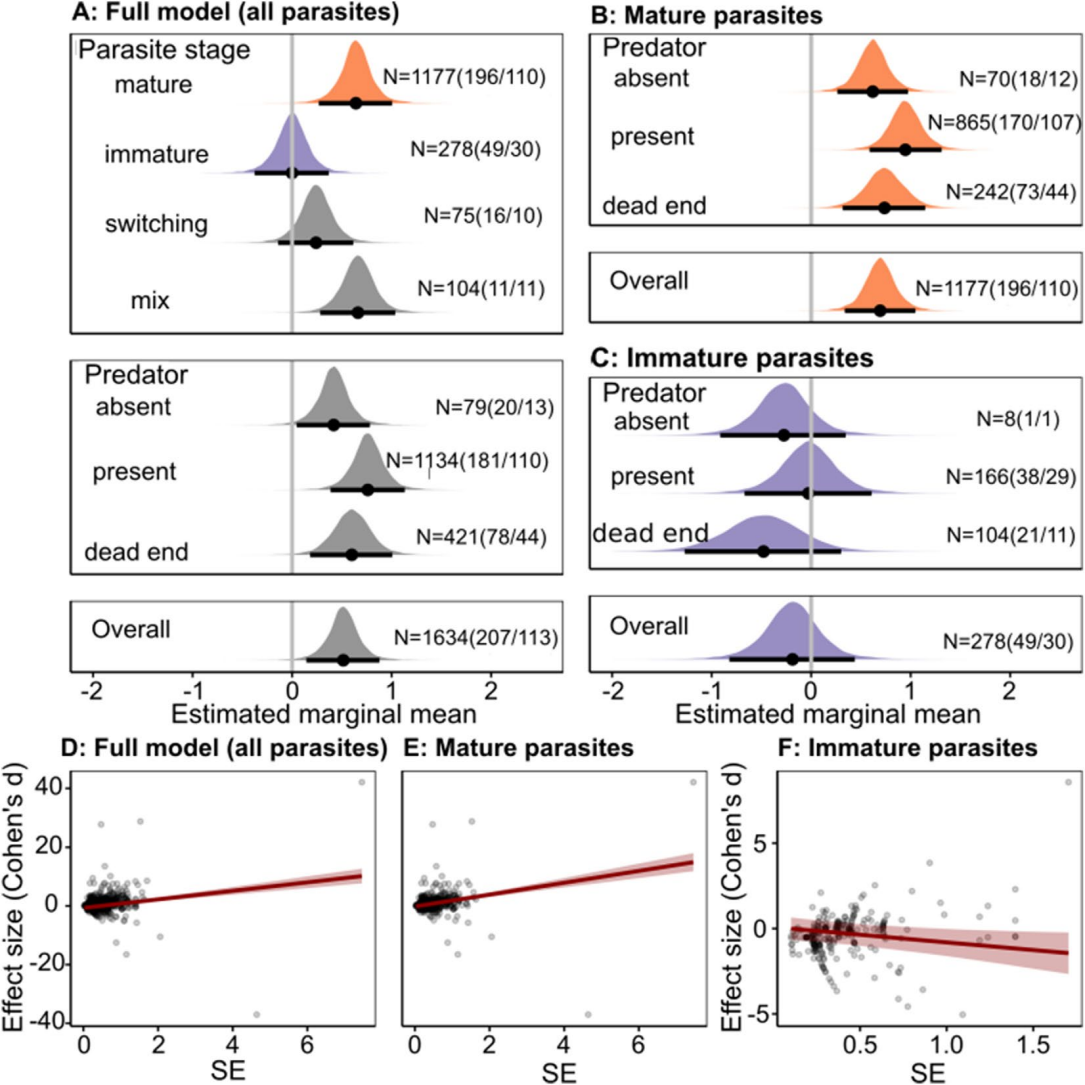
Estimated marginal means and marginal posterior distributions for the effect of parasites on host behaviour (A, B) and the relationship between effect size (Cohen's *d*) and its standard error (SE; D–F). Higher (positive) effect sizes indicate an increase in predation susceptibility while lower (negative) effect sizes indicate a decrease in predation susceptibility (or increased predation suppression). *N* indicates number of observations (number of studies/number of unique host–parasite pairs). Error bars indicate 95% highest posterior density (HPD) interval. (A, D) Full model (all parasites); (B, E) Mature parasites; (C, F) Immature parasites.

Manipulating parasites substantially altered host behaviour, making hosts likely more susceptible to predation (positive estimate; 95% HPD (High posterior density—i.e., the region containing 95% of the posterior distribution) range excluded 0; Table 1A, Figure 2A). This effect was strongly stage‐dependent (Table 2A). Mature parasites caused the most pronounced increase in predation susceptibility (Table 1A,B, Figure 2A,B). In contrast, immature parasites had no clear impact (HPD range overlapped 0; Table 1A,C, Figure 2A,C). As expected, the behavioural effects of mature and immature stages differed clearly (Table 1A). Hosts with transitioning parasites showed intermediate changes, while mixed infections—expected to involve conflict between stages—resembled mature infections and differed clearly from immature ones (Table 1A, Figure 2A). Patterns remained consistent when outliers were excluded (Table 2, Table S7, Figure S3).

**TABLE 1:** Estimated marginal means and contrasts for the best model.

A: Full model (all parasites)				
Estimates for fixed factors				
Factor	Level	Emmean/trend (HPD range)	*N*	Unique taxa
**Overall**	**Overall**	**0.464 (0.074 to 0.817)**	**1634/207**	**113 (80/82)**
**SE**	**Overall**	**1.454 (1.106 to 1.802)**	**1634/207**	**113 (80/82)**
Parasite stage	Immature	0.078 (−0.316 to 0.437)	278/49	30 (25/27)
**Parasite stage**	**Mature**	**0.72 (0.349 to 1.09)**	**1177/196**	**110 (79/79)**
**Parasite stage**	**Mix**	**0.74 (0.362 to 1.123)**	**104/11**	**11 (10/10)**
Parasite stage	Switching	0.32 (−0.065 to 0.7)	75/16	10 (8/9)
Predator	Absent	0.289 (−0.075 to 0.664)	1134/181	110 (77/81)
**Predator**	**Dead end**	**0.47 (0.042 to 0.868)**	**79/20**	**13 (10/10)**
**Predator**	**Present**	**0.631 (0.247 to 0.992)**	**421/78**	**44 (36/31)**

Contrasts		
Factor	Contrast	Estimate (HPD range)
**Parasite stage**	**Immature—mature**	**−0.641 (−0.74 to −0.544)**
**Parasite stage**	**Immature—mix**	**−0.664 (−0.802 to −0.525)**
**Parasite stage**	**Immature—switching**	**−0.241 (−0.358 to −0.125)**
Parasite stage	Mature—mix	−0.022 (−0.132 to 0.101)
**Parasite stage**	**Mature—switching**	**0.4 (0.287 to 0.506)**
**Parasite stage**	**Mix—switching**	**0.421 (0.276 to 0.554)**
Predator	Absent—dead end	−0.181 (−0.386 to 0.02)
**Predator**	**Absent—present**	**−0.344 (−0.423 to −0.263)**
Predator	Dead end—present	−0.163 (−0.374 to 0.054)

B: Mature parasites				
Estimates for fixed factors				
Factor	Level	Emmean/trend (HPD range)	*N*	Unique taxa
**Overall**	**Overall**	**0.767 (0.414 to 1.137)**	**1177/196**	**110 (79/79)**
**SE**	**Overall**	**2.024 (1.582 to 2.441)**	**1177/196**	**110 (79/79)**
**Predator**	**Absent**	**0.619 (0.246 to 0.954)**	**865/170**	**107 (76/78)**
**Predator**	**Dead end**	**0.737 (0.323 to 1.151)**	**70/18**	**12 (9/9)**
**Predator**	**Present**	**0.947 (0.594 to 1.316)**	**242/73**	**44 (36/31)**

Contrasts		
Factor	Contrast	Estimate (HPD range)
Predator	Absent—dead end	−0.118 (−0.348 to 0.117)
**Predator**	**Absent—present**	**−0.329 (−0.435 to −0.229)**
Predator	Dead end—present	−0.211 (−0.452 to 0.034)

C: Immature parasites				
Estimates for fixed factors				
Factor	Level	Emmean/trend (HPD range)	*N*	Unique taxa
Overall	Overall	−0.261 (−0.9 to 0.399)	278/49	30 (25/27)
**SE**	**Overall**	**−0.893 (−1.667 to −0.106)**	**278/49**	**30 (25/27)**
Predator	Absent	−0.274 (−0.923 to 0.336)	166/38	29 (24/27)
Predator	Dead end	−0.479 (−1.3 to 0.276)	8/1	1 (1/1)
Predator	Present	−0.027 (−0.675 to 0.598)	104/21	11 (9/9)

Contrasts		
Factor	Contrast	Estimate (HPD range)
Predator	Absent—dead end	0.203 (−0.284 to 0.681)
**Predator**	**Absent—present**	**−0.249 (−0.386 to −0.112)**
Predator	Dead end—present	−0.453 (−0.939 to 0.056)

*Note:* *N* indicates the number of observations/number of independent studies. Unique taxa indicates the number of unique host–parasite‐pairs (unique host taxa/unique parasite taxa). As far as possible, these estimates are based on species. However, in a limited number of cases, in which species level identification of parasites was not available, it is based on genus. Bold rows indicate estimates or contrasts whose HPD (High posterior density) range does not overlap with 0, that is, that estimates for these levels differ from 0 and from each other respectively.

**TABLE 2:** Results of model comparisons using leave‐one‐out cross‐validation (LOO).

Model	Basic models				Outliers excluded			
	ELPD loo	p loo	ΔELPD	Δse	ELPD loo	p loo	ΔELPD	Δse
**A: All parasites**								
*SE + parasite stage + predator + predator: parasite stage*	*−2011*	*329.8*	*10.21*	*6.95*	*−1562*	*340.9*	*11.56*	*7.57*
**SE + parasite stage + predator**	**−2021**	**322.6**	**39.16**	**12.02**	**−1574**	**337.5**	**49.05**	**13.1**
*SE + parasite stage + behaviour*	*−2043*	*290.6*	*16.86*	*10.23*	*−1611*	*321.6*	*11.96*	*9.56*
*SE + parasite stage + infection*	*−2055*	*305.2*	*5.28*	*3.3*	*−1617*	*322.3*	*5.82*	*3.26*
SE + parasite stage + year	−2058	309.6	2.34	3.48	*−1617*	*324.3*	*5.35*	*3.76*
**SE + parasite stage**	**−2060**	**305.6**	**72.52**	**20.14**	**−1623**	**328.4**	**76.34**	**20.44**
*SE*	*−2132*	*334.4*	*21.2*	*11.18*	*−1699*	*332.1*	*19.03*	*10.32*
1	−2154	347.1	0	0	−1718	337.6	0	0
**B: Mature parasites**								
**SE + predator**	**−1475**	**287.6**	**23.6**	**8.68**	**−1132**	**287.5**	**28.59**	**9.98**
*SE + behaviour*	*−1478*	*276.6*	*20.03*	*11.11*	−1144	283.7	17.17	23.7
*SE + infection*	*−1493*	*282.3*	*5.7*	*3.26*	*−1156*	*282.8*	*4.55*	*3.27*
SE + year	−1498	287.7	0.61	2.74	−1159	283.3	2.15	2.97
**SE**	**−1498**	**286.9**	**34.67**	**12.42**	**−1161**	**287.1**	**34.16**	**11.94**
1	−1533	294.4	0	0	−1195	290.7	0	0
**C: Immature parasites**								
**SE + predator**	**−278**	**74.3**	**10.64**	**3.96**	**−229**	**69.3**	**8.81**	**3.69**
SE + behaviour	−288	74.3	0.46	3.81	−236	68.4	1.64	4.19
SE + infection	−287	73.1	1.15	1.54	*−236*	*66.7*	*1.77*	*1.52*
*SE + year*	*−285*	*73*	*2.96*	*2.12*	*−235*	*66.7*	*2.5*	*1.97*
*SE*	*−288*	*75.2*	*3.59*	*2.81*	*−238*	*68.8*	*4.01*	*2.54*
1	−292	72	0	0	−242	67.6	0	0

*Note:* Results were obtained through the Bayesian modelling framework (BRMS). Factors that clearly improved the model are indicated in bold (i.e., ΔELPD larger than two times SE), those for which an improvement was observed, but was unclear due to ΔELPD being between one and two times SE are highlighted in italics. Comparisons are always to the preceding model that was clearly better (i.e., highlighted in bold). See Table S3 for details on model diagnostics. All models included the following random effects: Paper ID, Host and parasite phylogeny and the interaction between host and parasite (see Table 3B for information on heteorgeneity and Table 3A for the proportion of variance explained by each random effect).

Abbreviations: ELPD loo, expected log predictive density estimated via LOO cross‐validation; p loo, effective number of parameters, indicating model complexity; ΔELPD, difference in ELPD loo between models; Δse, standard error of ΔELPD, indicating uncertainty in model differences.

### 3.2 Ecological Context: Predator Presence Is Associated With Stronger Host Manipulation

Host manipulation varied with the predation risk experienced by the host (ΔELPD > 2*SE; Table 2). It was stronger when the correct predator was present than in the absence of any predator (Table 1, Figure 2). Interestingly, the same pattern appeared for immature parasites—even though any predation is fatal to them—although their manipulation never clearly differed from zero (Table 1C, Figure 2C). It was unclear whether including the interaction between parasite stage and predator type in the full model improved model fit (ΔELPD between one and two SE indicating that both models fit similarly well; Table 2A). In this model predator effects were only clear (i.e., 95% HPD interval not overlapping zero) for hosts with mature parasites or a mix of parasites and host manipulation by mature parasites was most pronounced in the presence of the correct predator (Table S8, Figure S4A).

### 3.3 Limited Differences Between Behaviours and Experimental Settings

Surprisingly, type of behaviour did not clearly affect the strength of host manipulation. Any improvement by including behaviour in the model was unclear (ΔELPD within one to two SE; Table 2). If behaviour was nevertheless retained in the model, effects were strongest for impairment and weakest for activity for the full model and the one on mature parasites (Table S9A,B, Figure S5A,B). Immature parasites showed a similar pattern, with the strongest positive value for impairment and the lowest (negative) value—indicating predation suppression—for social behaviour. Indeed, social behaviour was the only category to show any clear difference from zero for immature parasites, but this should be treated cautiously since data came from only three studies (Table S9C, Figure S5C). When only direct measures of predation susceptibility were analysed (see Table S6), the strength of host manipulation was in the same range as in the models that included all behaviours (*N* = number of observations/papers): full model: 0.413 (−0.295–1.118), *N* = 74/34; model on mature parasites: 1.044 (0.306–1.815), *N* = 65/32; model on immature parasites: −0.131 (−0.821–0.586), *N* = 8/6.

Infection method (natural vs. experimental) did not have a clear effect (ΔELPD between 1 and 2 times SE for the full model and mature parasites; Table 2). Nevertheless, in models that included infection method, manipulation was weaker in experimentally infected hosts (full model: 0.181 (−0.083–0.443); *N* = 1023/85 vs. 0.522 (0.256–0.784); *N* = 611/127; contrast: −0.342 (−0.524 to −0.166); mature parasites: 0.467 (0.201–0.744); *N* = 609/75 vs. 0.846 (0.581–1.112); *N* = 568/126; contrast: −0.377 (−0.572 to −0.196), Figure S4B,C).

Patterns remained similar when outliers were removed (Table 2).

### 3.4 Strong Evidence of Publication Bias

I included standard error (SE) in the models to test for publication bias. If publication bias is present studies that report the expected effect are more likely to be published and this effect is more pronounced for studies with small sample sizes and larger errors. Hence, if publication bias occurs there should be a correlation between effect size and SE. In the model on mature parasites including SE as a fixed effect caused a marked improvement in model fit (Table 2B) indicative of publication bias. The relationship between SE and effect size was very strongly positive (Table 1B, Figure 2E). In the other models, it was unclear whether including SE improved model fit (i.e., ΔELPD between and one and two SE (Table 2A,C)). Nevertheless, in the model on all parasites, the relationship between effect size and SE was again very strongly positive (Table 1A, Figure 2D). In contrast, in the model on immature parasites, this relationship was strongly negative (Table 1C, Figure 2F), again consistent with the expectation that studies showing the predicted effect—in this case, predation suppression by immature parasites—are more likely to be published. In either case the HPD interval for SE did not overlap zero. In addition, the presence of publication bias is also supported by funnel plots (see Figure S6). SE was therefore included as a fixed effect in all models to account for this publication bias. Similar publication bias was identified when outliers were removed (Table 2, Table S7, Figure S7).

There was no evidence of an effect of year on host manipulation in the full model or the model on mature parasites (Table 2A,B). In the model on immature parasites any effect of year was unclear (ΔELPD between one and two times SE; Table 2C); year had a slight negative effect whose HPD range did not overlap zero (estimated slope (HPD range): −0.028 (−0.049 to −0.007); *N* = 278 observations from 49 papers, Figure S5D), indicating a reduction of the reported effect sizes for predation enhancement or an increase in studies reporting predation suppression over time. This would be consistent with an increase in the number of studies that find the expected direction of host manipulation.

### 3.5 Minimal Evidence of Phylogenetic Constraints

Parasite phylogeny, included as a random effect, accounted for a very small proportion of variance (full model: 0.3%; mature parasites: 0.6%; immature parasites: 3.1%). Host phylogeny explained slightly more variance (full model: 2.8%; mature parasites: 2.1%; immature parasites: 5.3%). The interaction between host and parasite taxa contributed similarly little in the full model and for mature parasites, but much more strongly for immature parasites (19.3%). This result was similar when I reran models with an alternative host phylogeny (see Methods S1 and Results S1). Patterns in heterogeneity were consistent with these findings and similar for models without outliers (Table 3).

**TABLE 3:** Heterogeneity (A) and proportion of variance explained by random effects (B) for different models.

A: Variation explained by each random effect					
Model	Interaction (host–parasite pair)	Paper	Parasite phylogeny	Host phylogeny	Residual
All parasites	1.3%	11.3%	0.3%	2.8%	84.2%
All parasites, outliers removed	1.7%	17%	0.4%	3.9%	76.9%
Mature parasites	1.5%	13.9%	0.6%	2.1%	81.9%
Mature parasites, outliers removed	1.8%	19.8%	0.8%	2.7%	74.9%
Immature parasites	19.3%	10.7%	3.1%	5.3%	61.6%
Immature parasites, outliers removed	19%	13.6%	3.3%	6.1%	58%

B: Heterogeneity (*I* ^2^)					
Model	Host phylogeny	Interaction (host–parasite‐pair)	Paper	Parasite phylogeny	Overall
All parasites	11.94% (0.07%–41.07%)	5.34% (0.16%–14.5%)	39.73% (24.51%–53.1%)	1.77% (0%–10.28%)	58.78% (47.86%–72.54%)
All parasites, outliers removed	12.92% (0.07%–44.29%)	5.52% (0.3%–14.76%)	47.19% (28.51%–60.9%)	1.86% (0%–10.89%)	67.49% (58.21%–79%)
Mature parasites	8.63% (0.03%–33.03%)	5.4% (0.5%–14.17%)	44.41% (30.27%–56.06%)	2.73% (0%–13.95%)	61.16% (51.99%–71.66%)
Mature parasites, outliers removed	9.08% (0.03%–34.35%)	5.22% (0.46%–13.78%)	51.9% (35.52%–63.79%)	2.99% (0%–14.75%)	69.19% (61.67%–78.03%)
Immature parasites	12.73% (0.01%–64.29%)	37.37% (4.42%–65.04%)	20.46% (5.54%–43.74%)	7.92% (0.01%–37.77%)	78.49% (64.09%–90.35%)
Immature parasites, outliers removed	13.6% (0.01%–66.2%)	34.75% (2.29%–63.03%)	24.01% (7.26%–48.47%)	7.71% (0.01%–37.44%)	80.06% (67.81%–91.07%)

### 3.6 Substantial Heterogeneity and Study‐Level Variation

Overall, heterogeneity was moderate to high across models (between approximately 50% and 80% depending on the model). A substantial share of heterogeneity was attributed to variation among studies (between 20% and 45%; Table 3B). Consistently, the study‐level random effect accounted for a large proportion of variance (between 10% and 14%; Table 3A).

Results were similar after excluding outliers (Table 3).

### 3.7 Ecological and Evolutionary Drivers and Constraints of Host Manipulation

None of the ecological factors and potential constraints investigated showed any clear impact on host manipulation (Table 4). However, for mature parasites some factors slightly improved model fit, but these improvements were always between one and two times SE rendering them inconclusive and these effects vanished when outliers were excluded (Table 4A). Post hoc comparisons, nevertheless, revealed that parasites using birds (both aquatic and terrestrial, albeit aquatic birds were over represented in the data set) as subsequent hosts exhibited substantially stronger manipulation than those using terrestrial vertebrates (primarily mammals) (difference HPD range excluded zero; Table S10A, Figure S8A). Parasites targeting aquatic vertebrates (mainly fish) were intermediate. Other ecological and life‐history traits showed no clear effects (Table S10B–D, Figure S8B–D).

**TABLE 4:** Results of model comparisons using leave‐one‐out cross‐validation (LOO) to investigate factors that may drive or constrain the evolution of host manipulation (see Table S2 for more details on these factors).

Model	Basic models				Outliers excluded			
	ELPD loo	p loo	ΔELPD	Δse	ELPD loo	p loo	ΔELPD	Δse
**A: Mature parasites**								
+trophic link	−1474	286.5	0.26	2.23	−1132	287.7	0.49	21.89
*+next host type*	*−1473*	*284.6*	*1.87*	*1.78*	−1129	285.3	2.72	22.00
*+adult parasite size*	*−1474*	*287.5*	*0.78*	*0.73*	−1133	289.6	−0.61	0.66
*+infection site*	*−1473*	*288*	*1.34*	*1.29*	−1131	287.6	1.32	21.89
+parasite type	−1475	290.5	−0.13	1.02	−1132	288.7	0.63	0.87
+trophic interactions as predator (arcsine transformed)	−1475	287.9	0.13	0.33	−1132	287.9	0.65	21.91
+trophic level	−1474	287.8	0.3	0.9	−1131	286.8	1.68	21.89
+host life span (log transformed)	−1475	289.5	−0.11	0.69	−1132	288.9	−0.1	0.62
+host size (log transformed)	−1475	288.7	−0.07	0.4	−1132	288.1	0.16	0.37
+host type	−1473	290	1.44	1.6	−1130	288.1	2.15	21.96
*+habitat*	*−1473*	*287.5*	*1.2*	*0.87*	−1129	286.1	2.8	21.94
**SE + predator**	**−1475**	**287.6**			**−1132**	**287.5**		
**B: Immature parasites**								
+trophic link	−278	74.6	−0.16	1.74	−229	69.1	−0.17	1.64
+next host type	−279	76.8	−1.48	0.51	−230	71.1	−1.29	0.51
+adult parasite size	−279	76.4	−1.74	0.84	−230	70.5	−0.91	0.77
+infection site	−278	75.6	−0.27	0.51	−229	69.6	0.15	0.41
+parasite type	−278	76.2	−0.6	0.67	−229	70.2	−0.28	0.46
+trophic interactions as predator (arcsine transformed)	−279	75.7	−0.97	0.3	−229	69.9	−0.55	0.35
+trophic level	−280	77.2	−2.38	1.22	−230	71.2	−1.52	0.93
+host life span (log transformed)	−278	75.1	−0.28	0.35	−229	69.5	−0.08	0.37
+host size (log transformed)	−279	75.7	−0.95	0.35	−229	70.3	−0.74	0.42
+host type	−277	73.2	0.39	1.4	−229	68.7	−0.32	1.31
+habitat	−279	76.4	−1.47	0.46	−229	70.3	−0.74	0.39
**SE + predator**	**−278**	**74.3**			**−229**	**69.3**		

*Note:* Results were obtained through the Bayesian modelling framework (BRMS). Factors that clearly improved the model are indicated in bold (i.e., ΔELPD larger than two times SE), those for which an improvement was observed, but was unclear due to ΔELPD being between one and two times SE are highlighted in italics. Comparisons are always to the preceding model that was clearly better (i.e., highlighted in bold). See Table S4 for details on model diagnostics. All models included the following random effects: Paper ID, Host and parasite phylogeny and the interaction between host and parasite.

Abbreviations: ELPD loo, expected log predictive density estimated via LOO cross‐validation; p loo, effective number of parameters, indicating model complexity; ΔELPD, difference in ELPD loo between models; Δse, standard error of ΔELPD, indicating uncertainty in model differences.

Similarly, evidence that ecological factors shape what behaviour is targeted by manipulation rather than its overall magnitude was weak at best (Table S11A). For mature parasites, it was unclear (ΔELPD between and one and two times SE) whether interactions between next‐host type and behaviour improved model fit. In line with the potential effect of next host as a main effect, manipulation was generally strongest in parasites transmitted to birds, intermediate for those targeting aquatic vertebrates and weakest for those exploiting terrestrial vertebrates across most behaviours. The strength of these differences varied between behaviours and differences were especially pronounced for impairment and position (i.e., HPD range of differences between next host types not overlapping zero, Table S12, Figure S9). However, when outliers were removed, any effect of an interaction between behaviour and next host type completely disappeared (Table S11A). For immature parasites any potential behaviour–factor interactions (i.e., ΔELPD>SE; Table S11B), were driven by social behaviour and based on eight observations from three studies, rendering them inconclusive.

## 4 Discussion

The extend and possibly direction, of host manipulation is strongly associated with parasite developmental stage. Mature parasites consistently increase behaviours likely to enhance host predation susceptibility with medium‐to‐large effect sizes, similarly to patterns previously observed in Acanthocephalan parasites (Fayard et al. 2020) or a much smaller data set (Poulin 1994a). In contrast, immature parasites exert little influence overall, despite clear evidence in some systems (Dianne et al. 2011; Hafer and Milinski 2016; Hammerschmidt et al. 2009), a previous meta‐analysis on Acanthocephalans suggesting effects of similar magnitude, but opposite direction as in mature parasites (Fayard et al. 2020) and theoretical predictions that predation suppression by immature parasites should evolve more readily than predation enhancement by mature parasites (Parker et al. 2009). This discrepancy likely reflects lineage‐specific or even host–parasite pair‐specific strategies, as indicated by the high variation explained by host–parasite interaction for immature parasites in the present analysis. Functional tests of host manipulation (i.e., whether infection truly increases predation susceptibility) remain scarce compared to descriptive studies (Poulin and Maure 2015). While this gap persists, my results indicate that behavioural measures are in a similar range as direct measurements of predation susceptibility and may hence present reliable proxies in many cases.

When immature and mature parasites co‐inhabit the same host, their interests are at conflict: transmission benefits one, but is fatal for the other. This analysis provides strong evidence that mature parasites typically dominate this conflict at least with regards to host manipulation, a result consistent with experimental work (Dianne et al. 2010; Hafer and Milinski 2015c, 2015a, 2016). While this outcome might seem predictable given the weak overall effect of immature parasites, previous studies demonstrate that mature parasites can prevail even when immature stages are normally stronger manipulators (Hafer and Milinski 2015c, 2016). Co‐infections remain understudied, but theoretical models suggest that they can reshape ecological dynamics and community structure, especially when host manipulation is involved (Nguyen and Gokhale 2025). The current results imply that in co‐infections, host behaviour largely reflects the manipulation of mature parasites.

Phylogenetic constraints on host manipulation appear weak, as suggested by the limited phylogenetic signal. This is in line with prior conclusions that host manipulation has evolved multiple times independently across parasite lineages (e.g., Poulin 1994a), varies among populations of the same species (Franceschi, Cornet, et al. 2010; Hafer 2018), has converged across distantly related parasites (Hafer and Milinski 2016; Ponton et al. 2006) and rapidly responds to experimental selection (Hafer‐Hahmann 2019). Host phylogeny and the host parasite pair had a slightly larger but still small effect, suggesting only a limited impact of co‐evolution and host specific influence. This pattern was robust to different host phylogenies. Host manipulation likely exploits specific physiological or behavioural pathways (Herbison 2017; Li and Poulin 2024), which will vary among hosts. However, the absence of strong phylogenetic effects suggests that ecology may play the more important role in determining the strength of host manipulation. This fits well with the idea of host manipulation as an extended phenotype (Dawkins 1982), where parasite genes exert control beyond their body shaping the host phenotype, but are under selection to maximise parasite fitness.

In line with previous meta‐analyses (Fayard et al. 2020; McElroy and de Buron 2014), I find strong heterogeneity and strong variation between studies, likely reflecting differences in methodology and experimental context. Indeed, I cannot rule out that infection procedure (natural versus experimental) might have a minor impact on host manipulation, reinforcing previous findings in meta‐analyses looking at the effect of parasites on host traits related to some behaviours, but not directly focused on host manipulation (McElroy and de Buron 2014; Mrugała et al. 2023, but see Fayard et al. 2020). Additionally, environmental conditions, co‐infections, infection intensity, host nutritional status and abiotic factors (e.g., temperature) likely modulate manipulation strength (Hafer and Milinski 2015b; Labaude, Rigaud, and Frank 2015; McElroy and de Buron 2014; Thomas et al. 2011). Indeed, I do find clear context‐dependence in that host manipulation is more pronounced in the presence of a predator. This is consistent with cost–benefit theories (Ha 2010) and may help alleviate costs associated with suboptimal host behaviour when such behaviours are unlikely to result in any transmission. Publication bias remains a concern with regards to both published papers and results reported within published papers, with stronger effects overrepresented and non‐significant findings likely underrepresented. This emphasises the importance of preregistration, balanced reporting and open data in science in general and in studies on host manipulation in particular. By contrast, I detected no temporal decline in effect sizes. Such a trend was observed by Poulin (2000), but not in more recent meta‐analyses (Fayard et al. 2020; McElroy and de Buron 2014; Nakagawa et al. 2015) indicating that reported effect sizes have stabilised.

In mature parasites, there is some indication that manipulation strength may vary with the type of subsequent host. Parasites transmitted to birds showed the strongest manipulation, while those transmitted to terrestrial vertebrates the weakest and the ones transmitted to aquatic vertebrates exhibited intermediate levels. These patterns should, however, be interpreted cautiously, as bird parasites, especially those using aquatic birds, are particularly well studied. This could exaggerate apparent differences. Alternatively, systems showing more dramatic behavioural changes likely attract more attention, so unstudied systems may, on average, display weaker manipulation. Benesh et al. (2011) observed convergence of parasite growth in intermediate hosts based on the trophic link from intermediate to subsequent host rather than the type of subsequent host used. I find no such convergence with regard to host manipulation. Finer subsequent host (and possibly trophic link) classifications might clarify patterns, but small sample sizes for some comparisons would have limited statistical inference.

Contrary to theoretical expectations that predation pressure should shape the evolution of manipulation (Oliver and Best 2024; Poulin 1994b; Seppälä and Jokela 2008; Vickery and Poulin 2009; de Vries and van Langevelde 2018), I found no evidence linking these factors to manipulation strength. A lack of standardised measures for predation may have contributed to this result and species‐level proxies may fail to capture local variation in predation pressure or host manipulation. In addition, selection pressures on host manipulation may fluctuate over time due to changes in predator communities and co‐evolutionary dynamics (Oliver and Best 2024). Substantial unexplained variance in the data further suggests that key ecological or evolutionary drivers remain unidentified. Potential costs of manipulation were also unclear, that is, there was no clear correlation between manipulation strength and adult parasite size (used as a fitness proxy) that could hint at trade‐offs. Empirical support for such trade‐offs is mixed (Franceschi, Bollache, et al. 2010; Gopko et al. 2017; Hafer‐Hahmann 2019). Likewise, infection site did not clearly predict manipulation strength despite a previous meta‐analysis reporting associations between parasites in the body cavity and altered microhabitat choice and suggesting that infection site may influence the feasibility of host manipulation (Lafferty and Shaw 2013).

To fully understand host manipulation, we must link its evolutionary dynamics with the underlying mechanisms. Recent advances in omics technologies, increasingly applied to non‐model systems, are beginning to uncover the mechanistic basis of manipulation (Doherty and Matthews 2022; Herbison et al. 2018; Herbison 2017; Li and Poulin 2024). Yet, a comprehensive understanding of how host manipulation—and extended phenotypes more generally—evolve and function, especially within changing ecosystems, will require integrating mechanistic insights with evolutionary and ecological perspectives.

## Author Contributions

The author takes full responsibility for this article.

## Supporting information


**Figure S1:** Phylogenetic trees for parasites and host taxa included in this analysis. Numbers in brackets indicate the number of studies/number of observations for each taxon.

**Figure S2:** Diagnostic plots for the best model in each case. This model contained the standard error, parasite stage (A and B; all parasites) and predator type as fixed effects and paper ID, host phylogeny and parasite phylogeny and the interaction between host and parasite as random effects.

**Figure S3:** Estimated marginal means and marginal posterior distributions. Outliers removed. Higher (positive) effect sizes indicate an increase in predation susceptibility while lower (negative) effect sizes indicate a decrease in predation susceptibility (or increased predation suppression). *N* indicates number of observations (number of studies/number of unique host–parasite pairs). Error bars indicate 95% highest posterior density (HPD) interval. (A) Full model (all parasites); (B) Mature parasites; (C) Immature parasites.
**Figure S4:** Estimated marginal means and marginal posterior distributions (A‐C) and relationship between effect sizes and publication year (D). Higher (positive) effect sizes indicate an increase in predation susceptibility while lower (negative) effect sizes indicate a decrease in predation susceptibility (or increased predation suppression). *N* indicates number of observations (number of studies/number of unique host–parasite pairs). Error bars indicate 95% highest posterior density (HPD) interval. (A, B) Full model (all parasites); (C) Mature parasites; (D) Immature parasites.
**Figure S5:** Estimated marginal means and marginal posterior distributions for different types of behaviour. Higher (positive) effect sizes indicate an increase in predation susceptibility while lower (negative) effect sizes indicate a decrease in predation susceptibility (or increased predation suppression). *N* indicates number of observations (number of studies/number of unique host–parasite pairs). Error bars indicate 95% highest posterior density (HPD) interval. (A) Full model (all parasites); (B) Mature parasites; (C) Immature parasites.
**Figure S6:** Funnel plots for the complete data set (A, B), a data set containing only data from mature parasites (C, D) and a data set containing only data from immature parasites (E, F). In panels (A, C and E) all data was included, in panels (B, D and F) outliers were excluded (see Section 2 for criteria used to identify outliers). Dotted line indicates the mean from each data set, white are indicates the 95% pseudo confidence interval. Plots were created using R package metaphor.
**Figure S7:** Relationship between effect size (Cohen's *d*) and its Standard error (SE) in models without outliers. Outliers removed. Higher (positive) effect sizes indicate an increase in predation susceptibility while lower (negative) effect sizes indicate a decrease in predation susceptibility (or increased predation suppression). (A) Full model (all parasites); (B) Mature parasites; (C) Immature parasites.
**Figure S8:** Estimated marginal means and marginal posterior distributions for different subsequent hosts (A), infection sites (C) and habitat (D) and relationship between effect size and adult parasite size (B; proxy for parasite fitness). Higher (positive) effect sizes indicate an increase in predation susceptibility while lower (negative) effect sizes indicate a decrease in predation susceptibility (or increased predation suppression). *N* indicates number of observations (number of studies/number of unique host parasite pairs). Error bars indicate 95% highest posterior density (HPD) interval. Trend line indicates estimate and 96% HPD range. Mature parasites only.
**Figure S9:** Estimated marginal means and marginal posterior distributions for different behaviours by subsequent host type. Higher (positive) effect sizes indicate an increase in predation susceptibility while lower (negative) effect sizes indicate a decrease in predation susceptibility (or increased predation suppression). *N* indicates number of observations (number of studies/number of unique host–parasite pairs). Error bars indicate 95% highest posterior density (HPD) interval.

**Figure S10:** Phylogenetic trees host taxa included in this analysis. Numbers in brackets indicate the number of studies/number of observations for each taxon. Tree based on open tree of life (see methods in main manuscript). Major arthropod taxa were re‐arranged based on Chang and Lai (2018), GIGA Community of Scientists (2014), Thomas et al. (2020).
**Table S14:** Heterogeneity (A) and proportion of variance explained by random effects (B) for different models using an alternative host phylogeny.
**Table S13:** Results of model comparisons using leave‐one‐out cross‐validation (LOO) using an alternative host phylogeny. Results were obtained through the Bayesian modelling framework (BRMS). Factors that clearly improved the model are indicated in bold (i.e., ΔELPD larger than two times SE), those for which an improvement was observed, but was unclear due to ΔELPD being between one and two times SE are highlighted in italics. Comparisons are always to the preceding model that was clearly better (i.e., highlighted in bold). All models included the following random effects: Paper ID, Host and parasite phylogeny and the interaction between host and parasite (see Table S14 for information on heterogeneity and the proportion of variance explained by each random effect). R‐hat was close to 1 (< 1.01) for all models.
**Table S15:** Estimated marginal means for the best model.

**Table S1:** Overview of host and parasite taxa included in the meta‐analysis for each parasite stage. *N* indicated number of observations/number of studies for each taxon. See Figure S1 for a phylogenetic tree of the species included in this meta‐analysis.

**Table S2:** Additional traits investigated.
**Table S3:** Model diagnostics. Please see Table 2 for ELPD loo and model comparisons. R‐hat was close to 1 (< 1.01) or all models. The number of problematic pareto k values never exceeded 2% of all observations.
**Table S4:** Model diagnostics for models investigating factors that may drive or constrain the evolution of host manipulation. Please see Table 4 for ELPD loo and model comparisons. R‐hat was close to 1 (< 1.01) for all models. The number of problematic pareto k values never exceeded 2% of all observations.
**Table S5:** Model diagnostics for models investigating the interaction between behaviour and factors that may drive or constrain the evolution of host manipulation. Loo was obtained with moment match set to true. Please see Table S11 for ELPD loo and model comparisons. The number of problematic pareto k values never exceeded 3% of all observations. For immature parasites predator was included for the basic model but not for the one without outliers since it did not clearly improve that model.
**Table S6:** Results of model comparisons using leave‐one‐out cross‐validation (LOO) and model diagnostics for predation susceptibility. Results were obtained through the Bayesian modelling framework (BRMS). Factors that clearly improved the model are indicated in bold (i.e., difference in ELPD larger than two times SE), those for which an improvement was observed, but was unclear due to the difference in ELPD being between one and two times SE are highlighted in italics. Comparisons are always to the preceding model that was clearly better (i.e., highlighted in bold). All models included the following random effects: Paper ID, Host and parasite phylogeny and the interaction between host and parasite. R‐hat was close to 1 (< 1.01) for all models. Please note that a substantial number of problematic pareto k values were occurred. Hence ELPD loo may be unreliable and any comparison should be viewed with caution.
**Table S7:** Estimated marginal means and contrasts for the best model. Outliers removed.
**Table S8:** Estimated marginal means and contrasts. Interaction between predator stage and type of predator; full model on all parasite stages.
**Table S9:** Estimated marginal means and contrasts. Models include parasite stage (full model on all parasites only), behaviour and predator type as fixed effects.
**Table S10:** Estimated marginal means and contrasts for type of next host (A), adult parasite size (B), infection site (C) and host habitat (D). Effects were only observed for mature parasites. For information on other factors included in model see Table 1B.
**Table S11:** Results of model comparisons using leave‐one‐out cross‐validation (LOO). Behaviour was retained in all models. Results were obtained through the Bayesian modelling framework (BRMS). Factors that clearly improved the model are indicated in bold (i.e., difference in ELPD larger than two times SE), those for which an improvement was observed, but was unclear due to the difference in ELPD being between one and two times SE are highlighted in italics. Comparisons are always to the model indicated in bold. For immature parasites predator was included for the basic model but not for the one without outliers since it did not clearly improve that model. All models included the following random effects: Paper ID, Host and parasite phylogeny and the interaction between host and parasite. Please refer to Table S5 for model diagnostics.
**Table S12:** Estimated marginal means and contrasts for the interaction between behaviour and next host. Please note that only comparisons involving the same type of behaviour are included in Table S8. Results were obtained through the Bayesian modelling framework (BRMS). Factors that clearly improved the model are indicated in bold (i.e., difference in ELPD larger than two times SE), those for which an improvement was observed, but was unclear due to the difference in ELPD being between one and two times SE are highlighted in italics. Comparisons are always to the preceding model that was clearly better (i.e., highlighted in bold). All models included the following random effects: Paper ID, Host and parasite phylogeny and the interaction between host and parasite.

## Data Availability

Data and code are available through zenodo (doi: https://doi.org/10.5281/zenodo.17200384).
