# Supplementary material for: The Evolution and Ecology of Host Manipulation in Helminth Parasites: A Phylogenetic Meta‐Analysis
Source: Ecol Lett. 2026 Feb 18;29(2):e70340. doi: 10.1111/ele.70340 (PMC12916080; doi:10.1111/ele.70340)
Supplement: Supplementary file 1 — Figure S1: Phylogenetic trees for parasites and host taxa included in this analysis. Numbers in brackets indicate the number of studies/number of observations for each taxon. [file ELE-29-0-s002.pdf]

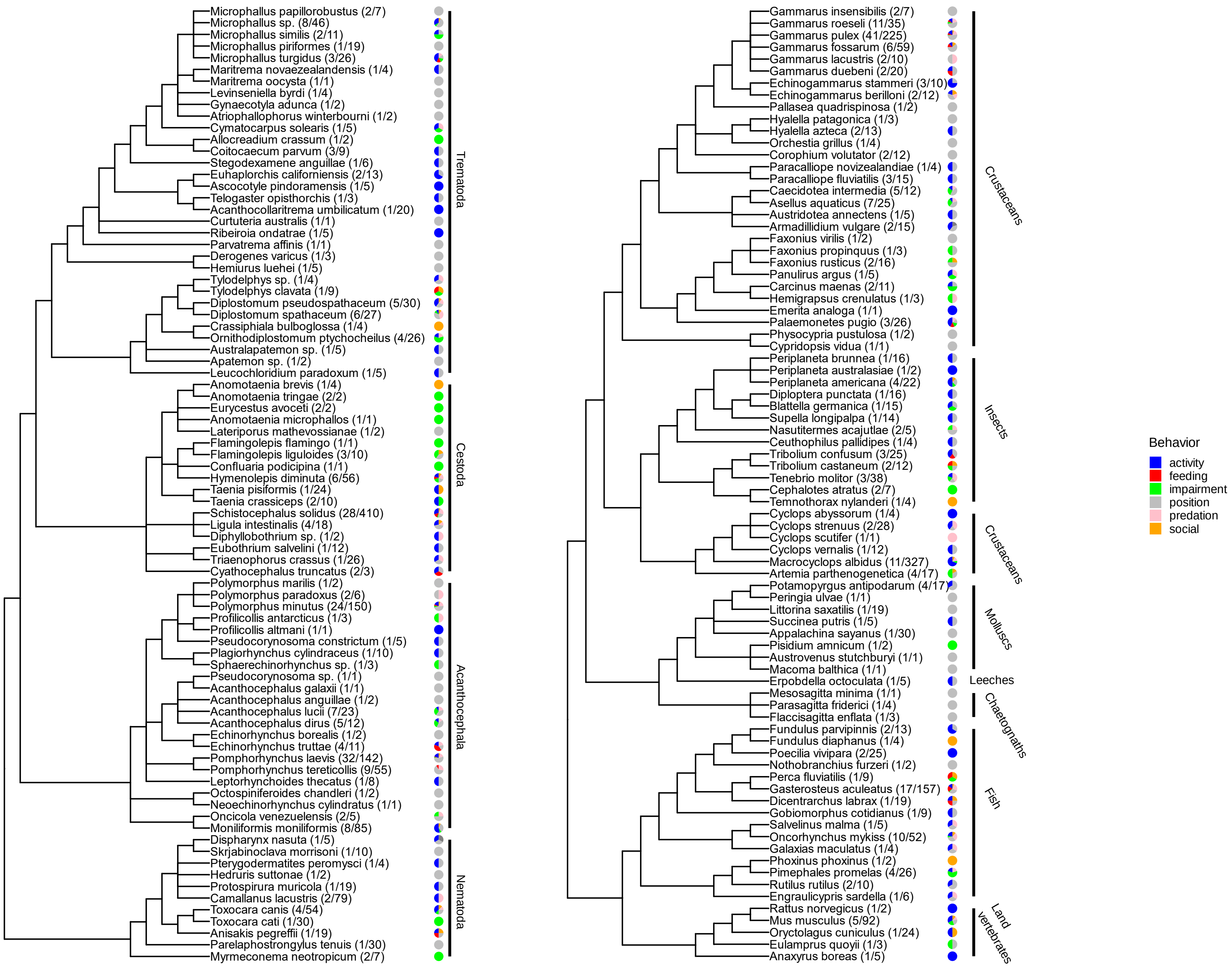

Figure S1: Phylogenetic trees for parasites and host taxa included in this analysis. Numbers in brackets indicate the number of studies/ number of observations for each taxon.
