# Supplementary material for: The Evolution and Ecology of Host Manipulation in Helminth Parasites: A Phylogenetic Meta‐Analysis
Source: Ecol Lett. 2026 Feb 18;29(2):e70340. doi: 10.1111/ele.70340 (PMC12916080; doi:10.1111/ele.70340)
Supplement: Supplementary file 2 — Figure S2: Diagnostic plots for the best model in each case. This model contained the standard error, parasite stage (A and B; all parasites) and predator type as fixed effects and paper ID, host phylogeny and parasite phylogeny and the interaction between host and parasite as random effects. [file ELE-29-0-s001.pdf]

## A: All parasites

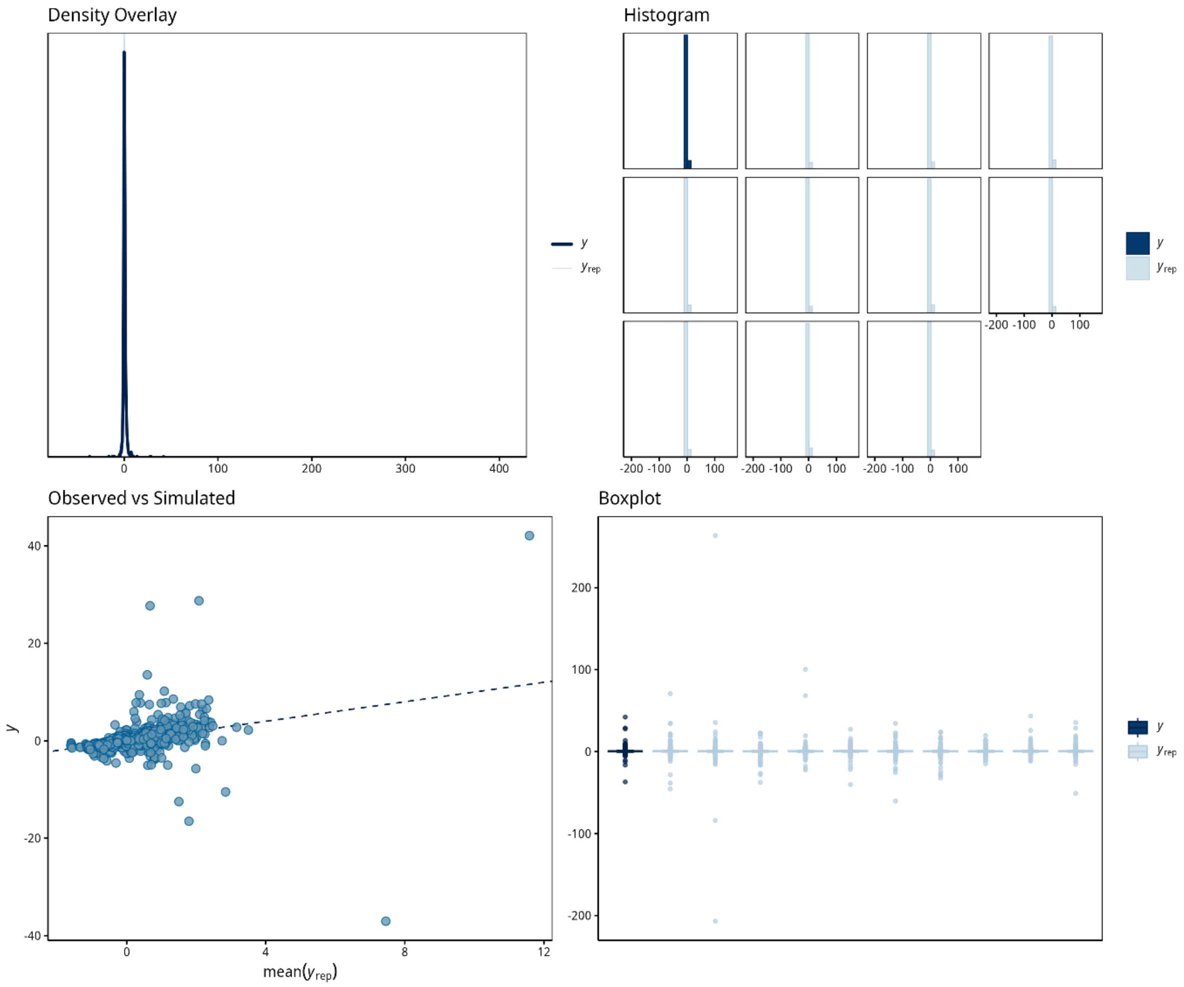

## B: All parasites; outliers removed

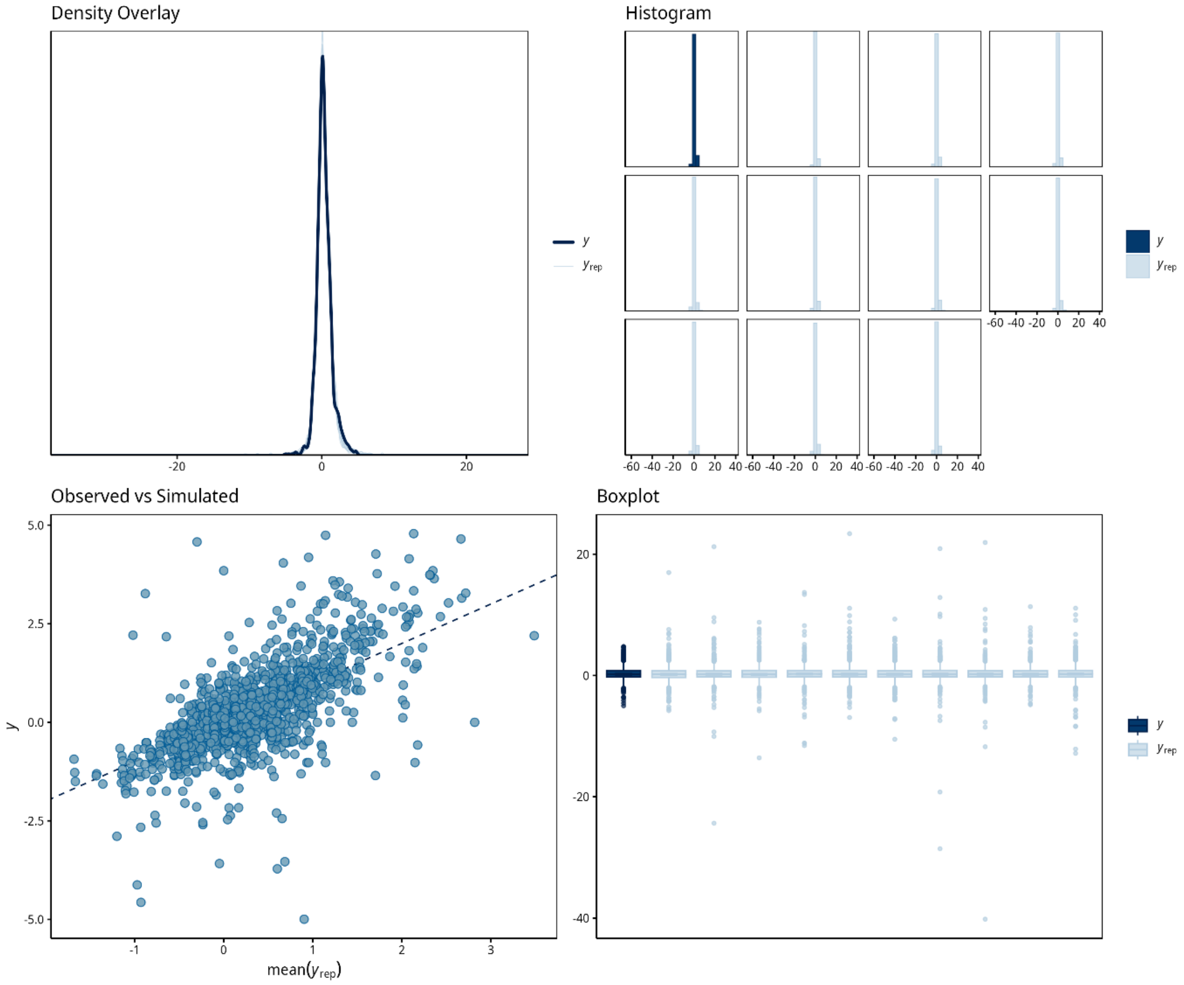

## C: Mature parasites

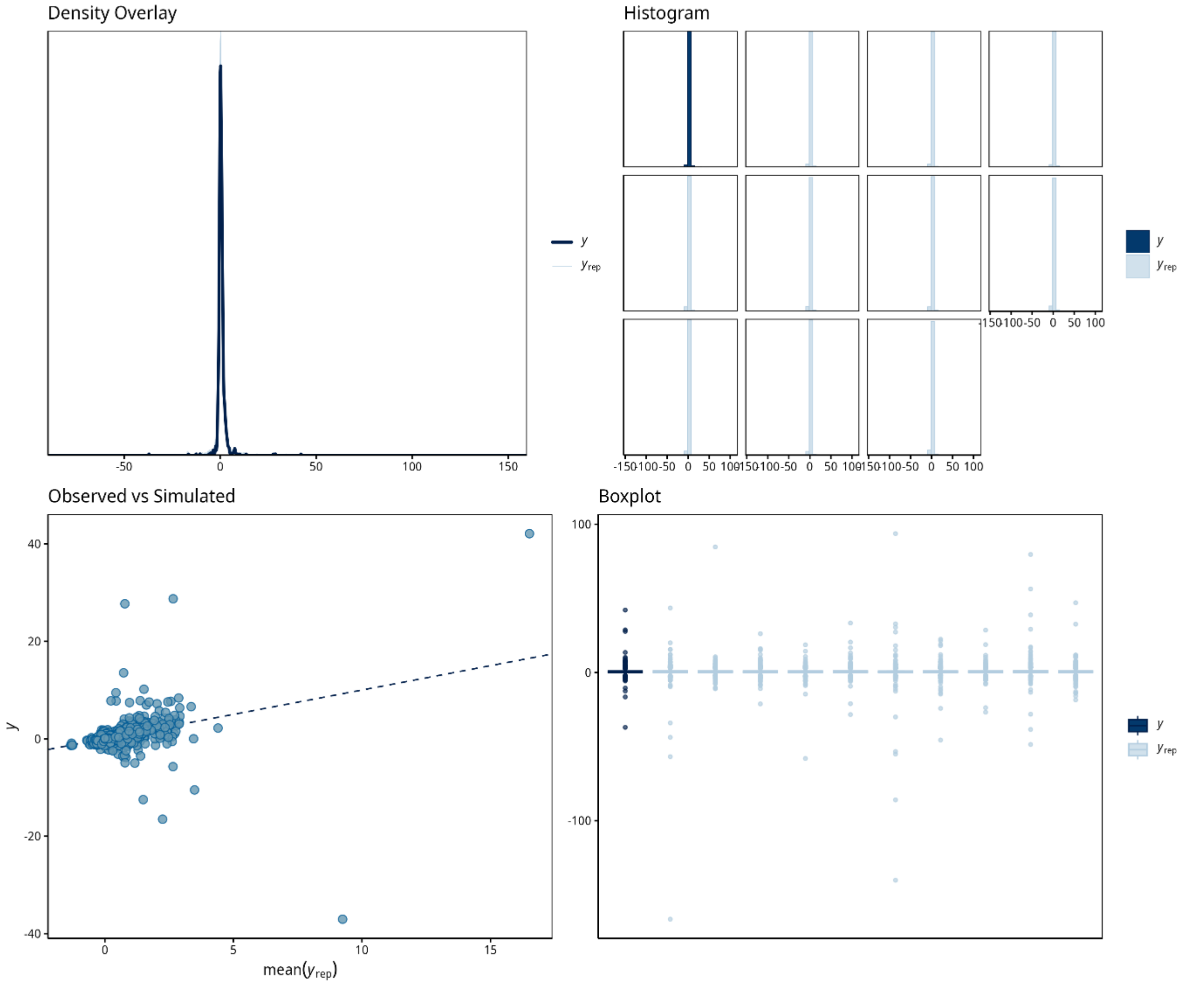

## D: Mature parasites; outliers removed

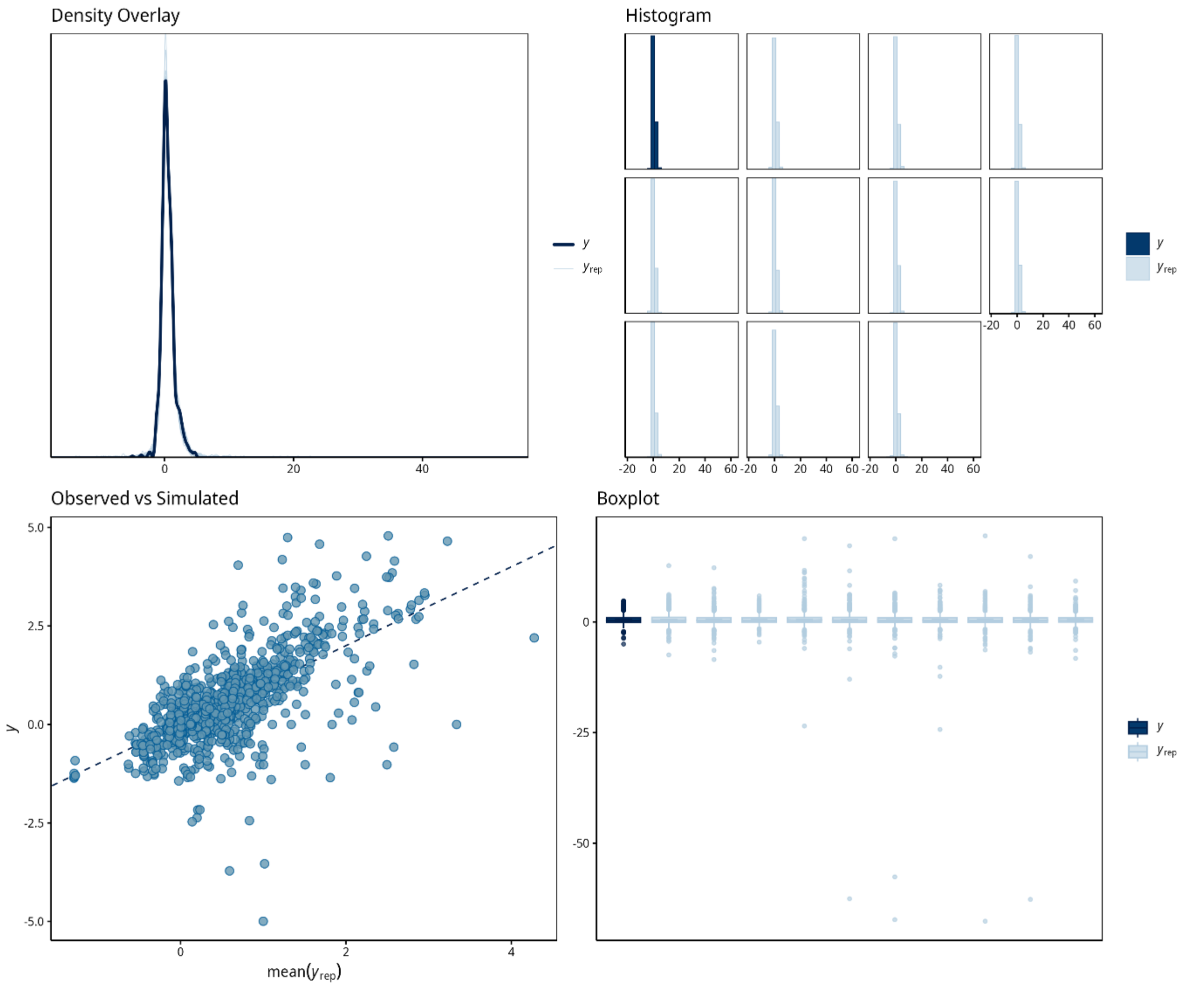

## E: Immature parasites

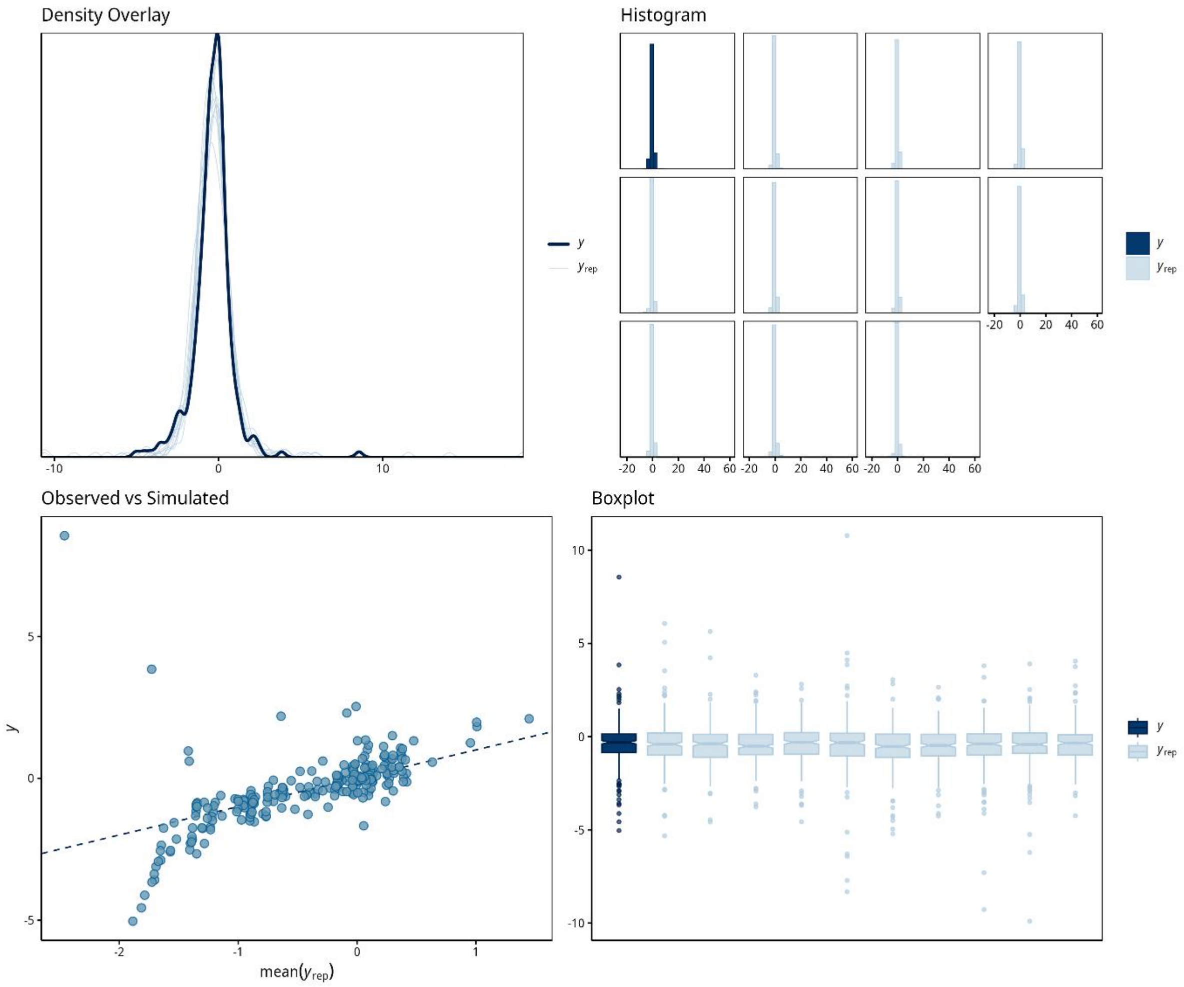

## F: Immature parasites; outliers removed

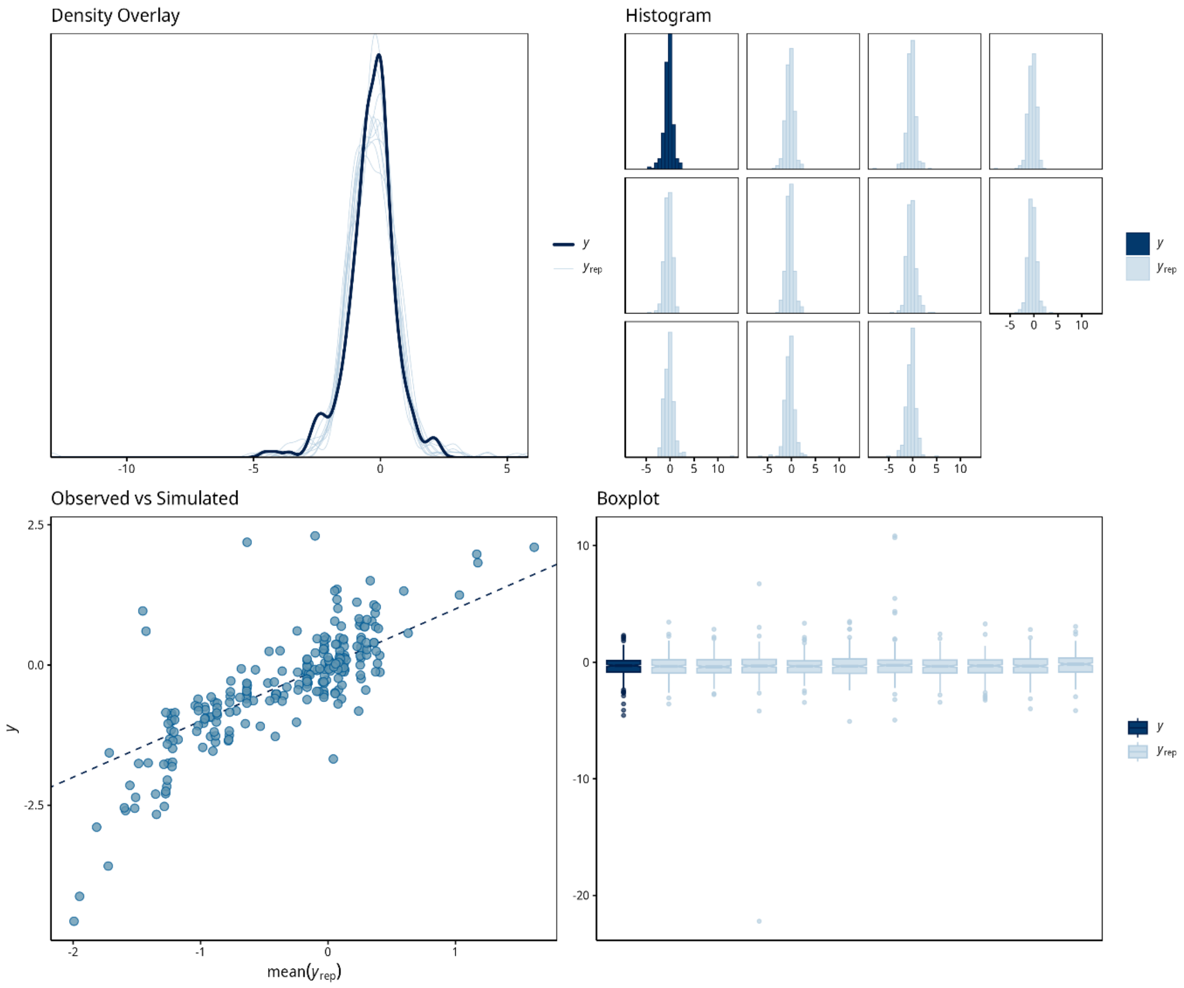


**Figure S2: Diagnostic plots for the best model in each case.** This model contained the standard error, parasite stage (A and B; all parasites), and predator type as fixed effects and paper ID, host phylogeny and parasite phylogeny and the interaction between host and parasite as random effects.
