# Supplementary material for: The Evolution and Ecology of Host Manipulation in Helminth Parasites: A Phylogenetic Meta‐Analysis
Source: Ecol Lett. 2026 Feb 18;29(2):e70340. doi: 10.1111/ele.70340 (PMC12916080; doi:10.1111/ele.70340)
Supplement: Supplementary file 3 — Figure S3: Estimated marginal means and marginal posterior distributions. Outliers removed. Higher (positive) effect sizes indicate an increase in predation susceptibility while lower (negative) effect sizes indicate a decrease in predation susceptibility (or increased predation suppression). N indicates number of observations (number of studies/number of unique host–parasite pairs). Error bars indicate 95% highest posterior density (HPD) interval. (A) Full model (all parasites); (B) Mature parasites; (C) Immature parasites. Figure S4: Estimated marginal means and marginal posterior distributions (A‐C) and relationship between effect sizes and publication year (D). Higher (positive) effect sizes indicate an increase in predation susceptibility while lower (negative) effect sizes indicate a decrease in predation susceptibility (or increased predation suppression). N indicates number of observations (number of studies/number of unique host–parasite pairs). Error bars indicate 95% highest posterior density (HPD) interval. (A, B) Full model (all parasites); (C) Mature parasites; (D) Immature parasites. Figure S5: Estimated marginal means and marginal posterior distributions for different types of behaviour. Higher (positive) effect sizes indicate an increase in predation susceptibility while lower (negative) effect sizes indicate a decrease in predation susceptibility (or increased predation suppression). N indicates number of observations (number of studies/number of unique host–parasite pairs). Error bars indicate 95% highest posterior density (HPD) interval. (A) Full model (all parasites); (B) Mature parasites; (C) Immature parasites. Figure S6: Funnel plots for the complete data set (A, B), a data set containing only data from mature parasites (C, D) and a data set containing only data from immature parasites (E, F). In panels (A, C and E) all data was included, in panels (B, D and F) outliers were excluded (see Section 2 for criteria used to identify outliers). Dot [file ELE-29-0-s006.pdf]

Supplementary figures

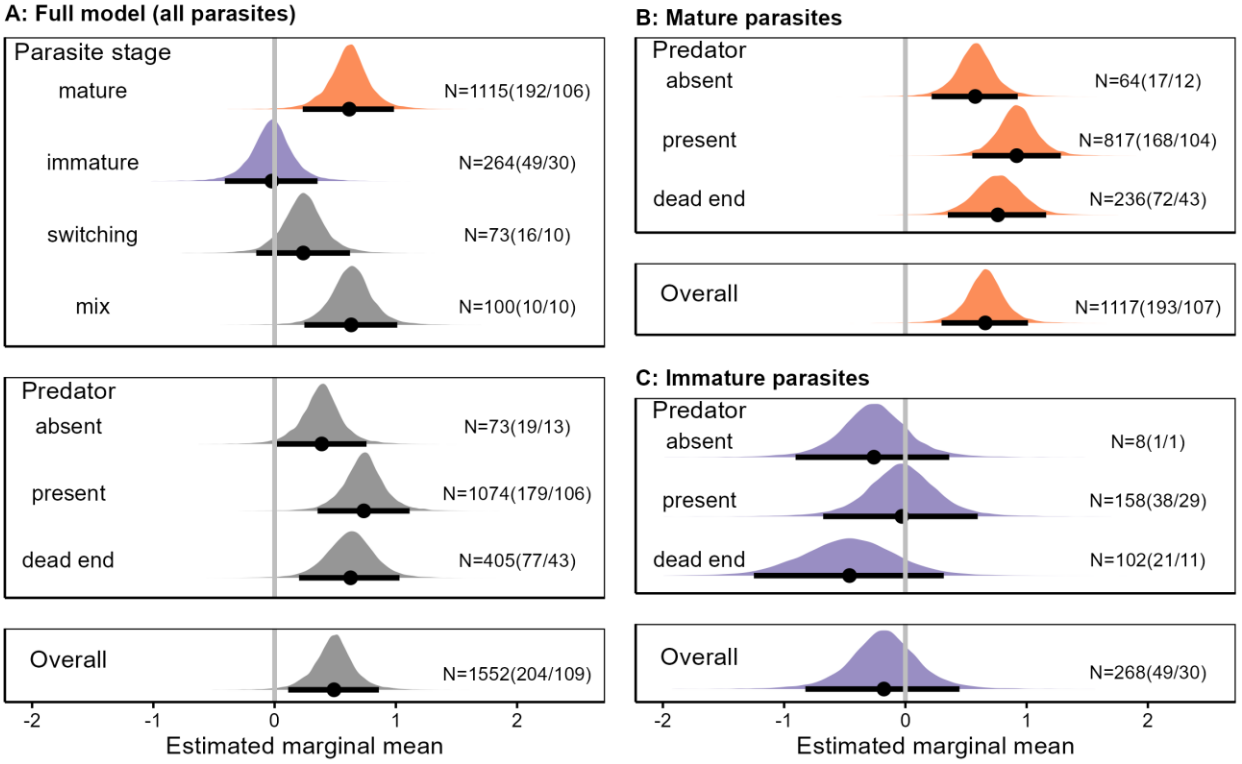

Figure S3: Estimated marginal means and marginal posterior distributions. Outliers removed. Higher (positive) effect sizes indicate an increase in predation susceptibility while lower (negative) effect sizes indicate a decrease in predation susceptibility (or increased predation suppression). N indicates number of observations (number of studies / number of unique host-parasite pairs). Error bars indicate 95% highest posterior density (HPD) interval. A: Full model (all parasites); B: Mature parasites; C: Immature parasites.

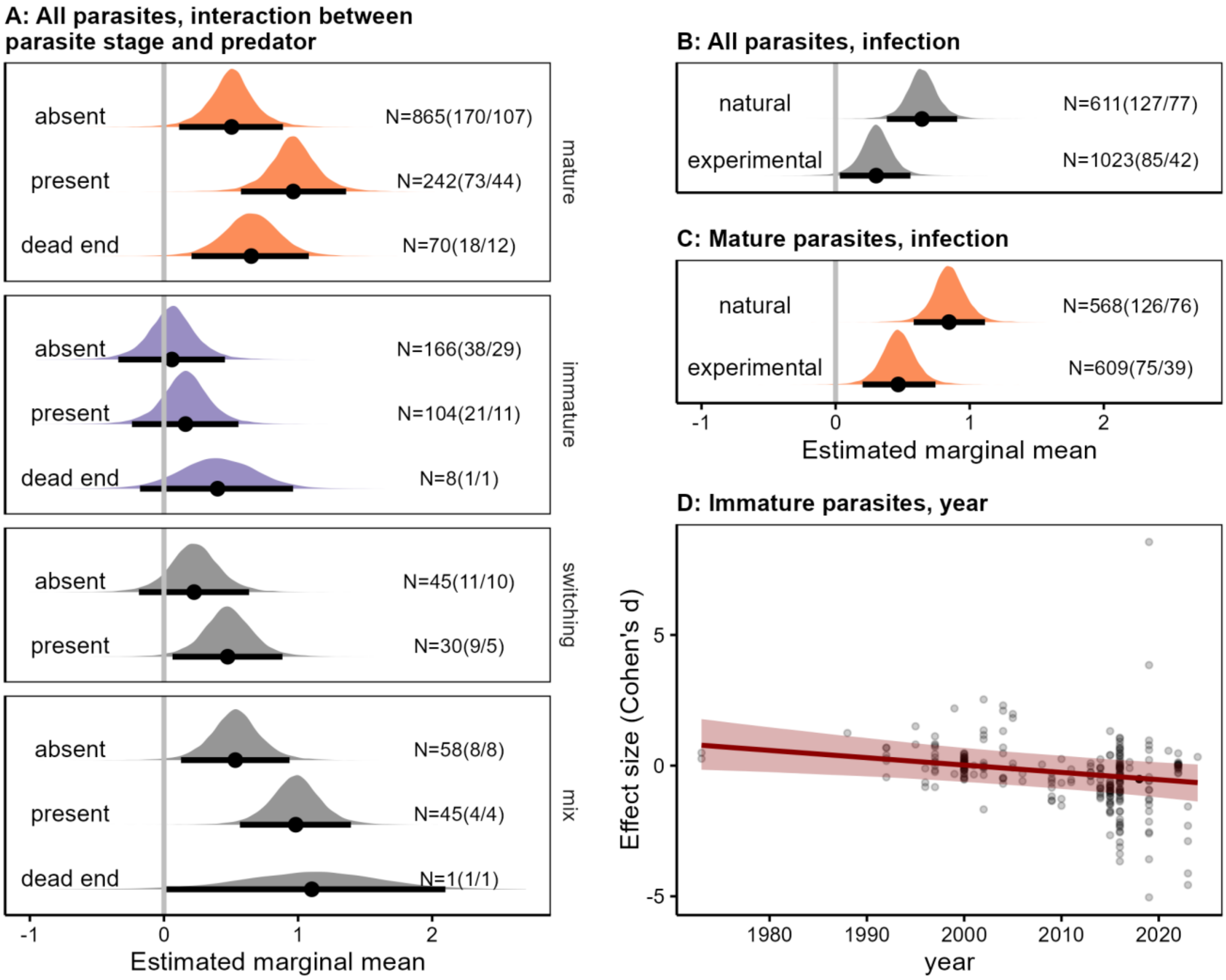

Figure S4: Estimated marginal means and marginal posterior distributions (A-C) and relationship between effect sizes and publication year (D). Higher (positive) effect sizes indicate an increase in predation susceptibility while lower (negative) effect sizes indicate a decrease in predation susceptibility (or increased predation suppression). N indicates number of observations (number of studies / number of unique host-parasite pairs). Error bars indicate 95% highest posterior density (HPD) interval. A-B: Full model (all parasites); C: Mature parasites; D: Immature parasites.

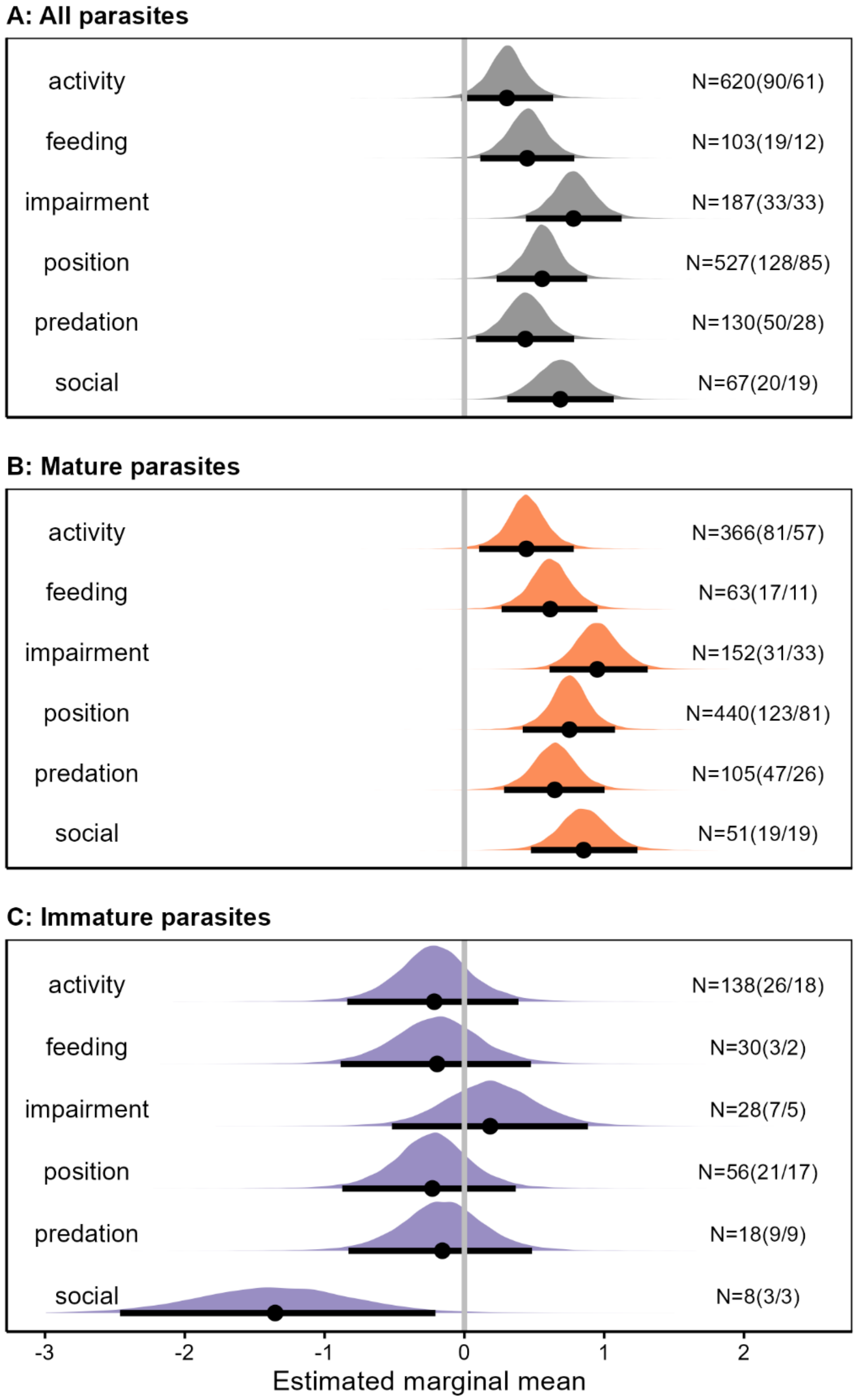

Figure S5: Estimated marginal means and marginal posterior distributions for different types of behavior. Higher (positive) effect sizes indicate an increase in predation susceptibility while lower (negative) effect sizes indicate a decrease in predation susceptibility (or increased predation suppression). N indicates number of observations (number of studies / number of unique host-parasite pairs). Error bars indicate 95% highest posterior density (HPD) interval. A: Full model (all parasites); B: Mature parasites; C: Immature parasites.

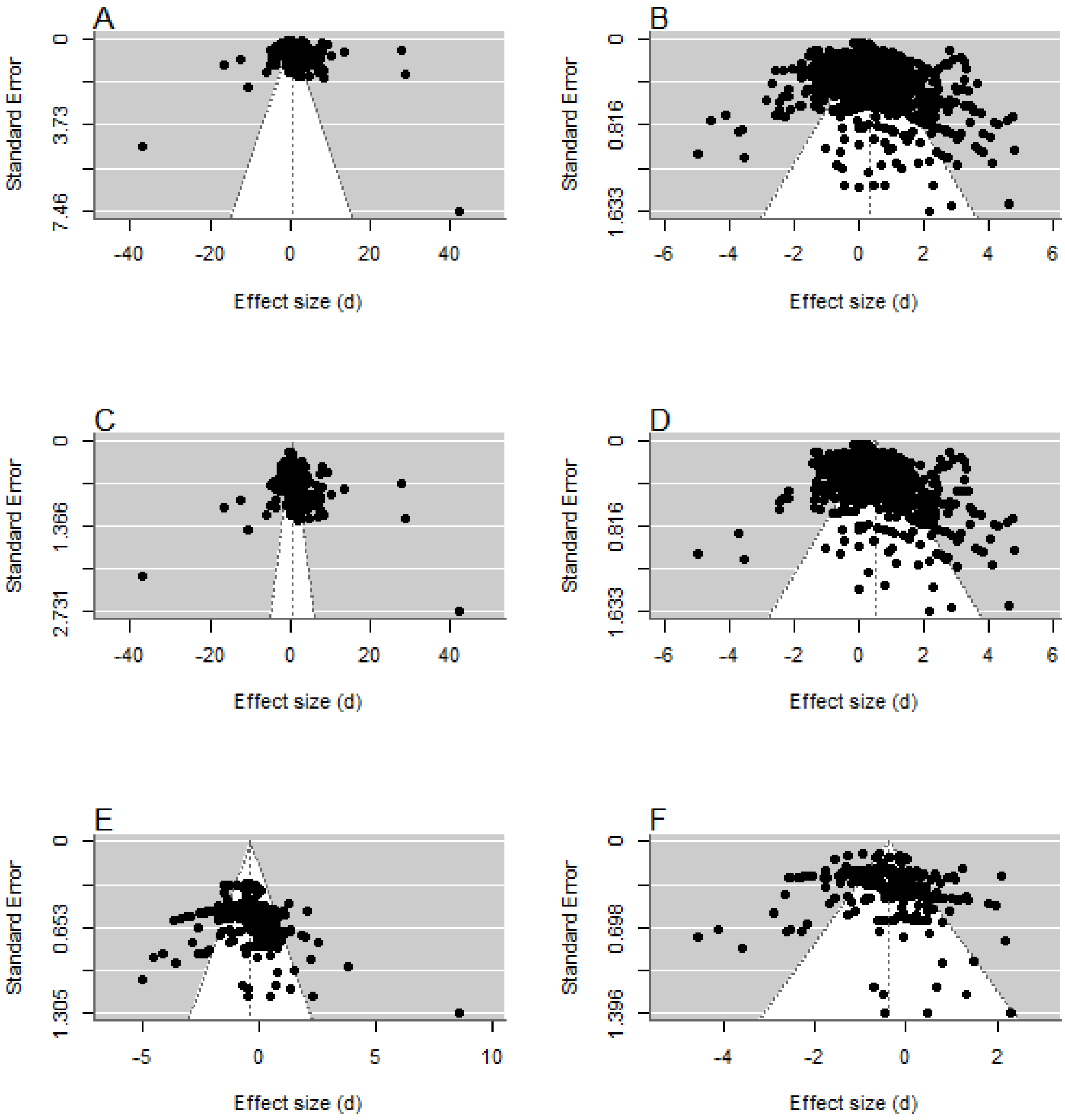

Figure S6: Funnel plots for the complete data set (A & B), a data set containing only data from mature parasites (C & D), and a data set containing only data from immature parasites (E & F). In panels A, C, and E all data was included, in panels B, D, and F outliers were excluded (see methods section for criteria used to identify outliers). Dotted line indicates the mean from each data set, white are indicates the 95% pseudo confidence interval. Plots were created using R package metaphor.

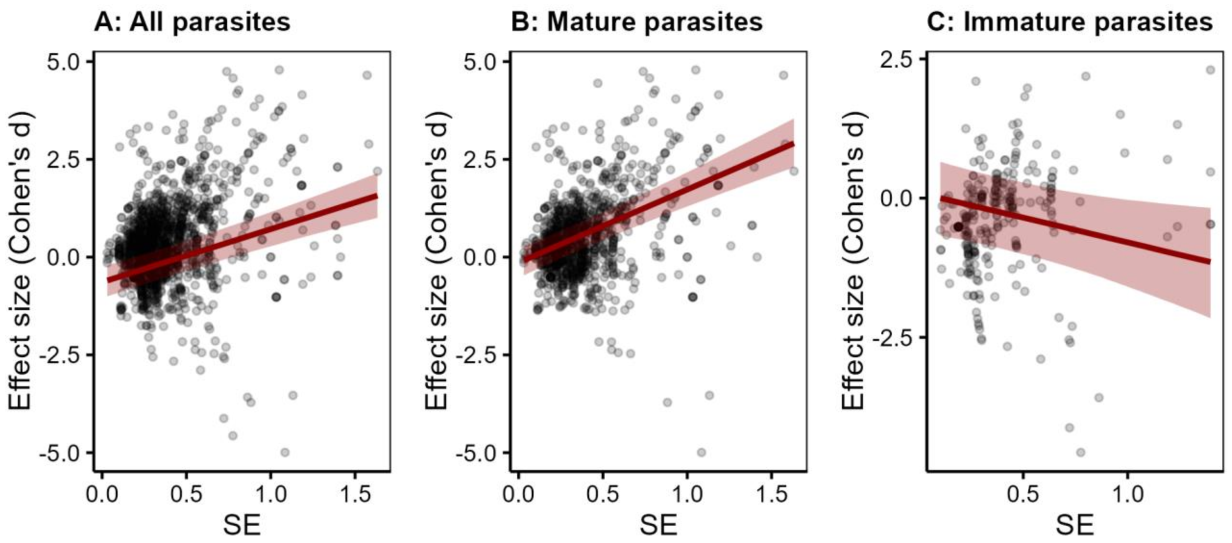

Figure S7: Relationship between effect size (Cohen's d) and its Standard error (SE) in models without outliers. Outliers removed. Higher (positive) effect sizes indicate an increase in predation susceptibility while lower (negative) effect sizes indicate a decrease in predation susceptibility (or increased predation suppression). A: Full model (all parasites); B: Mature parasites; C: Immature parasites.

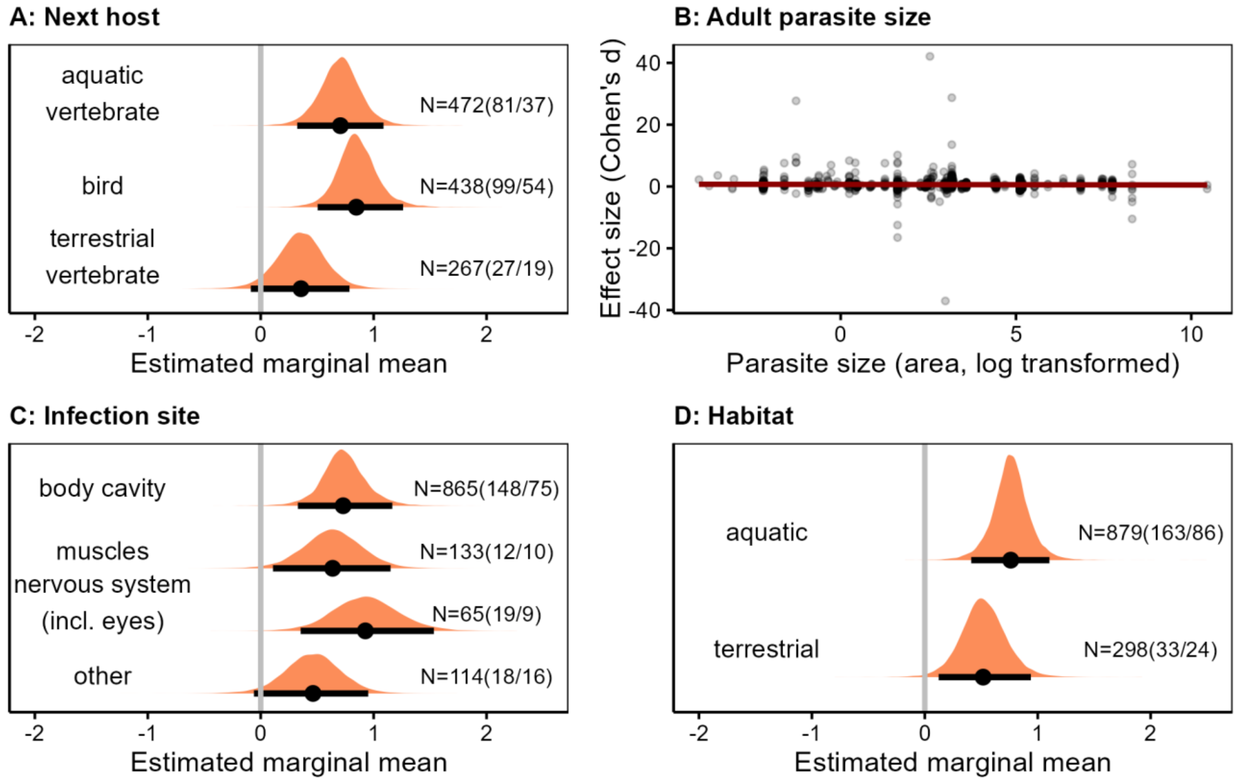

Figure S8: Estimated marginal means and marginal posterior distributions for different subsequent hosts (A), infection sites (C), and habitat (D) and relationship between effect size and adult parasite size (B; proxy for parasite fitness). Higher (positive) effect sizes indicate an increase in predation susceptibility while lower (negative) effect sizes indicate a decrease in predation susceptibility (or increased predation suppression). N indicates number of observations (number of studies / number of unique host-parasite pairs). Error bars indicate 95% highest posterior density (HPD) interval. Trend line indicates estimate and 96% HPD range. Mature parasites only.

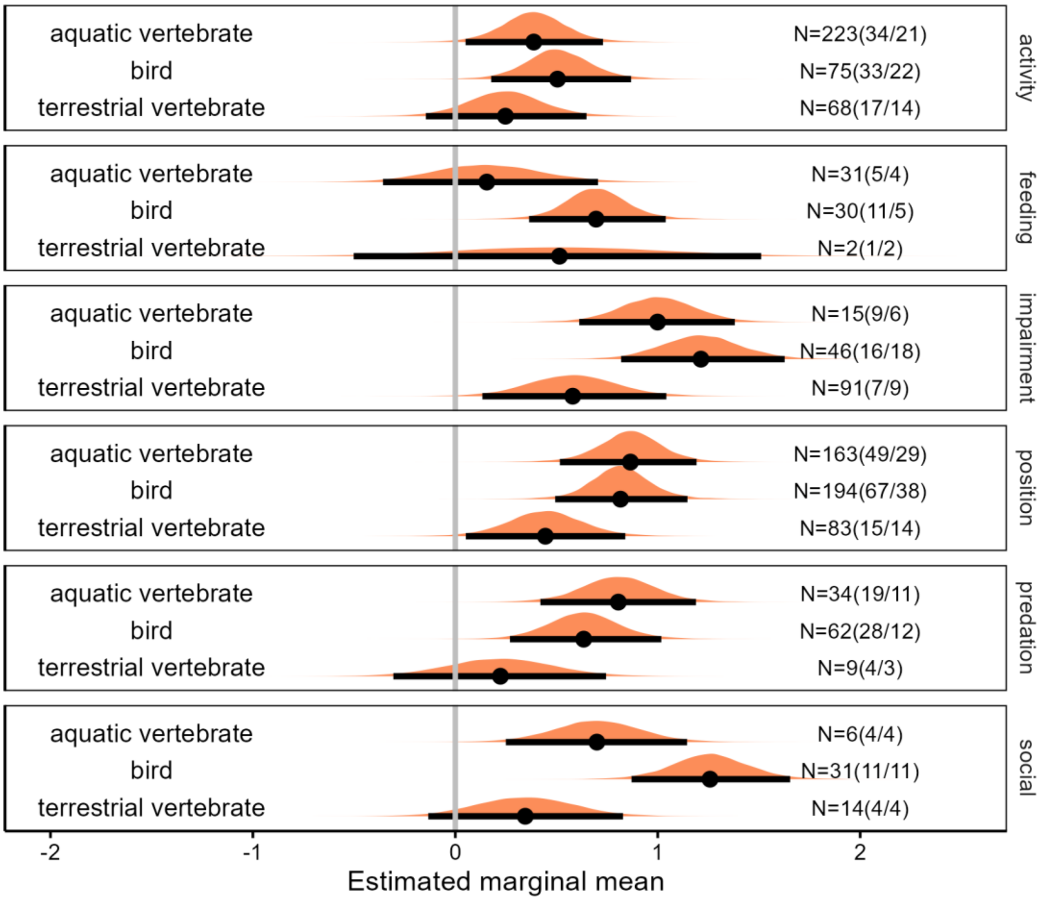

Figure S9: Estimated marginal means and marginal posterior distributions for different behaviors by subsequent host type. Higher (positive) effect sizes indicate an increase in predation susceptibility while lower (negative) effect sizes indicate a decrease in predation susceptibility (or increased predation suppression). N indicates number of observations (number of studies / number of unique host-parasite pairs). Error bars indicate 95% highest posterior density (HPD) interval.
