# Supplementary material for: The Evolution and Ecology of Host Manipulation in Helminth Parasites: A Phylogenetic Meta‐Analysis
Source: Ecol Lett. 2026 Feb 18;29(2):e70340. doi: 10.1111/ele.70340 (PMC12916080; doi:10.1111/ele.70340)
Supplement: Supplementary file 4 — Figure S10: Phylogenetic trees host taxa included in this analysis. Numbers in brackets indicate the number of studies/number of observations for each taxon. Tree based on open tree of life (see methods in main manuscript). Major arthropod taxa were re‐arranged based on Chang and Lai (2018), GIGA Community of Scientists (2014), Thomas et al. (2020). Table S14: Heterogeneity (A) and proportion of variance explained by random effects (B) for different models using an alternative host phylogeny. Table S13: Results of model comparisons using leave‐one‐out cross‐validation (LOO) using an alternative host phylogeny. Results were obtained through the Bayesian modelling framework (BRMS). Factors that clearly improved the model are indicated in bold (i.e., ΔELPD larger than two times SE), those for which an improvement was observed, but was unclear due to ΔELPD being between one and two times SE are highlighted in italics. Comparisons are always to the preceding model that was clearly better (i.e., highlighted in bold). All models included the following random effects: Paper ID, Host and parasite phylogeny and the interaction between host and parasite (see Table S14 for information on heterogeneity and the proportion of variance explained by each random effect). R‐hat was close to 1 (< 1.01) for all models. Table S15: Estimated marginal means for the best model. [file ELE-29-0-s004.pdf]

Supplementary methods and results: Alternative host phylogeny

Host and parasite phylogenies were based on data from the Open Tree of Life (Redelings, B *et al.* 2019). Alternative phylogenies with regards to Arthropods have been proposed which vary with regards to the placement of Crustacea and Hexapoda and the phylogeny of Crustacea (e.g. Chang & Lai 2018, GIGA Community of Scientists 2014, Thomas et al. 2020, but see Bernot et al. 2023). Since these groups make up a large fraction of host species studied in this meta-analysis, I ran my main models for each subset (full model, mature parasites, and immature parasites) using a different host phylogeny (see Figure S10). Except for using a different phylogenetic tree for host species, these models were build and analysed as described in the method section.

In models using an alternative arthropod phylogeny the proportion of variance explained by host phylogeny (and other random effects) were similar to the models reported in the main manuscript (Table S13). Likewise, host manipulation was associated with the same factors in these models (Table S14) and estimates were in a similar range (Table S15) as in the models reported in the main text.

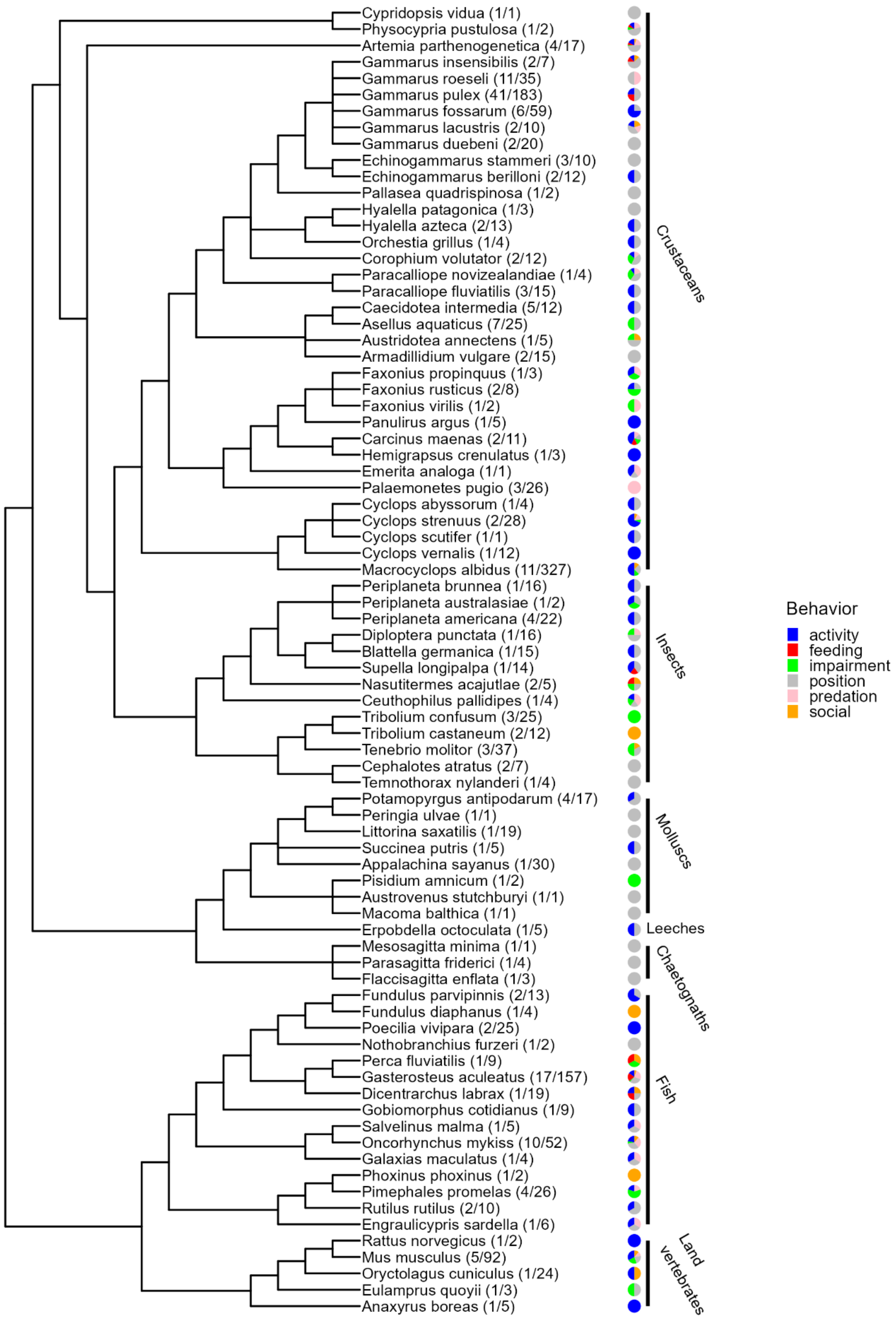

Figure S10: Phylogenetic trees host taxa included in this analysis. Numbers in brackets indicate the number of studies/ number of observations for each taxon. Tree based on open tree of life (see methods in main manuscript). Major arthropod taxa were re-arranged based on Chang et al. 2018, GIGA Community of Scientists 2014, Thomas et al. 2020.

Table S14: Heterogeneity (A) and proportion of variance explained by random effects (B) for different models using an alternative host phylogeny.

A: Variation explained by each random effect					
**model**	**interaction (host-parasite pair)**	**paper**	**parasite phylogeny**	**host phylogeny**	**residual**
all parasites	1.0%	10.7%	0.3%	5.2%	82.8%
mature parasites	1.4%	13.6%	0.7%	3.1%	81.2%
immature parasites	19.4%	10.6%	3.2%	5.0%	61.9%
**B: Heterogeneity (I^2)**					
**model**	**host phylogeny**	**interaction (host-parasite-pair)**	**paper**	**parasite phylogeny**	**overall**
all parasites	19% (0.21%-47.91%)	4.21% (0.08%-12.86%)	36.08% (21.44%-50.79%)	1.77% (0%-10.52%)	61.06% (49.03%-75%)
mature parasites	11.78% (0.04%-38.58%)	4.98% (0.34%-13.7%)	42.53% (27.36%-55.38%)	3.03% (0%-15.24%)	62.33% (52.62%-73.94%)
immature parasites	12.24% (0.01%-62.23%)	37.69% (4.15%-64.95%)	20.31% (5.38%-43.25%)	8.04% (0.01%-38.39%)	78.29% (63.61%-90.29%)

Table S13: Results of model comparisons using Leave-One-Out Cross-Validation (LOO) using an alternative host phylogeny. Results were obtained through the Bayesian modeling framework (BRMS). Factors that clearly improved the model are indicated in bold (i.e. ΔELPD larger than two times SE), those for which an improvement was observed, but was unclear due to ΔELPD being between one and two times SE are highlighted in italics. Comparisons are always to the preceding model that was clearly better (i.e. highlighted in bold). All models included the following random effects: Paper ID, Host and parasite phylogeny and the interaction between host and parasite (see Table S14 for information on heterogeneity and the proportion of variance explained by each random effect). R-hat was close to 1 (<1.01) for all models.

Model	ELPD loo	p loo	ΔELPD	Δse	Bulk ESS, min	Bulk ESS, mean	N pareto k >0.7 (k>1)	Max. pareto k
**A: All parasites**								
SE + parasite stage + predator + predator:parasite stage	*-2010*	*325.4*	*10.7*	*7.0*	*2496*	*18142*	*4 (2)*	*1.01*
**SE + parasite stage + predator**	**-2020**	**319.8**	**39.3**	**12.1**	**2837**	**19326**	**2**	**0.80**
SE + parasite stage + behavior	*-2043*	*291.5*	*16.9*	*10.1*	*2283*	*14762*	*4*	*0.76*
SE + parasite stage + infection	*-2054*	*303.4*	*5.6*	*3.3*	*3199*	*11454*	*5*	*0.73*
SE + parasite stage + year	-2057	307.3	3.1	3.6	2539	14404	0	0.55
**SE + parasite stage**	**-2060**	**304.4**	**72.8**	**20.1**	**2358**	**16801**	**7**	**0.95**
**SE**	**-2132**	**333.3**	**22.2**	**11.0**	**2825**	**11502**	**4**	**0.83**
1	-2155	347.5			1813	8108	3 (1)	1.38
**B: Mature parasites**								
**SE + predator**	**-1474**	**285.6**	**24.7**	**8.7**	2271	10550	0	0.69
SE + behavior	*-1478*	*277.6*	*20.6*	*11.1*	2790	10377	0	0.55
SE + infection	*-1492*	*282.5*	*6.6*	*3.4*	4129	10970	6	0.96
SE + year	-1497	286.6	1.7	2.8	2610	9144	0	0.61
**SE**	**-1499**	**286.3**	**33.6**	**12.4**	2606	10398	3	0.92
1	-1532	293.0			2712	13637	7 (1)	1.22
**C: Immature parasites**								
**SE + predator**	**-278**	**74.6**	**10.1**	**4.0**	4612	14987	1	0.77
SE + behavior	-288	74.2	0.7	3.8	4055	15267	0	0.60
SE + infection	-287	73.4	0.9	1.5	3815	13257	0	0.61
SE + year	*-285*	*72.6*	*3.5*	*2.1*	3598	10881	0	0.62
SE	*-288*	*75.0*	*3.8*	*2.9*	3358	9786	0	0.69
1	-292	71.7			3262	8424	0	0.50

ELPD loo: Expected log predictive density estimated via LOO cross-validation; p loo: Effective number of parameters, indicating model complexity; ΔELPD: Difference in ELPD loo between models; Δse: Standard error of ΔELPD, indicating uncertainty in model differences. Number of samples/ pareto k-values: all parasites: 1634; mature parasites: 1177; immature parasites: 278.

Table S15: Estimated marginal means for the best model.

**A: All parasites**				
Estimates for fixed factors				
Factor	Level	Emmean/ trend (HPD range)	N	Unique taxa
**overall**	**overall**	**0.48 (0.057 - 0.94)**	**1634/207**	**113(80/82)**
**SE**	**overall**	**1.442 (1.093 - 1.8)**	**1634/207**	**113(80/82)**
parasite stage	immature	0.093 (-0.349 - 0.541)	278/49	30(25/27)
**parasite stage**	**mature**	**0.734 (0.289 - 1.167)**	**1177/196**	**110(79/79)**
**parasite stage**	**mix**	**0.757 (0.319 - 1.217)**	**104/11**	**11(10/10)**
parasite stage	switching	0.334 (-0.108 - 0.787)	75/16	10(8/9)
predator	absent	0.304 (-0.125 - 0.742)	1134/181	110(77/81)
**predator**	**dead end**	**0.481 (0.011 - 0.964)**	**79/20**	**13(10/10)**
**predator**	**present**	**0.651 (0.216 - 1.094)**	**421/78**	**44(36/31)**
**B: Mature parasites**				
Estimates for fixed factors				
Factor	Level	Emmean/ trend (HPD range)	N	Unique taxa
**overall**	**overall**	**0.779 (0.369 - 1.17)**	**1177/196**	**110(79/79)**
**SE**	**overall**	**2.011 (1.584 - 2.432)**	**1177/196**	**110(79/79)**
**predator**	**absent**	**0.628 (0.249 - 1.038)**	**865/170**	**107(76/78)**
**predator**	**dead end**	**0.748 (0.306 - 1.201)**	**70/18**	**12(9/9)**
**predator**	**present**	**0.959 (0.575 - 1.383)**	**242/73**	**44(36/31)**
**C: Immature parasites**				
Estimates for fixed factors				
Factor	Level	Emmean/ trend (HPD range)	N	Unique taxa
overall	overall	-0.257 (-0.926 - 0.384)	278/49	30(25/27)
**SE**	**overall**	**-0.883 (-1.652 - -0.088)**	**278/49**	**30(25/27)**
predator	absent	-0.273 (-0.897 - 0.381)	166/38	29(24/27)
predator	dead end	-0.476 (-1.285 - 0.295)	8/1	1(1/1)
predator	present	-0.022 (-0.673 - 0.616)	104/21	11(9/9)

N indicates the number of observations/ Number of independent studies. Unique taxa indicates the number of unique host-parasite-pairs (unique host taxa/unique parasite taxa). As far as possible, these estimates are based on species. However, in a limited number of cases, in which species level identification of parasites was not available, it is based on genus. Bold rows indicate estimates whose HPD (High posterior density) range does not overlap with 0, i.e. that estimates for these levels differ from 0.

References

Bernot, JP, Owen CL, Wolfe JM, Meland K, Olesen J, Crandall KA (2023). Major Revisions in Pancrustacean Phylogeny and Evidence of Sensitivity to Taxon Sampling, *Molecular Biology and Evolution* 40, msad175.

Chang WH, Lai AG (2018). Genome-wide analyses of the bHLH superfamily in crustaceans: reappraisal of higher-order groupings and evidence for lineage-specific duplications. *Royal Society Open Science* 5, 72433.

GIGA Community of Scientists (2014). The Global Invertebrate Genomics Alliance (GIGA): Developing Community Resources to Study Diverse Invertebrate Genomes. *Journal of Heredity* 105, 1–18.

Thomas, GWC, Dohmen, E, Hughes, DST *et al.* (2020). Gene content evolution in the arthropods. *Genome Biology* **21**, 15.
