# Supplementary material for: The Evolution and Ecology of Host Manipulation in Helminth Parasites: A Phylogenetic Meta‐Analysis
Source: Ecol Lett. 2026 Feb 18;29(2):e70340. doi: 10.1111/ele.70340 (PMC12916080; doi:10.1111/ele.70340)
Supplement: Supplementary file 5 — Table S1: Overview of host and parasite taxa included in the meta‐analysis for each parasite stage. N indicated number of observations/number of studies for each taxon. See Figure S1 for a phylogenetic tree of the species included in this meta‐analysis. [file ELE-29-0-s005.pdf]

Table S1: Overview of host and parasite taxa included in the meta analysis for each parasite stage. N indicated number of observations/ number of studies for each taxon. See Figure S1for a phylogenetic tree of the species included in this meta analysis.

Parasite group	Parasite	Host group	Host	N mature	N immature	N switching	N mix
Acanthocephalans	*Acanthocephalus anguillae*	Crustaceans	*Asellus aquaticus*	2/1 [1*]			
Acanthocephalans	*Acanthocephalus dirus*	Crustaceans	*Caecidotea intermedia*	10/5 [2*,3#,4*,5*,6]	1/1 [6]		1/1 [6]
Acanthocephalans	*Acanthocephalus galaxii*	Crustaceans	*Paracalliope fluviatilis*	1/1 [7#]			
Acanthocephalans	*Acanthocephalus lucii*	Crustaceans	*Asellus aquaticus*	23/7 [1*,8*,9*#,10,11*,12*,13*]			
Acanthocephalans	*Echinorhynchus borealis*	Crustaceans	*Pallasea quadrispinosa*	2/1 [14]			
Acanthocephalans	*Echinorhynchus truttae*	Crustaceans	*Gammarus pulex*	11/4 [15*,16,17*,18]			
Acanthocephalans	*Leptorhynchoides thecatus*	Crustaceans	*Hyalella azteca*	8/1 [19]			
Acanthocephalans	*Moniliformis moniliformis*	Insects	*Blattella germanica*	15/1 [20#]			
Acanthocephalans	*Moniliformis moniliformis*	Insects	*Diploptera punctata*	16/1 [21]			
Acanthocephalans	*Moniliformis moniliformis*	Insects	*Periplaneta americana*	22/4 [20#,22#,23*#,24#]			
Acanthocephalans	*Moniliformis moniliformis*	Insects	*Periplaneta australasiae*	2/1 [25#]			
Acanthocephalans	*Moniliformis moniliformis*	Insects	*Periplaneta brunnea*	16/1 [26#]			
Acanthocephalans	*Moniliformis moniliformis*	Insects	*Supella longipalpa*	14/1 [27]			
Acanthocephalans	*Neoechinorhynchus cylindratus*	Crustaceans	*Physocypria pustulosa*	1/1 [28]			
Acanthocephalans	*Octospiniferoides chandleri*	Crustaceans	*Cypridopsis vidua*	1/1 [28]			
Acanthocephalans	*Octospiniferoides chandleri*	Crustaceans	*Physocypria pustulosa*	1/1 [28]			
Acanthocephalans	*Oncicola venezuelensis*	Insects	*Nasutitermes acajutlae*	5/2 [29*,30*]			

Parasite group	Parasite	Host group	Host	N mature	N immature	N switching	N mix
Acanthocephalans	*Plagiorhynchus cylindraceus*	Crustaceans	*Armadillidium vulgare*	10/1 [31#]			
Acanthocephalans	*Polymorphus marilis*	Crustaceans	*Gammarus lacustris*	2/1 [32#]			
Acanthocephalans	*Polymorphus minutus*	Crustaceans	*Echinogammarus berilloni*	12/2 [33,34*]			
Acanthocephalans	*Polymorphus minutus*	Crustaceans	*Gammarus duebeni*	20/2 [12,35]			
Acanthocephalans	*Polymorphus minutus*	Crustaceans	*Gammarus fossarum*	6/2 [33,36]			
Acanthocephalans	*Polymorphus minutus*	Crustaceans	*Gammarus pulex*	41/13 [16,33,37*,38*,39,40*,41,42,43*,44*,45–47]	1/1 [37]*		
Acanthocephalans	*Polymorphus minutus*	Crustaceans	*Gammarus roeseli*	28/8 [38*,48*,49*,50,51,52*,53*,54]			
Acanthocephalans	*Polymorphus paradoxus*	Crustaceans	*Gammarus lacustris*	4/2 [32#,55]	2/1 [32][#]		
Acanthocephalans	*Pomphorhynchus laevis*	Crustaceans	*Echinogammarus stammeri*	10/3 [56*,57*,58]			
Acanthocephalans	*Pomphorhynchus laevis*	Crustaceans	*Gammarus fossarum*	13/2 [59,60*]			
Acanthocephalans	*Pomphorhynchus laevis*	Crustaceans	*Gammarus pulex*	94/26 [39,40*,41,42,43*,45,60*,61*,62*,63,64*#,65*,66, 67*#,68–70,71*,72*,73*,74,75*,76–78,79*]	14/3 [65*,72*,80]		4/1 [66]
Acanthocephalans	*Pomphorhynchus laevis*	Crustaceans	*Gammarus roeseli*	7/3 [63,76,81*]			
Acanthocephalans	*Pomphorhynchus tereticollis*	Crustaceans	*Gammarus fossarum*	26/3 [36,82*,83]	6/1 [83]		8/1 [83]
Acanthocephalans	*Pomphorhynchus tereticollis*	Crustaceans	*Gammarus pulex*	15/6 [40*,42,45,78,84,85*]			
Acanthocephalans	*Profilicollis altmani*	Crustaceans	*Emerita analoga*	1/1 [86]			
Acanthocephalans	*Profilicollis antarcticus*	Crustaceans	*Hemigrapsus crenulatus*	3/1 [87]			

Parasite group	Parasite	Host group	Host	N mature	N immature	N switching	N mix
Acanthocephalans	*Pseudocorynosoma* sp.	Crustaceans	*Hyalella patagonica*	1/1 [88]			
Acanthocephalans	*Pseudocorynosoma constrictum*	Crustaceans	*Hyalella azteca*	5/1 [89]			
Acanthocephalans	*Sphaerechinorhynchus*	Reptiles	*Eulamprus quoyii*	3/1 [90#]			
Cestods	*Anomotaenia brevis*	Insects	*Temnothorax nylanderi*	4/1 [91]			
Cestods	*Anomotaenia microphallos*	Crustaceans	*Artemia parthenogenetica*	1/1 [92]			
Cestods	*Anomotaenia tringae*	Crustaceans	*Artemia parthenogenetica*	2/2 [92,93]			
Cestods	*Confluaria podicipina*	Crustaceans	*Artemia parthenogenetica*	1/1 [92]			
Cestods	*Cyathocephalus truncatus*	Crustaceans	*Gammarus pulex*	3/2 [94#,95]			
Cestods	*Diphyllobothrium* sp.	Crustaceans	*Cyclops strenuus*	2/1 [96#]			
Cestods	*Eubothrium salvelini*	Crustaceans	*Cyclops vernalis*	3/1 [97]	6/1 [97]	3/1 [97]	
Cestods	*Eurycestus avoceti*	Crustaceans	*Artemia parthenogenetica*	2/2 [92,93]			
Cestods	*Flamingolepis liguloides*	Crustaceans	*Artemia parthenogenetica*	10/3 [93,98,99]			
Cestods	*Flamingolepis flamingo*	Crustaceans	*Artemia parthenogenetica*	1/1 [92]			
Cestods	*Hymenolepis diminuta*	Insects	*Tenebrio molitor*	21/3 [100,101,102#]	12/1 [100]	4/1 [100]	
Cestods	*Hymenolepis diminuta*	Insects	*Tribolium castaneum*	12/2[103,104]			
Cestods	*Hymenolepis diminuta*	Insects	*Tribolium confusum*	5/2 [105#]	1/1 [105#]		
Cestods	*Lateriporus mathevossianae*	Crustaceans	*Gammarus lacustris*	2/1 [32#]			
Cestods	*Ligula intestinalis*	Fish	*Engraulicypris sardella*	3/1 [106]	3/1 [106]		
Cestods	*Ligula intestinalis*	Fish	*Phoxinus phoxinus*	2/1 [107]			

Parasite group	Parasite	Host group	Host	N mature	N immature	N switching	N mix
Cestods	*Ligula intestinalis*	Fish	*Rutilus rutilus*	10/2 [108,109]			
Cestods	*Schistocephalus solidus*	Crustaceans	*Cyclops abyssorum*	2/1 [110]	2/1 [110]		
Cestods	*Schistocephalus solidus*	Crustaceans	*Cyclops scutifer*	1/1 [110]			
Cestods	*Schistocephalus solidus*	Crustaceans	*Macrocyclops albidus*	111/10 [111,112#,113–117,118#,119,120]	67/9 [111–114,116,120,121]	26/5 [111]	44/1 [111,113,115,116,119]
Cestods	*Schistocephalus solidus*	Fish	*Gasterosteus aculeatus*	89/15 [122–125,126#,127–136]	44/4 [128,135,136#,137]	16/2 [135,138]	8/1 [135]
Cestods	*Taenia crassiceps*	Mammals	*Mus musculus*	10/2 [139,140]			
Cestods	*Taenia pisiformis*	Mammals	*Oryctolagus cuniculus*	9/1 [141]	9/1 [141]	6/1 [141]	
Cestods	*Triaenophorus crassus*	Crustaceans	*Cyclops strenuus*	21/1 [142#]	5/1 [142#]		
Nematods	*Anisakis pegreffii*	Fish	*Dicentrarchus labrax*	19/1 [143]			
Nematods	*Camallanus lacustris*	Crustaceans	*Macrocyclops albidus*	30/1 [119]	22/2 [119,121]	6/1 [119]	21/1 [119]
Nematods	*Dispharynx nasuta*	Crustaceans	*Armadillidium vulgare*	5/1 [144#]			
Nematods	*Hedruris suttonae*	Crustaceans	*Hyalella patagonica*				2/1 [88]
Nematods	*Myrmeconema neotropicum*	Insects	*Cephalotes atratus*	7/2 [145,146]			
Nematods	*Parelaphostrongylus tenuis*	Molluscs	*Appalachina sayanus*	12/1 [147]	14/1 [147]	4/1 [147]	
Nematods	*Protospirura muricola*	Insects	*Tribolium confusum*	17/1 [148]	2/1 [148]		
Nematods	*Pterygodermatites peromysci*	Insects	*Ceuthophilus pallidipes*	4/1 [149#]			
Nematods	*Skrjabinoclava morrisoni*	Crustaceans	*Corophium volutator*	8/1 [150#]	2/1 [150]		

Parasite group	Parasite	Host group	Host	N mature	N immature	N switching	N mix
Nematods	*Toxocara canis*	Mammals	*Mus musculus*	52/3 [151#,152,153#]			
Nematods	*Toxocara canis*	Mammals	*Rattus norvegicus*	2/1 [154]			
Nematods	*Toxocara cati*	Mammals	*Mus musculus*	30/1 [152]			
Trematods	*Acanthocollaritrema umbilicatum*	Fish	*Poecilia vivipara*	20/1 [155#]			
Trematods	*Allocreadium crassum*	Molluscs	*Pisidium amnicum*	1/1 [156]	1/1 [156]		
Trematods	*Apatemon* sp.	Fish	*Nothobranchius furzeri*	2/1 [157]			
Trematods	*Ascocotyle pindoramensis*	Fish	*Poecilia vivipara*		5/1 [158]		
Trematods	*Atriophallophorus winterbourni*	Molluscs	*Potamopyrgus antipodarum*	1/1 [159]	1/1 [159]		
Trematods	*Australapatemon* sp.	Annelids	*Erpobdella octoculata*	3/1 [160]			2/1 [160]
Trematods	*Coitocaecum parvum*	Crustaceans	*Paracalliope fluviatilis*	7/3 [7#,161,162]	2/1 [161]		
Trematods	*Crassiphiala bulboglossa*	Fish	*Fundulus diaphanus*	4/1 [163#]			
Trematods	*Curtuteria australis*	Molluscs	*Austrovenus stutchburyi*	1/1 [164]			
Trematods	*Cymatocarpus solearis*	Crustaceans	*Panulirus argus*	5/1 [165]			
Trematods	*Derogenes varicus*	Chaetognaths	*Flaccisagitta enflata*	1/1 [166]			
Trematods	*Derogenes varicus*	Chaetognaths	*Parasagitta friderici*	2/1 [166]			
Trematods	*Diplostomum pseudospathaceum*	Fish	*Oncorhynchus mykiss*	10/3 [167–169]	10/4 [167–170]	4/1 [169]	1/1 [167]
Trematods	*Diplostomum pseudospathaceum*	Fish	*Salvelinus malma*	5/1 [171]			
Trematods	*Diplostomum spathaceum*	Fish	*Oncorhynchus mykiss*	20/6 [172,173#,174–177]	4/2 [172,174]	3/2 [172,174]	
Trematods	*Euhaplorchis californiensis*	Fish	*Fundulus parvipinnis*	1/1 [178]			12/1 [179]

Parasite group	Parasite	Host group	Host	N mature	N immature	N switching	N mix
Trematods	*Gynaecotyla adunca*	Crustaceans	*Corophium volutator*	2/1 [180]			
Trematods	*Hemiurus luehei*	Chaetognaths	*Flaccisagitta enflata*	2/1 [166]			
Trematods	*Hemiurus luehei*	Chaetognaths	*Mesosagitta minima*	1/1 [166]			
Trematods	*Hemiurus luehei*	Chaetognaths	*Parasagitta friderici*	2/1 [166]			
Trematods	*Leucochloridium paradoxum*	Molluscs	*Succinea putris*	5/1 [181]			
Trematods	*Levinseniella byrdi*	Crustaceans	*Orchestia grillus*	4/1 [182]			
Trematods	*Maritrema novaezealandensis*	Crustaceans	*Paracalliope novizealandiae*	2/1 [183]	2/1 [183]		
Trematods	*Maritrema oocysta*	Molluscs	*Peringia ulvae*	1/1 [184]			
Trematods	*Microphallus* sp.	Crustaceans	*Austridotea annectens*	5/1 [185]			
Trematods	*Microphallus* sp.	Crustaceans	*Faxonius propinquus*	3/1 [186]			
Trematods	*Microphallus* sp.	Crustaceans	*Faxonius rusticus*	8/2 [186,187]			
Trematods	*Microphallus* sp.	Crustaceans	*Faxonius virilis*	2/1 [186]			
Trematods	*Microphallus* sp.	Crustaceans	*Paracalliope fluviatilis*	5/2 [7#,162#]			
Trematods	*Microphallus* sp.	Molluscs	*Potamopyrgus antipodarum*	13/3 [188,189,190#]	2/1 [189]		
Trematods	*Microphallus papillorobustus*	Crustaceans	*Gammarus insensibilis*	7/2 [191,192]			
Trematods	*Microphallus piriformes*	Molluscs	*Littorina saxatilis*	19/1 [193]			
Trematods	*Microphallus similis*	Crustaceans	*Carcinus maenas*	1/1 [194]	10/2 [194,195]		
Trematods	*Microphallus turgidus*	Crustaceans	*Palaemonetes pugio*	18/2 [196,197]	8/1 [198]		
Trematods	*Ornithodiplostomum ptychocheilus*	Fish	*Pimephales promelas*	7/3 [199,200,201#]	15/3 [199,201#,202#]	3/2 [199,201#]	1/1 [199]

Parasite group	Parasite	Host group	Host	N mature	N immature	N switching	N mix
Trematods	*Parvatrema affinis*	Molluscs	*Macoma balthica*	1/1 [203]			
Trematods	*Ribeiroia ondatrae*	Amphibians	*Anaxyrus boreas*		5/1 [204]		
Trematods	*Stegodexamene anguillae*	Fish	*Gobiomorphus cotidianus*	6/1 [205]			
Trematods	*Telogaster opisthorchis*	Fish	*Gobiomorphus cotidianus*	3/1 [205]			
Trematods	*Tylodelphys* sp.	Fish	*Galaxias maculatus*	4/1 [206]			
Trematods	*Tylodelphys clavata*	Fish	*Perca fluviatilis*	9/1 [207]			

*data (partly) obtained from: Fayard, Marion, François Xavier Dechaume-Moncharmont, Rémi Wattier, und Marie Jeanne Perrot-Minnot. 2020. „Magnitude and direction of parasite-induced phenotypic alterations: a meta-analysis in acanthocephalans“. *Biological Reviews* 95 (5): 1233–51. https://doi.org/10.1111/brv.12606.
#data (partly) obtained from: Nakagawa, Shinichi, Robert Poulin, Kerrie Mengersen, u. a. 2015. „Meta-analysis of variation: Ecological and evolutionary applications and beyond“. *Methods in Ecology and Evolution* 6 (2): 143–52. https://doi.org/10.1111/2041-210X.12309.

References

1. Lyndon AR. The role of acanthocephalan parasites in the predation of freshwater isopods by fish. Aquatic Predators and their Prey (Eds SPR Greenstreet and M L Tasker). Oxford; 1996. pp. 26–32.

2. Camp JW, Huizinga HW. Altered color, behavior and predation susceptibility of the isopod *Asellus intermedius* infected with *Acanthocephalus dirus*. J Parasitol. 1979;65: 667–669. doi:10.2307/3280340

3. Hechtel LJ, Johnson CL, Juliano SA. Modification of antipredator behavior of *Coecidotea intermedius* by its parasite *Acanthocephalus dirus*. Ecology. 1993;74: 710–713.

4. Kopp DA, Bierbower SM, Murphy AD, Mormann K, Sparkes TC. Parasite-related modification of mating behaviour and refuge use in the aquatic isopod *Caecidotea intermedius*: neurological correlates. Behaviour. 2016;153: 947–961. doi:10.1163/1568539X-00003379

5. Park T, Sparkes TC. Multidimensionality of Modification in an Isopod-Acanthocephalan System. Front Ecol Evol. 2017;5: 103. doi:10.3389/fevo.2017.00103

6. Sparkes TC, Wright VM, Renwick DT, Weil KA, Talkington JA, Milhalyov M. Intra-specific host sharing in the manipulative parasite *Acanthocephalus dirus*: does conflict occur over host modification? Parasitology. 2004;129: 335–340. doi:10.1017/S0031182004005645

7. Rauque CA, Paterson RA, Poulin R, Tompkins DM. Do different parasite species interact in their effects on host fitness? A case study on parasites of the amphipod *Paracalliope fluviatilis*. Parasitology. 2011;138: 1176–1182. doi:10.1017/S0031182011000928

8. Brattey J. The effects of larval *Acanthocephalus lucii* on the pigmentation, reproduction, and susceptibility to predation of the isopod *Asellus aquaticus*. J Parasitol. 1983;69: 1172–1173.

9. Benesh DP, Valtonen ET, Seppälä O. Multidimensionality and intra-individual variation in host manipulation by an acanthocephalan. Parasitology. 2008;135: 617–626. doi:10.1017/S0031182008004216

10. Benesh DP, Hasu T, Seppälä O, Valtonen ET. Seasonal changes in host phenotype manipulation by an acanthocephalan: time to be transmitted? Parasitology. 2009;136: 219–230. doi:10.1017/S0031182008005271

11. Benesh DP, Seppälä O, Valtonen ET. Acanthocephalan size and sex affect the modification of intermediate host colouration. Parasitology. 2009;136: 847–854. doi:10.1017/S0031182009006180

12. MacNeil C, Dick JTA, Hatcher MJ, Dunn AM. Differential drift and parasitism in invading and native *Gammarus* spp. (Crustacea: Amphipoda). Ecography. 2003;26: 467–473. doi:10.1034/j.1600-0587.2003.03460.x

13. Seppälä O, Valtonen ET, Benesh DP. Host manipulation by parasites in the world of dead-end predators: adaptation to enhance transmission? Proceeding R Soc B. 2008;275: 1611–1615. doi:10.1098/rspb.2008.0152

14. Benesh DP, Kitchen J, Pulkkinen K, Hakala I, Valtonen ET. The Effect of *Echinorhynchus borealis* (Acanthocephala) Infection on the Anti-Predator Behavior of a Benthic Amphipod The Effect of *Echinorhynchus borealis* (Acanthocephala) Infection on the Anti-Predator Behavior of a Benthic Amphipod. 2008;94: 542–545.

15. MacNeil C, Fielding NJ, Hume KD, Dick JTA, Elwood RW, Hatcher MJ, et al. Parasite altered micro-distribution of *Gammarus pulex* (Crustacea: Amphipoda). Int J Parasitol. 2003;33: 57–64. doi:10.1016/S0020-7519(02)00229-1

16. Lagrue C, Güvenatam A, Bollache L. Manipulative parasites may not alter intermediate host distribution but still enhance their transmission: field evidence for increased vulnerability to definitive hosts and non-host predator avoidance. Parasitology. 2013;140: 258–65. doi:10.1017/S0031182012001552

17. Laverty C, Brenner D, McIlwaine C, Lennon JJ, Dick JTA, Lucy FE, et al. Temperature rise and parasitic infection interact to increase the impact of an invasive species. Int J Parasitol. 2017;47: 291–296. doi:10.1016/j.ijpara.2016.12.004

18. Fielding NJ, MacNeil C, Dick JTA, Elwood RW, Riddell GE, Dunn AM. Effects of the acanthocephalan parasite *Echinorhynchus truttae* on the feeding ecology of *Gammarus pulex* (Crustacea: Amphipoda). J Zool. 2003;261: 321–325. doi:10.1017/S0952836903004230

19. Stone CF, Moore J. Parasite-induced alteration of odour responses in an amphipod-acanthocephalan system. Int J Parasitol. 2014;44: 969–975. doi:10.1016/j.ijpara.2014.06.012

20. Gotelli NJ, Moore J. Altered host behaviour in a cockroach-acanthocephalan association. Anim Behav. 1992;43: 949–959.

21. Allely Z, Moore J, Gotelli NJ. *Moniliformis moniliformis* infection has no effect on some behaviors of the cockroach. Source J Parasitol. 1992;78: 524–526.

22. Moore J. Altered Behavior in Cockroaches (*Periplaneta americana*) Infected with an Archiacanthocephalan, *Moniliformis moniliformis*. J Parasitol. 1983;69: 1174–1176.

23. Wilson K, Edwards J. The effects of parasitic infection on the behaviour of an intermediate host, the American Cockroach, *Periplaneta americana*, infected with the Acanthocephalan, *Moniliformis moniliformis*. Anim Behav. 1986;34: 942–944. doi:10.1016/s0003-3472(86)80088-4

24. Libersat F, Moore J. The parasite *Moniliformis moniliformis* alters the escape response of its cockroach host *Periplaneta americana*. J Insect Behav. 2000;13: 103–110. doi:10.1023/a:1007719710664

25. Moore J, Freehling M, Gotelli NJ. Altered Behavior in Two Species of Blattid Cockroaches Infected with *Moniliformis moniliformis* (Acanthocephala). J Parasitol. 1994;80: 220–223.

26. Carmichael LM, Moore J. A comparison of behavioral alterations in the brown cockroach, *Periplaneta brunnea*, and the American cockroach, *Periplaneta americana*, infected with the acanthocephalan, *Moniliformis moniliformis*. J Parasitol. 1991;77: 931–936.

27. Moore J, Gotelli NJ. *Moniliformis moniliformis* Increases Cryptic Behaviors in the Cockroach *Supella longipalpa*. J Parasitol. 1992;78: 49. doi:10.2307/3283684

28. DeMont DJ, Corkum KC. The life cycle of *Octospiniferoides chandleri* Bullock , 1957 (Acanthocephala : Neoechinorhynchidae) with some observations on parasite-induced, photophilic behavior in ostracods. J Parasitol. 1982;68: 125–130.

29. Fuller CA, Rock P, Philips T. Behavior, color changes, and predation risk Induced by Acanthocephalan parasitism in the Caribbean termite *Nasutitermes acajutlae*. Caribb J Sci. 2003;39: 128–135.

30. Fuller CA, Jeyasingh PD. Acanthocephalan (Oligacanthorhynchidae) parasitism of the Caribbean termite *Nasutitermes acajutlae*: implications for reproductive success. Insectes Sociaux. 2004;51. doi:10.1007/s00040-003-0727-8

31. Moore J. Responses of an avian predator and its isopod prey to an Acanthocephalan parasite. Ecology. 1983;64: 1000–1015.

32. Bethel WM, Holmes JC. Altered evasive behavior and responses to light in amphipods harboring acanthocephalan cystacanths. J Parasitol. 1973;59: 945–956. doi:10.2307/3278623

33. Farahani S, Palsboll PJ, Pen I, Komdeur J. Effects of parasites upon non-host predator avoidance behaviour in native and invasive gammarids. Parasitology. 2021;148: 354–360. doi:10.1017/S0031182020002140

34. Jacquin L, Mori Q, Pause M, Steffen M, Medoc V. Non-specific manipulation of gammarid behaviour by *P. minutus* parasite enhances their predation by definitive bird hosts. PLoS One. 2014;9: e101684–e101684. doi:10.1371/journal.pone.0101684

35. Williams MA, Holland C V, Donohue I. Warming can alter host behavior in a similar manner to infection with behavior-manipulating parasites. Oecologia. 2020;194: 65–74. doi:10.1007/s00442-020-04745-2

36. Labaude S, Cézilly F, Rigaud T. Temperature-related intraspecific variability in the behavioral manipulation of acanthocephalan parasites on their gammarid hosts. Biol Bull. 2017;232: 82–90. doi:10.1086/692684

37. Bailly Y, Cezilly F, Rigaud T. Stage-dependent behavioural changes but early castration induced by the acanthocephalan parasite *Polymorphus minutus* in its *Gammarus pulex* intermediate host. Parasitology. 2018;145: 260–268. doi:10.1017/S0031182017001457

38. Bauer A, Haine ER, Perrot-Minnot MJ, Rigaud T. The acanthocephalan parasite *Polymorphus minutus* alters the geotactic and clinging behaviours of two sympatric amphipod hosts: the native *Gammarus pulex* and the invasive *Gammarus roeseli*. J Zool. 2005;267: 39–43. doi:10.1017/S0952836905007223

39. Cézilly F, Grégoire A, Bertin A. Conflict between co-occurring manipulative parasites? An experimental study of the joint influence of two acanthocephalan parasites on the behaviour of *Gammarus pulex*. Parasitology. 2000;120: 625–630. doi:10.1017/S0031182099005910

40. Cornet S, Franceschi N, Bauer A, Rigaud T, Moret Y. Immune depression induced by acanthocephalan parasites in their intermediate crustacean host: consequences for the risk of super-infection and links with host behavioural manipulation. Int J Parasitol. 2009;39: 221–9. doi:10.1016/j.ijpara.2008.06.007

41. Kaldonski N, Perrot-Minnot M-J, Dodet RRR, Martinaud G, Cézilly F. Carotenoid-based colour of acanthocephalan cystacanths plays no role in host manipulation. Proceeding R Soc B. 2009;276: 169–176. doi:10.1098/rspb.2008.0798

42. Kaldonski N, Perrot-Minnot M-J, Motreuil S, Cézilly F. Infection with acanthocephalans increases the vulnerability of *Gammarus pulex* (Crustacea, Arnphipoda) to non-host invertebrate predators. Parasitology. 2008;135: 627–632. doi:10.1017/S003118200800423X

43. Kaldonski N, Perrot-Minnot M-J, Cézilly F. Differential influence of two acanthocephalan parasites on the antipredator behaviour of their common intermediate host. Anim Behav. 2007;74: 1311–1317. doi:10.1016/j.anbehav.2007.02.027

44. Marriott DR, Collins ML, Paris RM, Gudgin DR, Barnard CJ, McGregor PK, et al. Behavioural modifications and increased predation risk of *Gammarus pulex* infected with *Polymorphus minutus*. J Biol Educ. 1989;23: 135–141. doi:10.1080/00219266.1989.9655047

45. Tain L, Perrot-Minnot M-J, Cézilly F. Altered host behaviour and brain serotonergic activity caused by acanthocephalans: evidence for specificity. Proc R Soc B. 2006;273: 3039–3045. doi:10.1098/rspb.2006.3618

46. Thuenken T, Vitt S, Baldauf SA, Jung T, Frommen JG. Individual behavioural responses of an intermediate host to a manipulative acanthocephalan parasite and the effects of intra-specific parasite competition. Evol Ecol Res. 2018;19: 503–516.

47. Thünken T, Baldauf SA, Bersau N, Bakker TCM, Kullmann H, Frommen JG. Impact of olfactory non-host predator cues on aggregation behaviour and activity in *Polymorphus minutus* infected *Gammarus pulex*. Hydrobiologia. 2010;654: 137–145. doi:10.1007/s10750-010-0377-6

48. Medoc V, Beisel J-N. An acanthocephalan parasite boosts the escape performance of its intermediate host facing non-host predators. Parasitology. 2008;135: 977–984. doi:10.1017/S0031182008004447

49. Medoc V, Beisel J-N. Field evidence for non-host predator avoidance in a manipulated amphipod. Naturwissenschaften. 2009;96: 513–523. doi:10.1007/s00114-008-0503-8

50. Medoc V, Bollache L, Beisel J-N. Host manipulation of a freshwater crustacean (*Gammarus roeseli*) by an acanthocephalan parasite (*Polymorphus minutus*) in a biological invasion context. Int J Parasitol. 2006;36: 1351–1358. doi:10.1016/j.ijpara.2006.07.001

51. Médoc V, Rigaud T, Bollache L, Beisel J-N. A manipulative parasite increasing an antipredator response decreases its vulnerability to a nonhost predator. Anim Behav. 2009;77: 1235–1241. doi:10.1016/j.anbehav.2009.01.029

52. Perrot-Minnot M-J, Maddaleno M, Cézilly F. Parasite-induced inversion of geotaxis in a freshwater amphipod: a role for anaerobic metabolism? Funct Ecol. 2016;30: 780–788. doi:10.1111/1365-2435.12516

53. Piscart C, Webb D, Beisel JN. An acanthocephalan parasite increases the salinity tolerance of the freshwater amphipod *Gammarus roeseli* (Crustacea: Gammaridae). Naturwissenschaften. 2007;94: 741–747. doi:10.1007/s00114-007-0252-0

54. Médoc V, Piscart C, Maazouzi C, Simon L, Beisel JN. Parasite-induced changes in the diet of a freshwater amphipod: Field and laboratory evidence. Parasitology. 2011;138: 537–546. doi:10.1017/S0031182010001617

55. Bethel WM, Holmes JC. Increased vulnerability of amphipods to predation owing to altered behavior induced by larval acanthocephalans. Can J Zool. 1977;55: 110–115. doi:10.1139/z77-013

56. Dezfuli BS, Maynard BJ, Wellnitz TA. Activity levels and predator detection by amphipods infected with an acanthocephalan parasite, *Pomphorhynchus laevis*. Folia Parasitol (Praha). 2003;50: 129–134. doi:10.14411/fp.2003.023

57. Maynard BJ, Wellnitz TA, Zanini N, Wright WG, Dezfuli BS. Parasite-altered behavior in a crustacean intermediate host: Field and laboratory studies. J Parasitol. 1998;84: 1102–1106. doi:10.2307/3284656

58. Wellnitz T, Giari L, Maynard B, Dezfuli BS. A parasite spatially structures its host population. Oikos. 2003;100: 263–268. doi:10.1034/j.1600-0706.2003.12153.x

59. Fayard M, Cezilly F, Perrot-Minnot M-J. Inter-population variation in the intensity of host manipulation by the fish acanthocephalan *Pomphorhynchus tereticollis*: are differences driven by predation risk? Parasitology. 2019;146: 1296–1304. doi:10.1017/S0031182019000520

60. Perrot-Minnot M-J, Sanchez-Thirion K, Cézilly F. Multidimensionality in host manipulation mimicked by serotonin injection. Proceeding R Soc B. 2014;281: 20141915–20141915. doi:10.1098/rspb.2014.1915

61. Bakker TCM, Mazzi D, Zala S. Parasite-induced changes in behavior and color make *Gammarus pulex* more prone to fish predation. Ecology. 1997;78: 1098–1104. doi:10.1890/0012-9658(1997)078{[}1098:PICIBA]2.0.CO;2

62. Baldauf SA, Thünken T, Frommen JG, Bakker TCM, Heupel O, Kullmann H. Infection with an acanthocephalan manipulates an amphipod's reaction to a fish predator's odours. Int J Parasitol. 2007;37: 61–65. doi:10.1016/j.ijpara.2006.09.003

63. Bauer A, Trouvé S, Grégoire A, Bollache L, Cézilly F. Differential influence of *Pomphorhynchus laevis* (Acanthocephala) on the behaviour of native and invader gammarid species. Int J Parasitol. 2000;30: 1453–1457. doi:10.1016/S0020-7519(00)00138-7

64. Brown AF, Thompson DBA. Parasite manipulation of host behaviour: acanthocephalans and shrimps in the laboratory. J Biol Educ. 1986;20: 121–127. doi:10.1080/00219266.1986.9654798

65. Dianne L, Perrot-Minnot M-J, Bauer A, Gaillard M, Léger E, Rigaud T. Protection first then facilitation: A manipulative parasite modulates the vulnerability to predation of its intermediate host according to its own developmental stage. Evolution. 2011;65: 2692–2698. doi:10.1111/j.1558-5646.2011.01330.x

66. Dianne L, Rigaud T, Leger E, Motreuil S, Bauer A, Perrot-Minnot M-J. Intraspecific conflict over host manipulation between different larval stages of an acanthocephalan parasite. J Evol Biol. 2010;23: 2648–2655. doi:10.1111/j.1420-9101.2010.02137.x

67. Durieux R, Rigaud T, Médoc V. Parasite-induced suppression of aggregation under predation risk in a freshwater amphipod: Sociality of infected amphipods. Behav Processes. 2012;91: 207–213. doi:10.1016/j.beproc.2012.08.002

68. Fanton H, Franquet E, Logez M, Kaldonski N. Effects of temperature and a manipulative parasite on the swimming behaviour of *Gammarus pulex* in flowing water. Hydrobiologia. 2021;848: 4467–4476. doi:10.1007/s10750-021-04655-1

69. Fanton H, Franquet E, Logez M, Kaldonski N. *Pomphorhynchus laevis* manipulates *Gammarus pulex* behaviour despite salt pollution. Freshw Biol. 2020;65: 1718–1725. doi:10.1111/fwb.13573

70. Franceschi N, Bollache L, Cornet S, Bauer A, Motreuil S, Rigaud T. Co-variation between the intensity of behavioural manipulation and parasite development time in an acanthocephalan–amphipod system. J Evol Biol. 2010;23: 2143–2150. doi:10.1111/j.1420-9101.2010.02076.x

71. Franceschi N, Cornet S, Bollache L, Dechaume-Moncharmont FX, Bauer A, Motreuil S, et al. Variation between populations and local adaptation in acanthocephalan-induced parasite manipulation. Evolution. 2010;64: 2417–2430. doi:10.1111/j.1558-5646.2010.01006.x

72. Franceschi N, Bauer A, Bollache L, Rigaud T. The effects of parasite age and intensity on variability in acanthocephalan-induced behavioural manipulation. Int J Parasitol. 2008;38: 1161–1170. doi:10.1016/j.ijpara.2008.01.003

73. Kennedy CR, Broughton PF, Hine PM. The status of brown and rainbow trout, *Salmo trutta* and *S. gairdneri* as hosts of the acanthocephalan, *Pomphorhynchus laevis*. J Fish Biol. 1978;13: 265–275. doi:10.1111/j.1095-8649.1978.tb03434.x

74. Labaude S, Cezilly F, De Marco L, Rigaud T. Increased temperature has no consequence for behavioral manipulation despite effects on both partners in the interaction between a crustacean host and a manipulative parasite. Sci Rep. 2020;10: 11670. doi:10.1038/s41598-020-68577-z

75. Labaude S, Cézilly F, Tercier X, Rigaud T. Influence of host nutritional condition on post-infection traits in the association between the manipulative acanthocephalan *Pomphorhynchus laevis* and the amphipod *Gammarus pulex*. Parasit Vectors. 2015;8: 403–403. doi:10.1186/s13071-015-1017-9

76. Lagrue C, Kaldonski N, Perrot-Minnot M-J, Motreuil S, Bollache LL. Modification of hosts' behavior by a parasite: Field evidence for adaptive manipulation. Ecology. 2007;88: 2839–2847.

77. McCahon CP, Maund SJ, Poulton MJ. The effect of the acanthocephalan parasite *Pomphorhynchus laevis* on the drift of its intermediate host *Gammarus pulex*. Freshw Biol. 1991;25: 507–513. doi:10.1111/j.1365-2427.1991.tb01393.x

78. Perrot-Minnot MJ. Larval morphology, genetic divergence, and contrasting levels of host manipulation between forms of *Pomphorhynchus laevis* (Acanthocephala). Int J Parasitol. 2004;34: 45–54. doi:10.1016/j.ijpara.2003.10.005

79. Tain L, Perrot-Minnot M-J, Cézilly F. Differential influence of *Pomphorhynchus laevis* (Acanthocephala) on brain serotonergic activity in two congeneric host species. Biol Lett. 2007;3: 68–71. doi:10.1098/rsbl.2006.0583

80. Dianne L, Perrot-Minnot M-J, Bauer A, Guvenatam A, Rigaud T. Parasite-induced alteration of plastic response to predation threat: increased refuge use but lower food intake in *Gammarus pulex* infected with the acanothocephalan *Pomphorhynchus laevis*. Int J Parasitol. 2014;44: 211–216. doi:10.1016/j.ijpara.2013.11.001

81. Moret Y, Bollache L, Wattier R, Rigaud T. Is the host or the parasite the most locally adapted in an amphipod-acanthocephalan relationship? A case study in a biological invasion context. Int J Parasitol. 2007;37: 637–644. doi:10.1016/j.ijpara.2006.12.006

82. Labaude S, Rigaud T, Cézilly F. Additive effects of temperature and infection with an acanthocephalan parasite on the shredding activity of *Gammarus fossarum* (Crustacea: Amphipoda): the importance of aggregative behavior. Glob Change Biol. 2017;23: 1415–1424. doi:10.1111/gcb.13490

83. Cozzarolo C-S, Perrot-Minnot M-J. Infection with an acanthocephalan helminth reduces anxiety-like behaviour in crustacean host. Sci Rep. 2022;12: 21649. doi:10.1038/s41598-022-25484-9

84. Perrot-Minnot M-J, Maddaleno M, Balourdet A, Cézilly F. Host manipulation revisited: no evidence for a causal link between altered photophobia and increased trophic transmission of amphipods infected with acanthocephalans. Funct Ecol. 2012;26: 1007–1014. doi:10.1111/j.1365-2435.2012.02027.x

85. Perrot-Minnot M-J, Kaldonski N, Cezilly F. Increased susceptibility to predation and altered anti-predator behaviour in an acanthocephalan-infected amphipod. Int J Parasitol. 2007;37: 645–651. doi:10.1016/j.ijpara.2006.12.005

86. Figueroa LB, Urbina MA, Riedemann A, Rodriguez SM, Paschke K. Decreased metabolic rate in the mole crabs *Emerita analoga*, infected with the Acanthocephalan *Profilicollis altmani*. J Parasitol. 2019;105: 19–24. doi:10.1645/18-29

87. Pulgar, Jose, Ardana, Marcela, Vergara, Elisio, George-Nascimento, Mario. Behavior of the esturian crab *Hemigrapsus crenulatus* (Milne-Edwards 1837) in relation to the parasitism by the acanthocephalan *Profilicollis anarticus* (Zdzitowiechi 1985) in southern Chile. Revisita Chil Hist Nat. 1995;68: 439–450.

88. Casalins L, Brugni N, Rauque C, Casalins LM, Brugni NL, Rauque CA. The behavior response of amphipods infected by *Hedruris suttonae* (Nematoda) and *Pseudocorynosoma* Sp. (Acanthocephala). J Parasitol. 2015;101: 647–650. doi:10.1645/13-327

89. Benesh DP, Duclos LM, Nickol BB. The behavioral response of amphipods harboring Corynosoma constrictum (Acanthocephala) to various components of light. J Parasitol. 2005;91: 731–736. doi:10.1645/GE-440R.1

90. Daniels CB. The effect of infection by a parasitic worm on swimming and diving in the water skink, *Sphenomorphus quoyii*. J Herpetol. 1985;19: 160. doi:10.2307/1564431

91. Trabalon M, Plateaux L, Péru L, Ve Bagnères C A-G, Hartmann N. Modification of morphological characters and cuticular compounds in worker ants *Leptothorax nylanderi* induced by endoparasites *Anomotaenia brevis*. J Insect Physiol. 2000;46: 169–178.

92. Sanchez MI, Georgiev BB, Green AJ. Avian cestodes affect the behaviour of their intermediate host *Artemia parthenogenetica*: An experimental study. Behav Processes. 2007;74: 293–299. doi:10.1016/j.beproc.2006.11.002

93. Sánchez MI, Georgiev BB, Nikolov PN, Vasileva GP, Green AJ. Red and transparent brine shrimps (*Artemia parthenogenetica*): A comparative study of their cestode infections. Parasitol Res. 2006;100: 111–114. doi:10.1007/s00436-006-0248-2

94. Franceschi N, Rigaud T, Moret Y, Hervant F, Bollache L. Behavioural and physiological effects of the trophically transmitted cestode parasite, *Cyathocephalus truncatus*, on its intermediate host, *Gammarus pulex*. Parasitology. 2007;134: 1839–1847. doi:10.1017/S0031182007003228

95. Bollache L. Effects of the cestode parasite, *Cyathocephalus truncatus*, on the fecundity and feeding rate of *Gammarus pulex* (Crustacea: Amphipoda). Parasitol Res. 2016;115: 445–447. doi:10.1007/s00436-015-4810-7

96. Pasternak AF, Huntingford FA, Crompton DWT. Changes in metabolism and behaviour of the freshwater copepod *Cyclops strenuus abyssorum* infected with *Diphyllobothrium* spp. Parasitology. 1995;110: 395–399. doi:10.1017/S0031182000064738

97. Poulin R, Curtis MA, Rau ME. Effects of *Eubothrium salvelini* (Cestoda) on the behaviour of *Cyclops vernalis* (Copepoda) and its susceptibility to fish predators. Parasitology. 1992;105: 265–271. doi:10.1017/S0031182000074199

98. Rode NO, Lievens EJP, Flaven E, Segard A, Jabbour-Zahab R, Sanchez MI, et al. Why join groups? Lessons from parasite-manipulated *Artemia*. Ecol Lett. 2013;16: 493–501. doi:10.1111/ele.12074

99. Sánchez MI, Varo N, Matesanz C, Ramo C, Amat JA, Green AJ. Cestodes change the isotopic signature of brine shrimp, Artemia, hosts: Implications for aquatic food webs. Int J Parasitol. 2013;43: 73–80. doi:10.1016/j.ijpara.2012.11.003

100. Blankespoor CL, Pappas PW, Eisner T. Impairment of the chemical defence of the beetle, *Tenebrio molitor*, by metacestodes (cysticercoids) of the tapeworm, *Hymenolepis diminuta*. Parasitology. 1997;115: 105–110. doi:10.1017/S0031182097008901

101. Webster JP, Gowtage-Sequeira S, Berdoy M, Hurd H. Predation of beetles (*Tenebrio molitor*) infected with tapeworms (*Hymenolepis diminuta*): A note of caution for the Manipulation Hypothesis. Parasitology. 2000;120: 313–318. doi:10.1017/S003118209900548X

102. Hurd H, Fogo S. Changes induced by *Hymenolepis diminuta* (Cestoda) in the behaviour of the intermediate host *Tenebrio molitor* (Coleoptera). Can J Zool. 1991;69: 2291–2294. doi:10.1139/z91-321

103. Yan G, Stevens L, Schall JJ. Behavioral changes in *Tribolium* beetles infected with a tapeworm: Variation in effects between beetle species and among genetic strains. Am Nat. 1994;143: 830–847.

104. Yan G, Phillips TW. Influence of tapeworm infection on the production of aggregation pheromone and defensive compounds in *Tribolium castaneum*. J Parasitol. 1996;82: 1037–1039.

105. Robb T, Reid ML. Parasite-induced changes in the behaviour of cestode-infected beetles: adaptation or simple pathology? Can J Zool. 1996;74: 1268–1274.

106. Gabagambi NP, Salvanes A-GV, Midtoy F, Skorping A. The tapeworm *Ligula intestinalis* alters the behavior of the fish intermediate host *Engraulicypris sardella*, but only after it has become infective to the final host. Behav Processes. 2019;158: 47–52. doi:10.1016/j.beproc.2018.11.002

107. Barber I, Huntingford FA. Parasite infection alters schooling behaviour: Deviant positioning of helminth-infected minnows in conspecific groups. Proceeding R Soc B. 1996;263: 1095–1102. doi:10.1098/rspb.1996.0161

108. Loot G, Aulagnier S, Lek S, Thomas F, Guégan JF. Experimental demonstration of a behavioural modification in a cyprinid fish, *Rutilus rutilus* (L.), induced by a parasite, *Ligula intestinalis* (L.). Can J Zool. 2002;80: 738–744. doi:10.1139/Z02-043

109. Loot G, Brosse S, Lek S, Guégan JF. Behaviour of roach (*Rutilus rutilus* L.) altered by *Ligula intestinalis* (Cestoda: Pseudophyllidea): a field demonstration. Freshw Biol. 2001;46: 1219–1227. doi:10.1046/j.1365-2427.2001.00733.x

110. Urdal K, Tierney JF, Jakobsen PJ. The tapeworm *Schistocephalus solidus* alters the activity and response, but not the predation susceptibility of infected copepods. J Parasitol. 1995;81: 330–333. doi:10.2307/3283949

111. Hafer N, Milinski M. When parasites disagree: Evidence for parasite-induced sabotage of host manipulation. Evolution. 2015;69: 611–620. doi:10.1111/evo.12612

112. Benesh DP. What are the evolutionary constraints on larval growth in a trophically transmitted parasite? Oecologia. 2010;162: 599–608. doi:10.1007/s00442-009-1507-6

113. Benesh D, Benesh DP. Tapeworm manipulation of copepod behaviour: parasite genotype has a larger effect than host genotype. Biol Lett. 2019;15. doi:10.1098/rsbl.2019.0495

114. Hafer-Hahmann N. Behavior out of control: Experimental evolution of resistance to host manipulation. Ecol Evol. 2019;9: 7237–7245. doi:10.1002/ece3.5294

115. Hafer N. Differences between populations in host manipulation by the tapeworm *Schistocephalus solidus* - is there local adaptation? Parasitology. 2018;145: 762–769. doi:10.1017/S0031182017001792

116. Hammerschmidt K, Koch K, Milinski M, Chubb JC, Parker GA. When to go: optimization of host switching in parasites with complex life cycles. Evolution. 2009;63: 1976–1986. doi:10.1111/j.1558-5646.2009.00687.x

117. Jakobsen PJ, Wedekind C. Copepod reaction to odor stimuli influenced by cestode infection. Behav Ecol. 1998;9: 414–418. doi:10.1093/beheco/9.4.414

118. Wedekind C, Milinski M. Do three-spined sticklebacks avoid consuming copepods, the first intermediate host of *Schistocephalus solidus*?—An experimental analysis of behavioural resistance. Parasitology. 1996;112: 371–383. doi:10.1017/S0031182000066609

119. Hafer N, Milinski M. Inter- and intraspecific conflicts between parasites over host manipulation. Proc R Soc B. 2016;283: 20152870–20152870. doi:10.1098/rspb.2015.2870

120. Hafer-Hahmann N. Experimental evolution of parasitic host manipulation. Proc R Soc B. 2019;286: 20182413–20182413. doi:10.1098/rspb.2018.2413

121. Weinreich F, Benesh DP, Milinski M. Suppression of predation on the intermediate host by two trophically-transmitted parasites when uninfective. Parasitology. 2013;140: 129–135. doi:10.1017/S0031182012001266

122. Berger CS, Aubin-Horth N. The secretome of a parasite alters its host's behaviour but does not recapitulate the behavioural response to infection. Proceeding R Soc B. 2020;287. doi:10.1098/rspb.2020.0412

123. Alves VA, Aubin-Horth N. Implication of the myo-inositol pathway in behavioural alterations of infected threespine sticklebacks. Funct Ecol. 2023;37: 873–885. doi:10.1111/1365-2435.14216

124. Demandt N, Saus B, Kurvers RHJM, Krause J, Kurtz J, Scharsack JP. Parasite-infected sticklebacks increase the risk-taking behaviour of uninfected group members. Proceeding R Soc B. 2018;285. doi:10.1098/rspb.2018.0956

125. Demandt N, Bierbach D, Kurvers RHJM, Krause J, Kurtz J, Scharsack JP. Parasite infection impairs the shoaling behaviour of uninfected shoal members under predator attack. Behav Ecol Sociobiol. 2021;75. doi:10.1007/s00265-021-03080-7

126. Giles N. Behavioural effects of the parasite *Schistocephalus solidus* (Cestoda) on an intermediate host, the three-spined stickleback, *Gasterosteus aculeatus* L. Anim Behav. 1983;31: 1192–1194. doi:10.1016/S0003-3472(83)80025-6

127. Grecias L, Valentin J, Aubin-Horth N. Testing the parasite mass burden effect on alteration of host behaviour in the *Schistocephalus*-stickleback system. J Exp Biol. 2018;221. doi:10.1242/jeb.174748

128. Jakobsen PJ, Johnsen GH, Larsson P. Effects of predation risk and parasitism on the feeding ecology, habitat use, and abundance of lacustrine threespine stickleback (*Gasterosteus aculeatus*). Can J Fish Aquat Sci. 1988;45: 426–431.

129. Jolles JW, Mazué GPF, Davidson J, Behrmann-Godel J, Couzin ID. *Schistocephalus parasite* infection alters sticklebacks' movement ability and thereby shapes social interactions. Sci Rep. 2019; 849737–849737. doi:10.1101/849737

130. LoBue CP, Bell MA. Phenotypic manipulation by the cestode parasite *Schistocephalus solidus* of its intermediate host, *Gasterosteus aculeatus*, the threespine stickleback. Am Nat. 1993;142: 725–735.

131. Milinski M. Risk of predation of parasitized sticklebacks (*Gasterosteus aculeatus* L.) under competition for food. Behaviour. 1985;93: 203–216. doi:10.1163/156853986X00883

132. Ness JH, Foster SA. Parasite-associated phenotype modifications in threespine stickleback. Oikos. 1999;85: 127–134.

133. Svensson PA, Eghbal R, Eriksson R, Nilsson E. How cunning is the puppet-master? Cestode-infected fish appear generally fearless. Parasitol Res. 2022;121: 1305–1315. doi:10.1007/s00436-022-07470-2

134. Tibblin P, Hall M, Svensson PA, Merila J, Forsman A. Phenotypic flexibility in background-mediated color change in sticklebacks. Behav Ecol. 2020;31: 950–959. doi:10.1093/beheco/araa041

135. Hafer N, Milinski M. An experimental conflict of interest between parasites reveals the mechanism of host manipulation. Behav Ecol. 2015;27: 617–627. doi:10.1093/beheco/arv200

136. Barber I, Walker P, Svensson PA. Behavioural responses to simulated avian predation in female three spined sticklebacks: the effect of experimental *Schistocephalus solidus* infections. Behaviour. 2004;141: 1425–1440. doi:10.1163/1568539042948231

137. Aeschlimann P, Häberli MA, Milinski M. Threat-sensitive feeding strategy of immature sticklebacks (*Gasterosteus aculeatus*) in response to recent experimental infection with the cestode *Schistocephalus solidus*. Behav Ecol Sociobiol. 2000;49: 1–7. doi:10.1007/s002650000273

138. Grécias L, Hébert F-O, Berger CS, Barber I, Aubin-horth N. Can the behaviour of threespine stickleback parasitized with *Schistocephalus solidus* be replicated by manipulating host physiology? J Exp Biol. 2017;220: 237–246. doi:10.1242/jeb.151456

139. Morales-Montor J, Picazo O, Besedovsky H, Hernández-Bello R, Lopez-Griego L, Becerril-Villanueva E, et al. Helminth infection alters mood and short-term memory as well as levels of neurotransmitters and cytokines in the mouse hippocampus. NeuroImmunoModulation. 2014;21: 195–205. doi:10.1159/000356521

140. Parasite-altered host behavior in the face of a predator: manipulation or not?

141. Arias-Hernández D, Flores-Pérez FI, Domínguez-Roldan R, Báez-Saldaña A, Carreon RA, García-Jiménez S, et al. Influence of the interaction between cysticercosis and obesity on rabbit behavior and productive parameters. Vet Parasitol. 2019;276: 108964. doi:10.1016/j.vetpar.2019.108964

142. Pulkkinen K, Pasternak AF, Hasu T, Valtonen ET. Effect of *Triaenophorus crassus* (Cestoda) infection on behavior and susceptibility to predation of the first intermediate host *Cyclops strenuus* (Copepoda). J Parasitol. 2000;86: 664–670. doi:10.1645/0022-3395(2000)086[0664:EOTCCI]2.0.CO;2

143. Zoratto F, Ciabattoni F, Ledda E, Racca A, Carlini A, Santucci D, et al. Behavioural changes in farmed sea bass (*Dicentrarchus labrax*) experimentally infected by *Anisakis* nematodes. Rendiconti Lincei Sci Fis E Nat. 2022;33: 555–567. doi:10.1007/s12210-022-01087-4 EA JUL 2022

144. Moore J, Lasswell J. Altered Behavior in Isopods (*Armadillidium vulgare*) Infected with the Nematode *Dispharynx nasuta*. J Parasitol. 1986;72: 186. doi:10.2307/3281818

145. Verble RM, Meyer AD, Kleve MG, Yanoviak SP. Exoskeletal thinning in *Cephalotes atratus* ants (Hymenoptera: Formicidae) parasitized by *Myrmeconema neotropicum* (Nematoda: Tetradonematidae). J Parasitol. 2012;98: 226–228. doi:10.1645/GE-2847.1

146. Yanoviak SP, Kaspari M, Dudley R, Poinar G Jr. Parasite-induced fruit mimicry in a tropical canopy ant. Am Nat. 2008;171: 536–544. doi:10.1086/528968

147. McCoy KD, Nudds TD. An examination of the manipulation hypothesis to explain prevalence of *Parelaphostrongylus tenuis* in gastropod intermediate host populations. Can J Zool. 2000;78: 294–299. doi:10.1139/cjz-78-2-294

148. Schutgens M, Cook B, Gilbert F, Behnke JMM. Behavioural changes in the flour beetle *Tribolium confusum* infected with the spirurid nematode *Protospirura muricola*. J Helminthol. 2015;89: 68–79. doi:10.1017/S0022149X13000606

149. Luong LT, Hudson PJ, Braithwaite VA. Parasite-induced changes in the anti-predator behavior of a cricket intermediate host. Ethology. 2011;117: 1019–1026. doi:10.1111/j.1439-0310.2011.01951.x

150. Mccurdy DG, Forbes MR, Sherman Boates J. Evidence that the parasitic nematode *Skrjabinoclava* manipulates host *Corophium* behavior to increase transmission to the sandpiper, *Calidris pusilla*. Behav Ecol. 10: 351–357.

151. Cox DM, Holland CV. The relationship between numbers of larvae recovered from the brain of *Toxocara canis*-infected mice and social behaviour and anxiety in the host. Parasitology. 1998;116: 579–594.

152. Janecek E, Waindok P, Bankstahl M, Strube C. Abnormal neurobehaviour and impaired memory function as a consequence of *Toxocara canis*- as well as *Toxocara cati*-induced neurotoxocarosis. PLoS Negl Trop Dis. 2017;11: 1–20. doi:10.1371/journal.pntd.0005594

153. Hay J, Aitken PP. Experimental toxocariasis in mice and its effect on their behaviour. Ann Trop Med Parasitol. 1984;78: 145–155. doi:10.1080/00034983.1984.11811788

154. de Queiroz ML, Viel TA, Papa CHG, Lescano SAZ, Chieffi PP. Behavioral changes in *Rattus norvegicus* coinfected by *Toxocara canis* and *Toxoplasma gondii*. Rev Inst Med Trop Sao Paulo. 2013;55: 51–53. doi:10.1590/S0036-46652013000100009

155. Santos EGN, Cunha RA, Santos Potes CP. Behavioral responses of *Poecilia vivipara* (Osteichthyies: Cyprinodontiformes) to experimental infections of *Acanthocollaritrema umbilicatum* (Digenea: Cryptogonimidae). Exp Parasitol. 2011;127: 522–526. doi:10.1016/j.exppara.2010.10.018

156. Zhokhov AE, Pugacheva MN, Mikheev VN. Decalcification of a clam shell caused by trematodes: Side effect or manipulation of the host phenotype? Contemp Probl Ecol. 2024;17: 54–60. doi:10.1134/S1995425524010165

157. Nezhybova V, Janac M, Reichard M, Ondrackova M. Risk-taking behaviour in African killifish - a case of parasitic manipulation? J Vertebr Biol. 2020;69. doi:10.25225/jvb.20022

158. Santos EGN, Portes Santos C. Parasite-induced and parasite development-dependent alteration of the swimming behavior of fish hosts. Acta Trop. 2013;127: 56–62. doi:10.1016/j.actatropica.2013.03.008

159. Feijen F, Buser C, Klappert K, Jokela J. Parasite infection and the movement of the aquatic snail *Potamopyrgus antipodarum* along a depth cline. Ecol Evol. 2023;13. doi:10.1002/ece3.10124

160. Karvonen A, Faltynkova A, Choo JM, Valtonen ET. Infection, specificity and host manipulation of *Australapatemon* sp (Trematoda, Strigeidae) in two sympatric species of leeches (Hirudinea). Parasitology. 2017;144: 1346–1355. doi:10.1017/S0031182017000609

161. Poulin R. Progenesis and reduced virulence as an alternative transmission strategy in a parasitic trematode. Parasitology. 2001;123: 623–630. doi:10.1017/s0031182001008794

162. Coats J, Poulin R, Nakagawa S. The consequences of parasitic infections for host behavioural correlations and repeatability. Behaviour. 2010;147: 367–382. doi:10.1163/000579509X12574307194101

163. Krause J, Godin JGJ. Influence of parasitism on the shoaling behavior of banded killifish, *Fundulus diaphanus*. Can J Zool. 1994;72: 1775–1779. doi:10.1139/z94-240

164. Mouritsen KN, Poulin R. Parasite-induced trophic facilitation exploited by a non-host predator: a manipulator’s nightmare. Int J Parasitol. 2003;33: 1043–1050. doi:10.1016/S0020-7519(03)00178-4

165. Franco-Bodek T, Barradas-Ortiz C, Negrete-Soto F, Rodriguez-Canul R, Lozano-Alvarez E, Briones-Fourzan P. Effects of *Cymatocarpus solearis* (Trematoda: Brachycoeliidae) on its second intermediate host, the Caribbean spiny lobster *Panulirus argus*. PLoS One. 2023;18. doi:10.1371/journal.pone.0287097

166. Pearre S. Niche Modification in Chaetognatha Infected with Larval Trematodes (Digenea). Int Rev Gesamten Hydrobiol Hydrogr. 1979;64: 193–206. doi:10.1002/iroh.19790640205

167. Gopko M, Mikheev VN, Taskinen J. Deterioration of basic components of the anti-predator behavior in fish harboring eye fluke larvae. Behav Ecol Sociobiol. 2017;71: 68–68. doi:10.1007/s00265-017-2300-x

168. Klemme I, Kortet R, Karvonen A. Parasite infection in a central sensory organ of fish does not affect host personality. Behav Ecol. 2016;27: 1533–1538. doi:10.1093/beheco/arw080

169. Mikheev VN, Pasternak AF. Structure of Aggressive Behavior in Underyearlings of the Rainbow Trout *Oncorhynchus mykiss* (Salmonidae) Changes under the Influence of *Diplostomum pseudospathaceum* (Trematoda) Parasites. J Ichthyol. 2023;63: 816–821. doi:10.1134/S0032945223040136

170. Gopko M, Mikheev VN, Taskinen J. Changes in host behaviour caused by immature larvae of the eye fluke: evidence supporting the predation suppression hypothesis. Behav Ecol Sociobiol. 2015;69: 1723–1730. doi:10.1007/s00265-015-1984-z

171. Gopko M, Tkachenko D, Shpagina A, Maximenko D, Mironova E. Is vision deterioration responsible for changes in the host's behavior caused by eye flukes? Int J Parasitol. 2023;53: 731–738. doi:10.1016/j.ijpara.2023.06.001

172. Mikheev VN, Pasternak AF, Taskinen J, Valtonen ET. Parasite-induced aggression and impaired contest ability in a fish host. Parasit Vectors. 2010;3: 17–17. doi:10.1186/1756-3305-3-17

173. Seppälä O, Karvonen A, Valtonen ET. Parasite-induced change in host behaviour and susceptibility to predation in an eye fluke-fish interaction. Anim Behav. 2004;68: 257–263.

174. Seppälä O, Karvonen A, Valtonen ET. Manipulation of fish host by eye flukes in relation to cataract formation and parasite infectivity. Anim Behav. 2005;70: 889–894. doi:10.1016/j.anbehav.2005.01.020

175. Seppälä O, Karvonen A, Valtonen ET. Impaired crypsis of fish infected with a trophically transmitted parasite. Anim Behav. 2005;70: 895–900. doi:10.1016/j.anbehav.2005.01.021

176. Seppälä O, Karvonen A, Valtonen ET. Susceptibility of eye fluke-infected fish to predation by bird hosts. Parasitology. 2006;132: 575–579. doi:10.1017/S0031182005009431

177. Seppälä O, Karvonen A, Valtonen ET, Seppaelae O, Karvonen A, Valtonen ET, et al. Behavioural mechanisms underlying `specific' host manipulation by a trophically transmitted parasite. Evol Ecol Res. 2012;14: 73–81.

178. Lafferty KD, Morris AK. Altered behavior of parasitized killifish increases susceptibility to predation by bird final hosts. Ecology. 1996;77: 1390–1397. doi:10.2307/2265536

179. Weinersmith KL, Nadler LE, Bengston E, Turner AV, Birda A, Cobian K, et al. Experimental infections with *Euhaplorchis clifornienesis* and a small cythocotylid increase conspicuous behaviors in California killifish (*Fundulus parvipinnis*). J Parasitol. 2023;109: 362–376. doi:10.1645/23-35

180. McCurdy DG, Forbes MR, Boates JS. Testing alternative hypotheses for variation in amphipod behaviour and life history in relation to parasitism. Int J Parasitol. 1999;29: 1001–1009. doi:10.1016/S0020-7519(99)00067-3

181. Wesołowska W, Wesołowski T. Do *Leucochloridium* sporocysts manipulate the behaviour of their snail hosts? J Zool. 2014;292: 151–155. doi:10.1111/jzo.12094

182. Johnson DS. Are amphipods *Orchestia grillus* (Bosc, 1802) (Amphipoda: Talitridae) infected with the trematode *Levinseniella byrdi* (Heard, 1968) drawn to the light? J Crustac Biol. 2022;42: 1–6. doi:10.1093/jcbiol/ruac017

183. Leung TLF, Poulin R. Effects of the trematode *Maritrema novaezealandensis* on the behaviour of its amphipod host: adaptive or not? J Helminthol. 2006;80: 271–275. doi:10.1079/JOH2005332

184. Huxhamat M, Raffaelli D, Pikeb AW. The effect of larval trematodes on the growth and burrowing behaviour of *Hydrobia ulvae* (gastropoda: prosobranchiata) in the Ythan estuary, north-east Scotland. J Exp Mar Biol Ecol. 1995;185: 1–17.

185. Hansen EK, Poulin R. Impact of a microphallid trematode on the behaviour and survival of its isopod intermediate host: phylogenetic inheritance? Parasitol Res. 2005;97: 242–246. doi:10.1007/s00436-005-1435-2

186. Reisinger LS, Petersen I, Hing JS, Davila RL, Lodge DM. Infection with a trematode parasite differentially alters competitive interactions and antipredator behaviour in native and invasive crayfish. Freshw Biol. 2015;60: 1581–1595. doi:10.1111/fwb.12590

187. Reisinger LS, Lodge DM. Parasites alter freshwater communities in mesocosms by modifying invasive crayfish behavior. Ecology. 2016;97: 1497–1506. doi:10.1890/15-1634.1

188. Levri EP, Lunnen SJ, Itle CT, Mosquea L, Kinkade BV, Martin TG, et al. Parasite-induced alteration of diurnal rhythms in a freshwater snail. J Parasitol. 2007;93: 231–237. doi:10.1645/GE-933R1.1

189. Levri EP, Lively CM. The effects of size, reproductive condition, and parasitism on foraging behaviour in a freshwater snail, *Potamopyrgus antipodarum*. Anim Behav. 1996;51: 891–901. doi:10.1006/anbe.1996.0093

190. Levri EP, Fisher LM. The effect of a trematode parasite (*Microphallus* sp.) on the response of the freshwater snail *Potamopygrus antipodarum* to light and gravity. Behaviour. 137: 1141–1151.

191. Helluy S. Host-parasite relations of the trematode *Microphallus papillorobustus* (Rankin 1940). III Factors involved in the behavioral changes of the *Gammarus*, intermediate hosts and predator tests. Ann Parasitol Hum Comparée. 1984;59: 41–56. doi:10.1051/parasite/1984591041

192. Thomas F, Mete K, Helluy S, Santalla F, Verneau O, de Meeus T, et al. Hitch-hiker parasites or how to benefit from the strategy of another parasite. Evolution. 1997;51: 1316–1318.

193. McCarthy HO, Fitzpatrick S, Irwin SWB. A transmissible trematode affects the direction and rhythm of movement in a marine gastropod. Anim Behav. 2000;59: 1161–1166. doi:10.1006/anbe.2000.1414

194. Ro H, Fowler AE, Wood CL, Blakeslee AMH. Trematode parasites have minimal effect on the behavior of invasive green crabs. Aquat Invasions. 2022;17: 238–258. doi:10.3391/ai.2022.17.2.07

195. Blakeslee AMH, Keogh CL, Fowler AE, Griffen BD. Assessing the effects of trematode infection on invasive green crabs in eastern North America. PLoS One. 2015;10. doi:10.1371/journal.pone.0128674

196. Khan RN, Spiers JA, Pung OJ. Effects of the trematode *Microphallus turgidus* on locomotion and prey capture in the grass shrimp *Palaemonetes pugio*. J Helminthol. 2003;77: 327–330. doi:10.1079/joh2003192

197. Kunz AK, Pung OJ. Effects of *Microphallus turgidus* (Trematoda: Microphallidae) on the predation, behavior, and swimming stamina of the grass shrimp *Palaemonetes pugio*. J Parasitol. 2004;90: 441–445. doi:10.1645/GE-183R

198. Gonzalez ST. Influence of a Trematode Parasite (*Microphallus turgidus*) on Grass Shrimp (*Palaemonetes pugio*) Response to Refuge and Predator Presence. J Parasitol. 2016;102: 646–649. doi:10.1645/15-889

199. Sacco LH, Goater CP, Smith T-D, Chivers DP, Ferrari MCO. Escape responses to simulated host versus nonhost predators in minnows exposed to a brain-encysting parasite. Anim Behav. 2021;173: 169–176. doi:10.1016/j.anbehav.2021.01.006

200. Shirakashi S, Goater C, Shirakashi S, Goater C. Brain-encysting parasites affect visually-mediated behaviours of fathead minnows. Ecoscience. 2001;8: 289–293. doi:10.1080/11956860.2001.11682655

201. Shirakashi S, Goater CP. Chronology of parasite-induced alteration of fish behaviour: Effects of parasite maturation and host experience. Parasitology. 2005;130: 177–183. doi:10.1017/S0031182004006432

202. Shirakashi S, Goater CP. Intensity-dependent alteration of minnow (*Pimephales promelas*) behavior by a brain-encysting trematode. J Parasitol. 2002;88: 1071–1074. doi:10.1645/0022-3395(2002)088[1071:idaomp]2.0.co;2

203. Edelaar P, Drent J, De Goeij P. A double test of the parasite manipulation hypothesis in a burrowing bivalve. Oecologia. 2003;134: 66–71. doi:10.1007/s00442-002-1038-x

204. Preston DL, Boland CE, Hoverman JT, Johnson PTJ. Natural enemy ecology: comparing the effects of predation risk, infection risk and disease on host behaviour. Funct Ecol. 2014;28: 1472–1481. doi:10.1111/1365-2435.12293

205. Ruehle R, Poulin R. Potential multidimensional behavioural impacts of differential infection in two fish populations. Behaviour. 2020;157: 901–922. doi:10.1163/1568539X-bja10029

206. López-Rodríguez R, George-Nascimento M, Górski K, Lopez-Rodriguez R, George-Nascimento M, Gorski K. Effects of the cranial parasite *Tylodelphys* sp. on the behavior and physiology of puye *Galaxias maculatus* (Jenyns, 1842). PEERJ. 2021;9. doi:10.7717/peerj.11095

207. Muñoz JCV, Bierbach D, Knopf K. Eye fluke (*Tylodelphys clavata*) infection impairs visual ability and hampers foraging success in European perch. Parasitol Res. 2019;118: 2531–2541. doi:10.1007/s00436-019-06389-5
