# Supplementary material for: The Evolution and Ecology of Host Manipulation in Helminth Parasites: A Phylogenetic Meta‐Analysis
Source: Ecol Lett. 2026 Feb 18;29(2):e70340. doi: 10.1111/ele.70340 (PMC12916080; doi:10.1111/ele.70340)
Supplement: Supplementary file 6 — Table S2: Additional traits investigated. Table S3: Model diagnostics. Please see Table 2 for ELPD loo and model comparisons. R‐hat was close to 1 (< 1.01) or all models. The number of problematic pareto k values never exceeded 2% of all observations. Table S4: Model diagnostics for models investigating factors that may drive or constrain the evolution of host manipulation. Please see Table 4 for ELPD loo and model comparisons. R‐hat was close to 1 (< 1.01) for all models. The number of problematic pareto k values never exceeded 2% of all observations. Table S5: Model diagnostics for models investigating the interaction between behaviour and factors that may drive or constrain the evolution of host manipulation. Loo was obtained with moment match set to true. Please see Table S11 for ELPD loo and model comparisons. The number of problematic pareto k values never exceeded 3% of all observations. For immature parasites predator was included for the basic model but not for the one without outliers since it did not clearly improve that model. Table S6: Results of model comparisons using leave‐one‐out cross‐validation (LOO) and model diagnostics for predation susceptibility. Results were obtained through the Bayesian modelling framework (BRMS). Factors that clearly improved the model are indicated in bold (i.e., difference in ELPD larger than two times SE), those for which an improvement was observed, but was unclear due to the difference in ELPD being between one and two times SE are highlighted in italics. Comparisons are always to the preceding model that was clearly better (i.e., highlighted in bold). All models included the following random effects: Paper ID, Host and parasite phylogeny and the interaction between host and parasite. R‐hat was close to 1 (< 1.01) for all models. Please note that a substantial number of problematic pareto k values were occurred. Hence ELPD loo may be unreliable and any comparison should be viewed with caution. Table S7: Estimated marg [file ELE-29-0-s003.pdf]

Supplementary tables

Table S2: Additional traits investigated.

**Trait**	**Details/ Levels**	**Remarks & expectation**
Host life span	log transformed	Expectations: Higher PE in short lived hosts; no effect on PS
Adult parasite size	Proxy for fitness; log transformed	Expectation: Trade-off between host manipulation and reproductive output, i.e. higher host manipulation in hosts with lower fecundity; requires host manipulation to be energetically costly.
Proportion of trophic interactions as predator*	Proxy for predation susceptibility; arcsine transformed	Expectation: Animals with more interactions as a predator should be less susceptible to predation; PE: positive correlation between interactions as predator and host manipulation; PS: negative correlation between interactions as predator and host manipulation
Trophic level	Proxy for predation susceptibility	Expectation: Animals with higher trophic levels should be less susceptible to predation; PE: positive correlation trophic level and host manipulation; PS: negative correlation between trophic level and host manipulation
Length	Proxy for predation susceptibility; log transformed	Expectations: Negative correlation between size and predation susceptibility; PE: Higher host manipulation by lager hosts; PS: Higher host manipulation by smaller hosts.
Parasite group	Ancanthocephala, Cestoda, Nematoda, Trematoda	partly captured by phylogeny
Host group	Spiralia (molluscs, annelids, Chaetognatha), terrestrial vertebrates, crustaceans, insects, fish	Crustacea included as a group even so it is not a monophyletic group; partly captured by phylogeny
Next host type	Aquatic vertebrate (fish and cetaceans), terrestrial vertebrate (excluding birds; including parasites that can reproduce in different vertebrates including birds and live in terrestrial intermediate hosts), birds (including parasites that can reproduce in any warm blooded host and use aquatic intermediate hosts since these usually use birds as final hosts in nature)	Transmission to different host may require the modification of different traits
Trophic link	aquatic crustaceans (without zooplankton) to aquatic vertebrate, fish to bird, terrestrial arthropods to vertebrate, terrestrial vertebrate to vertebrate, aquatic crustaceans (without zooplankton) to bird, zooplankton to fish, annelids and molluscs to bird, annelids and molluscs to non-bird vertebrate, terrestrial	Transmission to different host may require the modification of different traits which may depend on the type of current host exploited.

	arthropods to bird, zooplankton to bird, fish to vertebrate(without birds)	
Host habitat	Aquatic, terrestrial	Efficiency of host manipulation and of modifying different behaviors may vary among different habitats
Infection site		Infection site may influence which behaviours and how strongly parasites are able to manipulate

* The proportion of interactions as predator was obtained by querying globi in R (Poelen et al. 2025, *rglobi: Interface to Global Biotic Interactions*. https://docs.ropensci.org/rglobi/).and extracting interactions classified as “eatenBy” and “preyedUponBy”. Since globi varies strongly in the amount of information available for different taxa, I also obtained the number of total trophic interactions by additionally obtaining interactions classified as “preysOn” and “eats” and calculated the proportion of interactions as predator out of all interactions. In order to ensure sufficient amount of data, I obtained genus level data and if less than 10 trophic interactions were available for a genus, I used family level data instead.
PE: Predation enhancement by mature parasites; PS: Predation suppression by immature parasites

Table S3: Model diagnostics. Please see table 2 for ELPD loo and model comparisons. R-hat was close to 1 (<1.01) for all models. The number of problematic pareto k values never exceeded 2% of all observations.

	basic models				outliers excluded			
model	Bulk ESS, min	Bulk ESS, mean	N pareto k >0.7 (k>1)	Max. pareto k	Bulk ESS, min	Bulk ESS, mean	N pareto k >0.7	Max. pareto k
**A: All parasites**								
SE + parasite stage + predator + predator:parasite stage	1967	13789	4	0.84	2545	17495	3	0.88
SE + parasite stage + predator	2151	17022	3	0.88	2386	16247	1	0.74
SE + parasite stage + behavior	2734	16835	0	0.69	2303	13765	0	0.60
SE + parasite stage + infection	3564	12799	2	0.73	2971	11242	0	0.54
SE + parasite stage + year	2539	14404	0	0.55	2539	14404	0	0.55
SE + parasite stage	2173	12764	7	0.76	2276	13367	0	0.66
SE	3331	15226	3	0.80	2256	9001	1	0.74
1	2074	10413	3 (1)	1.46	3517	13474	1	0.70
**B: Mature parasites**								
SE + predator	3503	17319	5	0.92	2360	11779	3	0.83
SE + behavior	3300	16870	0	0.67	2729	12288	1	0.74
SE + infection	3674	9670	3 (1)	1.08	3191	8228	1	0.73
SE + year	2995	11657	2	0.76	3626	12870	0	0.70
SE	2650	10101	3	0.81	2965	10110	2	0.75
1	2568	11118	7 (1)	1.35	2017	10392	0	0.70
**C: Immature parasites**								
SE + predator	4119	15344	0	0.69	5471	19328	5	0.82
SE + behavior	3992	15139	0	0.49	3903	14730	1	0.71
SE + infection	3845	16059	0	0.67	3382	10086	4	0.96
SE + year	3963	11709	0	0.61	4504	14129	1	0.74
SE	2650	10101	3	0.66	2965	10110	2	0.75
1	3495	9639	1	0.69	3922	9144	1	0.73

Total number of samples/ pareto k-values (basic models/ models without outliers): all parasites: 1634/ 1554; mature parasites: 1177/ 1117; immature parasites: 278/ 268

Table S4: Model diagnostics for models investigating factors that may drive or constrain the evolution of host manipulation. Please see Table 4 for ELPD loo and model comparisons. R-hat was close to 1 (<1.01) for all models. The number of problematic pareto k values never exceeded 2% of all observations.

	basic models				outliers excluded			
model	Bulk ESS, min	Bulk ESS, mean	N pareto k >0.7 (k>1)	Max. pareto k	Bulk ESS, min	Bulk ESS, mean	N pareto k >0.7 (k>1)	Max. pareto k
**A: Mature parasites**								
+ trophic link	3235	13820	3	0.84	2853	10167	1	0.73
+ next host type	2550	12410	7	0.94	2269	11637	1	0.73
+ adult parasite size	2778	11691	1	0.76	2724	11769	1	0.73
+ infection site	2367	13514	4 (1)	1.01	2961	13594	1	0.81
+ parasite type	3232	16958	5 (2)	1.33	3314	18172	1	0.80
+ trophic interactions as predator (arcsine transformed)	3464	18049	2	0.72	2552	11767	1	0.79
+ trophic level	3340	18351	7	0.89	3032	11945	2	0.84
+ host life span (log transformed)	3434	14336	5 (4)	1.44	3267	17663	1	0.83
+ host size (log transformed)	3062	15089	8 (3)	1.08	2966	14966	1	0.74
+ host type	2860	12413	5 (5)	1.42	2466	11751	2	0.74
+ habitat	2638	12234	5 (5)	1.19	3749	18862	2	0.76
SE + predator	3503	17319	5	0.92	2360	11779	3	0.83
**B: Immature parasites**								
+ trophic link	4157	20587	1	0.75	4202	18048	3	0.76
+ next host type	4169	15879	1	0.71	5490	22814	4	0.93
+ adult parasite size	4333	16794	1	0.70	4947	21276	3	0.89
+ infection site	3801	21867	1	0.75	4473	17146	4 (1)	1.09
+ parasite type	5294	21080	0	0.65	4681	18649	5	0.87
+ trophic interactions as predator (arcsine transformed)	4329	17692	0	0.66	3753	14219	4	0.87
+ trophic level	4685	18323	0	0.61	4700	20158	4	0.92
+ host life span (log transformed)	4727	18470	1	0.71	4949	17939	5	0.80
+ host size (log transformed)	4889	22011	1	0.73	5013	18090	4	0.81
+ host type	5259	20470	1	0.72	4321	17549	5	0.84
+ habitat	5065	19745	0	0.69	4754	17437	3	0.89
SE + predator	4119	15344	0	0.69	5471	19328	5	0.82

Total number of samples/ pareto k-values (basic models/ models without outliers): mature parasites: 1177/ 1117; immature parasites: 278/ 268.

Table S5: Model diagnostics for models investigating the interaction between behaviour and factors that may drive or constrain the evolution of host manipulation. Loo was obtained with moment match set to true. Please see Table S11 for ELPD loo and model comparisons. The number of problematic pareto k values never exceeded 3% of all observations. For immature parasites predator was included for the basic model but not for the one without outliers since it did not clearly improve that model.

	basic models				outliers excluded			
model	Bulk ESS, min	Bulk ESS, mean	N pareto k >0.7	Max. pareto k	Bulk ESS, min	Bulk ESS, mean	N pareto k >0.7 (k>1)	Max. pareto k
**A: Mature parasites**								
+ behavior:trophic link	3732	19690	2	1.00	3990	19302	3 (1)	1.03
+ behavior:next host type	3150	12841	0	0.68	2995	11384	2 (1)	1.23
+ behavior:infection site	3143	12314	0	0.66	3778	13745	2	0.79
+ behavior:parasite type	2750	11544	0	0.70	2998	12409	1	0.70
+ behavior:host type	4083	20165	0	0.61	3987	15735	1	0.70
+ behavior:habitat	4142	16079	4	0.85	2882	10596	3 (1)	1.01
**SE + behaviour + predator**	3612	15760	0	0.5	3706	15680	1	0.77
**B: Immature parasites**								
+ behavior:trophic link	3732	19690	3	0.88	5092	18698	4	0.95
+ behavior:next host type	5431	16699	4	0.81	5815	20340	5	0.75
+ behavior:infection site	6734	21322	1	0.75	4327	15947	4	0.76
+ behavior:parasite type	7188	25674	5	0.95	5286	20910	8	0.92
+ behavior:host type	6596	24591	1	0.77	5497	23479	4	0.88
+ behavior:habitat	4393	16719	2	0.74	5141	20226	4	0.88
**SE + behaviour (+predator)**	4991	17519	0	0.68	3903	14730	1	0.71

Total number of samples/ pareto k-values (basic models/ models without outliers): all parasites: 1634/ 1554; mature parasites: 1177/ 1117; immature parasites: 278/ 268. ESS: Effective samplings size.

Table S6: Results of model comparisons using Leave-One-Out Cross-Validation (LOO) and model diagnostics for predation susceptibility. Results were obtained through the Bayesian modelling framework (BRMS). Factors that clearly improved the model are indicated in bold (i.e. difference in ELPD larger than two times SE), those for which an improvement was observed, but was unclear due to the difference in ELPD being between one and two times SE are highlighted in italics. Comparisons are always to the preceding model that was clearly better (i.e. highlighted in bold). All models included the following random effects: Paper ID, Host and parasite phylogeny and the interaction between host and parasite. R-hat was close to 1 (<1.01) for all models. Please note that a substantial number of problematic pareto k values were occurred. Hence ELPD loo may be unreliable and any comparison should be viewed with caution.

Model (Fixed factors)	ELPD loo	p loo	ΔELPD	Δse	Bulk ESS, min	Bulk ESS, mean	N pareto k >0.7 (>1)	Max. pareto k
**A: All parasites**								
SE + parasite stage + infection	-108	52.7	-0.31	1.19	2920	9758	2	0.99
SE + parasite stage + year	*-105*	*47.2*	*3.15*	2.57	3641	11757	3 (1)	1.04
**SE + parasite stage**	**-108**	**51.2**	**15.28**	**7.43**	**2190**	**15553**	**7 (3)**	**1.45**
SE	*-123*	*55*	*2.88*	*1.81*	*2455*	*14229*	*8 (3)*	*1.61*
1	-126	53	0	0	2373	13279	8 (2)	1.10
**B: Mature parasites**								
SE + infection	-67	30.4	-0.35	0.47	3135	13001	5 (1)	1.02
SE + year	-66	29.7	0.69	1.31	2249	8730	8 (5)	1.70
**SE**	**-67**	**30.1**	5.37	2.18	3117	10519	7 (3)	1.13
1	-72	31.6	0	0	2599	9289	8 (3)	1.43
**C: Immature parasites**								
SE + infection	-4	5.3	0.75	0.45	10521	15264	4	0.95
SE + year	-5	6.9	-1.01	1.05	11585	18446	3	0.77
SE	-4	5.9	0.06	0.37	11180	16046	6	0.94
1	-4	6	0	0	11429	19251	5	1.00

ELPD loo: Expected log predictive density estimated via LOO cross-validation; p loo: Effective number of parameters, indicating model complexity; ΔELPD: Difference in ELPD loo between models; Δse: Standard error of ΔELPD, indicating uncertainty in model differences.
Number of samples/ pareto k-values: all parasites: 74; mature parasites: 65; immature parasites: 8

Table S7: Estimated marginal means and contrasts for the best model. Outliers removed.

Factor	Level	Emmean/ trend (HPD range)	N	Unique taxa
**A: Full model (all parasites)**				
Estimates for fixed factors				
Factor	Level	Emmean/ trend (HPD range)	N	Unique taxa
**overall**	**overall**	**0.459 (0.089 - 0.851)**	**1552/204**	**109(78/80)**
**SE**	**overall**	**1.357 (1.025 - 1.701)**	**1552/204**	**109(78/80)**
parasite stage	immature	0.07 (-0.309 - 0.464)	264/49	30(25/27)
**parasite stage**	**mature**	**0.709 (0.339 - 1.098)**	**1115/192**	**106(77/77)**
**parasite stage**	**mix**	**0.725 (0.329 - 1.105)**	**100/10**	**10(9/9)**
parasite stage	switching	0.33 (-0.05 - 0.728)	73/16	10(8/9)
predator	absent	0.264 (-0.116 - 0.636)	1074/179	106(75/79)
**predator**	**dead end**	**0.502 (0.084 - 0.916)**	**73/19**	**13(10/10)**
**predator**	**present**	**0.611 (0.236 - 0.993)**	**405/77**	**43(35/30)**
Contrasts				
Factor	Contrast	Estimate (HPD range)		
parasite stage	immature - mature	-0.637 (-0.729 - -0.548)		
**parasite stage**	**immature - mix**	**-0.655 (-0.781 - -0.527)**		
**parasite stage**	**immature - switching**	**-0.26 (-0.376 - -0.145)**		
parasite stage	mature - mix	-0.017 (-0.126 - 0.088)		
**parasite stage**	**mature - switching**	**0.378 (0.274 - 0.484)**		
**parasite stage**	**mix - switching**	**0.395 (0.266 - 0.529)**		
**predator**	**absent - dead end**	**-0.237 (-0.444 - -0.041)**		
**predator**	**absent - present**	**-0.348 (-0.422 - -0.273)**		
predator	dead end - present	-0.111 (-0.319 - 0.101)		
**B: Mature parasites**				
Estimates for fixed factors				
Factor	Level	Emmean/ trend (HPD range)	N	Unique taxa
**overall**	**overall**	**0.753 (0.393 - 1.113)**	**1117/193**	**107(78/78)**
**SE**	**overall**	**1.872 (1.469 - 2.259)**	**1117/193**	**107(78/78)**
**predator**	**absent**	**0.576 (0.22 - 0.929)**	**817/168**	**104(75/77)**
**predator**	**dead end**	**0.762 (0.344 - 1.153)**	**64/17**	**12(9/9)**
**predator**	**present**	**0.918 (0.543 - 1.271)**	**236/72**	**43(35/30)**
Contrasts				
Factor	Contrast	Estimate (HPD range)		
predator	absent - dead end	-0.185 (-0.414 - 0.03)		
**predator**	**absent - present**	**-0.343 (-0.445 - -0.249)**		
predator	dead end - present	-0.158 (-0.388 - 0.085)		
**C: Immature parasites**				
Estimates for fixed factors				
Factor	Level	Emmean/ trend (HPD range)	N	Unique taxa

overall	overall	-0.25 (-0.883 - 0.421)	268/49	30(25/27)
**SE**	**overall**	**-0.887 (-1.633 - -0.135)**	**268/49**	**30(25/27)**
predator	absent	-0.26 (-0.886 - 0.382)	158/38	29(24/27)
predator	dead end	-0.461 (-1.271 - 0.3)	8/1	1(1/1)
predator	present	-0.029 (-0.675 - 0.599)	102/21	11(9/9)

Contrasts

Factor	Contrast	Estimate (HPD range)
predator	absent - dead end	0.203 (-0.287 - 0.696)
**predator**	**absent - present**	**-0.231 (-0.359 - -0.099)**
predator	dead end - present	-0.436 (-0.946 - 0.061)

N indicates the number of observations/ Number of independent studies. Unique taxa indicates the number of unique host-parasite-pairs (unique host taxa/unique parasite taxa). As far as possible, these estimates are based on species. However, in a limited number of cases, in which species level identification of parasites was not available, it is based on genus. Bold rows indicate estimates or contrasts whose HPD (High posterior density) range does not overlap with 0, i.e. that estimates for these levels differ from 0 and from each other respectively.

Table S8: Estimated marginal means and contrasts. Interaction between predator stage and type of predator; full model on all parasite stages.

Estimates for fixed factors				
Factor	Level	Emmean/ trend (HPD range)	N	Unique taxa
**overall**	**overall**	**0.552 (0.062 - 1.025)**	**1634/207**	**113(80/82)**
**SE**	**overall**	**1.455 (1.093 - 1.815)**	**1634/207**	**113(80/82)**
parasite stage	immature	0.207 (-0.205 - 0.619)	278/49	30(25/27)
**parasite stage**	**mature**	**0.708 (0.322 - 1.098)**	**1177/196**	**110(79/79)**
**parasite stage**	**mix**	**0.87 (0.365 - 1.391)**	**104/11**	**11(10/10)**
parasite stage	switching	0.425 (-0.712 - 1.594)	75/16	10(8/9)
predator	absent	0.33 (-0.064 - 0.712)	1134/181	110(77/81)
predator	dead end	0.679 (-0.251 - 1.661)	79/20	13(10/10)
**predator**	**present**	**0.645 (0.245 - 1.032)**	**421/78**	**44(36/31)**
parasite stage:predator	immature absent	0.059 (-0.323 - 0.469)	166/38	29(24/27)
parasite stage:predator	immature dead end	0.4 (-0.16 - 0.979)	8/1	1(1/1)
parasite stage:predator	immature present	0.161 (-0.23 - 0.564)	104/21	11(9/9)
**parasite stage:predator**	**mature absent**	**0.504 (0.126 - 0.896)**	**865/170**	**107(76/78)**
**parasite stage:predator**	**mature dead end**	**0.651 (0.23 - 1.099)**	**70/18**	**12(9/9)**
**parasite stage:predator**	**mature present**	**0.964 (0.564 - 1.346)**	**242/73**	**44(36/31)**
**parasite stage:predator**	**mix absent**	**0.532 (0.139 - 0.949)**	**58/8**	**8(7/8)**
parasite stage:predator	mix dead end	1.104 (-0.02 - 2.128)	1/1	1(1/1)
**parasite stage:predator**	**mix present**	**0.983 (0.559 - 1.382)**	**45/4**	**4(3/3)**
parasite stage:predator	switching absent	0.225 (-0.189 - 0.632)	45/11	10(8/9)
**parasite stage:predator**	**switching present**	**0.475 (0.076 - 0.895)**	**30/9**	**5(4/4)**
Contrasts				
Factor	Contrast	Estimate (HPD range)		
**parasite stage**	**immature - mature**	**-0.498 (-0.681 - -0.322)**		
**parasite stage**	**immature - mix**	**-0.663 (-1.02 - -0.286)**		
parasite stage	immature - switching	-0.218 (-1.303 - 0.872)		
parasite stage	mature - mix	-0.166 (-0.507 - 0.201)		
parasite stage	mature - switching	0.278 (-0.793 - 1.403)		
parasite stage	mix - switching	0.446 (-0.637 - 1.628)		
predator	absent - dead end	-0.351 (-1.267 - 0.494)		
**predator**	**absent - present**	**-0.315 (-0.406 - -0.23)**		
predator	dead end - present	0.036 (-0.82 - 0.932)		
parasite stage:predator	immature absent - immature dead end	-0.342 (-0.773 - 0.065)		
parasite stage:predator	immature absent - immature present	-0.1 (-0.234 - 0.023)		

Estimates for fixed factors				
Factor	Level	Emmean/ trend (HPD range)	N	Unique taxa
parasite stage:predator	mature absent - mature dead end	-0.146 (-0.377 - 0.08)		
**parasite stage:predator**	**mature absent - mature present**	**-0.462 (-0.565 - -0.361)**		
parasite stage:predator	mix absent - mix dead end	-0.574 (-1.534 - 0.487)		
**parasite stage:predator**	**mix absent - mix present**	**-0.449 (-0.646 - -0.257)**		
parasite stage:predator	switching absent - switching dead end	-0.352 (-3.636 - 2.9)		
**parasite stage:predator**	**switching absent - switching present**	**-0.25 (-0.443 - -0.062)**		
parasite stage:predator	immature dead end - immature present	0.239 (-0.197 - 0.676)		
**parasite stage:predator**	**mature dead end - mature present**	**-0.317 (-0.564 - -0.084)**		
parasite stage:predator	mix dead end - mix present	0.128 (-0.944 - 1.087)		
parasite stage:predator	switching dead end - switching present	0.102 (-3.228 - 3.304)		

N indicates the number of observations/ Number of independent studies. Unique taxa indicates the number of unique host-parasite-pairs (unique host taxa/unique parasite taxa). As far as possible, these estimates are based on species. However, in a limited number of cases, in which species level identification of parasites was not available, it is based on genus. Bold rows indicate estimates or contrasts whose HPD (High posterior density) range does not overlap with 0, i.e. that estimates for these levels differ from 0 and from each other respectively.

Table S9: Estimated marginal means and contrasts. Models include parasite stage (full model on all parasites only), behavior, and predator type as fixed effects.

A: Full model (all parasites)				
Estimates for fixed factors				
Factor	Level	Emmean/ trend (HPD range)	N	Unique taxa
**overall**	**overall**	**0.49 (0.143 - 0.805)**	**1634/207**	**113(80/82)**
**SE**	**overall**	**1.441 (1.082 - 1.771)**	**1634/207**	**113(80/82)**
parasite stage	immature	0.099 (-0.237 - 0.441)	278/49	30(25/27)
**parasite stage**	**mature**	**0.742 (0.405 - 1.062)**	**1177/196**	**110(79/79)**
**parasite stage**	**mix**	**0.775 (0.427 - 1.108)**	**104/11**	**11(10/10)**
**parasite stage**	**switching**	**0.347 (0.01 - 0.7)**	**75/16**	**10(8/9)**
behavior	activity	0.26 (-0.082 - 0.592)	620/90	61(48/43)
**behavior**	**feeding**	**0.403 (0.056 - 0.741)**	**103/19**	**12(10/10)**
**behavior**	**impairment**	**0.735 (0.374 - 1.073)**	**187/33**	**33(25/28)**
**behavior**	**position**	**0.512 (0.184 - 0.852)**	**527/128**	**85(62/60)**
**behavior**	**predation**	**0.391 (0.033 - 0.733)**	**130/50**	**28(21/22)**
**behavior**	**social**	**0.641 (0.254 - 1.04)**	**67/20**	**19(17/16)**
predator	absent	0.308 (-0.028 - 0.621)	1134/181	110(77/81)
**predator**	**dead end**	**0.491 (0.101 - 0.855)**	**79/20**	**13(10/10)**
**predator**	**present**	**0.67 (0.341 - 1.002)**	**421/78**	**44(36/31)**
Contrasts				
Factor	Contrast	Estimate (HPD range)		
**parasite stage**	**immature - mature**	**-0.642 (-0.738 - -0.549)**		
**parasite stage**	**immature - mix**	**-0.676 (-0.818 - -0.547)**		
**parasite stage**	**immature - switching**	**-0.248 (-0.37 - -0.133)**		
parasite stage	mature - mix	-0.034 (-0.151 - 0.075)		
**parasite stage**	**mature - switching**	**0.394 (0.286 - 0.503)**		
**parasite stage**	**mix - switching**	**0.428 (0.288 - 0.562)**		
**behavior**	**activity - feeding**	**-0.144 (-0.285 - -0.014)**		
**behavior**	**activity - impairment**	**-0.477 (-0.65 - -0.314)**		
**behavior**	**activity - position**	**-0.251 (-0.349 - -0.149)**		
behavior	activity - predation	-0.132 (-0.297 - 0.034)		
**behavior**	**activity - social**	**-0.383 (-0.611 - -0.153)**		
**behavior**	**feeding - impairment**	**-0.332 (-0.51 - -0.142)**		
**behavior**	**feeding - position**	**-0.107 (-0.21 - -0.005)**		
behavior	feeding - predation	0.013 (-0.165 - 0.182)		
behavior	feeding - social	-0.237 (-0.483 - 0.011)		
**behavior**	**impairment - position**	**0.225 (0.064 - 0.379)**		
**behavior**	**impairment - predation**	**0.344 (0.139 - 0.545)**		
behavior	impairment - social	0.095 (-0.15 - 0.37)		

behavior	position - predation	0.12 (-0.032 - 0.278)		
behavior	position - social	-0.13 (-0.348 - 0.111)		
behavior	predation - social	-0.251 (-0.518 - 0.012)		
predator	absent - dead end	-0.182 (-0.383 - 0.015)		
**predator**	**absent - present**	**-0.364 (-0.451 - -0.282)**		
predator	dead end - present	-0.182 (-0.387 - 0.024)		
		**B: Mature parasites**		
		Estimates for fixed factors		
Factor	Level	Emmean/ trend (HPD range)	N	Unique taxa
**overall**	**overall**	**0.791 (0.44 - 1.113)**	**1177/196**	**110(79/79)**
**SE**	**overall**	**1.98 (1.569 - 2.39)**	**1177/196**	**110(79/79)**
**behavior**	**activity**	**0.525 (0.195 - 0.881)**	**366/81**	**57(45/39)**
**behavior**	**feeding**	**0.695 (0.329 - 1.03)**	**63/17**	**11(10/9)**
**behavior**	**impairment**	**1.031 (0.691 - 1.406)**	**152/31**	**33(25/28)**
**behavior**	**position**	**0.832 (0.491 - 1.167)**	**440/123**	**81(59/56)**
**behavior**	**predation**	**0.728 (0.37 - 1.087)**	**105/47**	**26(21/21)**
**behavior**	**social**	**0.935 (0.537 - 1.327)**	**51/19**	**19(17/16)**
**predator**	**absent**	**0.631 (0.291 - 0.949)**	**865/170**	**107(76/78)**
**predator**	**dead end**	**0.767 (0.372 - 1.147)**	**70/18**	**12(9/9)**
**predator**	**present**	**0.976 (0.649 - 1.33)**	**242/73**	**44(36/31)**
		Contrasts		
Factor	Contrast	Estimate (HPD range)		
**behavior**	**activity - feeding**	**-0.17 (-0.306 - -0.029)**		
**behavior**	**activity - impairment**	**-0.507 (-0.694 - -0.335)**		
**behavior**	**activity - position**	**-0.309 (-0.4 - -0.199)**		
**behavior**	**activity - predation**	**-0.201 (-0.381 - -0.016)**		
**behavior**	**activity - social**	**-0.41 (-0.632 - -0.188)**		
**behavior**	**feeding - impairment**	**-0.339 (-0.536 - -0.143)**		
**behavior**	**feeding - position**	**-0.138 (-0.24 - -0.027)**		
behavior	feeding - predation	-0.031 (-0.221 - 0.176)		
behavior	feeding - social	-0.24 (-0.487 - 0.001)		
**behavior**	**impairment - position**	**0.2 (0.034 - 0.38)**		
**behavior**	**impairment - predation**	**0.306 (0.085 - 0.535)**		
behavior	impairment - social	0.097 (-0.167 - 0.354)		
behavior	position - predation	0.106 (-0.074 - 0.279)		
behavior	position - social	-0.103 (-0.338 - 0.111)		
behavior	predation - social	-0.209 (-0.471 - 0.066)		
predator	absent - dead end	-0.134 (-0.363 - 0.096)		
**predator**	**absent - present**	**-0.345 (-0.458 - -0.24)**		
predator	dead end - present	-0.21 (-0.458 - 0.025)		

**C: Immature parasites**				
Estimates for fixed factors				
Factor	Level	Emmean/ trend (HPD range)	N	Unique taxa
overall	overall	-0.394 (-1.034 - 0.229)	278/49	30(25/27)
SE	overall	-0.785 (-1.546 - 0.006)	278/49	30(25/27)
behavior	activity	-0.28 (-0.899 - 0.355)	138/26	18(16/17)
behavior	feeding	-0.261 (-0.976 - 0.417)	30/3	2(2/2)
behavior	impairment	0.119 (-0.607 - 0.824)	28/7	5(5/5)
behavior	position	-0.293 (-0.945 - 0.321)	56/21	17(14/17)
behavior	predation	-0.222 (-0.917 - 0.437)	18/9	9(7/8)
**behavior**	**social**	**-1.418 (-2.562 - -0.242)**	**8/3**	**3(2/3)**
predator	absent	-0.407 (-1.036 - 0.198)	166/38	29(24/27)
predator	dead end	-0.588 (-1.382 - 0.16)	8/1	1(1/1)
predator	present	-0.179 (-0.797 - 0.446)	104/21	11(9/9)
Contrasts				
Factor	Contrast	Estimate (HPD range)		
behavior	activity - feeding	-0.017 (-0.362 - 0.341)		
behavior	activity - impairment	-0.388 (-0.838 - 0.012)		
behavior	activity - position	0.019 (-0.224 - 0.26)		
behavior	activity - predation	-0.056 (-0.369 - 0.276)		
**behavior**	**activity - social**	**1.139 (0.099 - 2.132)**		
behavior	feeding - impairment	-0.374 (-0.907 - 0.143)		
behavior	feeding - position	0.038 (-0.312 - 0.393)		
behavior	feeding - predation	-0.041 (-0.429 - 0.371)		
**behavior**	**feeding - social**	**1.16 (0.103 - 2.205)**		
behavior	impairment - position	0.406 (-0.015 - 0.859)		
behavior	impairment - predation	0.336 (-0.15 - 0.848)		
**behavior**	**impairment - social**	**1.537 (0.487 - 2.65)**		
behavior	position - predation	-0.075 (-0.399 - 0.256)		
**behavior**	**position - social**	**1.124 (0.094 - 2.128)**		
**behavior**	**predation - social**	**1.199 (0.172 - 2.249)**		
predator	absent - dead end	0.182 (-0.298 - 0.654)		
**predator**	**absent - present**	**-0.23 (-0.376 - -0.079)**		
predator	dead end - present	-0.41 (-0.928 - 0.07)		

N indicates the number of observations/ Number of independent studies. Unique taxa indicates the number of unique host-parasite-pairs (unique host taxa/unique parasite taxa). As far as possible, these estimates are based on species. However, in a limited number of cases, in which species level identification of parasites was not available, it is based on genus. Bold rows indicate estimates or contrasts whose HPD (High posterior density) range does not overlap with 0, i.e. that estimates for these levels differ from 0 and from each other respectively.

Table S10: Estimated marginal means and contrasts for type of next host (A), adult parasite size (B), infection site (C) and host habitat (D). Effects were only observed for mature parasites. For information on other factors included in model see Table 1B.

A: Next host				
**Estimates for fixed factors**				
**Factor**	**Level**	**Emmean/ trend (HPD range)**	**N**	**Unique taxa**
**next host type**	**aquatic vertebrate**	**0.776 (0.386 - 1.164)**	**472/81**	**37(24/27)**
**next host type**	**bird**	**0.919 (0.574 - 1.339)**	**438/99**	**54(43/42)**
next host type	terrestrial vertebrate	0.427 (-0.012 - 0.878)	267/27	19(16/11)
**Contrasts**				
**Factor**	**Contrast**	**Estimate (HPD range)**		
next host type	aquatic vertebrate - bird	-0.147 (-0.442 - 0.109)		
next host type	aquatic vertebrate - terrestrial vertebrate	0.348 (-0.04 - 0.718)		
**next host type**	**bird - terrestrial vertebrate**	**0.497 (0.113 - 0.886)**		
**B: Adult parasite size**				
**Estimates for fixed factors**				
**Factor**	**Level**	**Emmean/ trend (HPD range)**	**N**	**Unique taxa**
adult parasite size	overall	-0.016 (-0.067 - 0.03)	1177/196	110(79/79)
**C: Infection site**				
**Estimates for fixed factors**				
**Factor**	**Level**	**Emmean/ trend (HPD range)**	**N**	**Unique taxa**
**infection site**	**body cavity**	**0.805 (0.415 - 1.258)**	**865/148**	**75(50/50)**
**infection site**	**muscles**	**0.711 (0.195 - 1.245)**	**133/12**	**10(9/9)**
**infection site**	**nervous system incl. eyes**	**1.002 (0.42 - 1.602)**	**65/19**	**9(8/8)**
**infection site**	**other**	**0.537 (0.023 - 1.043)**	**114/18**	**16(15/13)**
**Contrasts**				
**Factor**	**Contrast**	**Estimate (HPD range)**		
infection site	body cavity - muscles	0.098 (-0.323 - 0.552)		
infection site	body cavity - nervous system incl. eyes	-0.194 (-0.696 - 0.31)		
infection site	body cavity - other	0.275 (-0.156 - 0.694)		
infection site	muscles - nervous system incl. eyes	-0.292 (-0.899 - 0.288)		
infection site	muscles - other	0.178 (-0.299 - 0.638)		
infection site	nervous system incl. eyes - other	0.471 (-0.102 - 1.039)		
**D: Host habitat**				
**Estimates for fixed factors**				

A: Next host				
**Estimates for fixed factors**				
**Factor**	**Level**	**Emmean/ trend (HPD range)**	**N**	**Unique taxa**
**Factor**	**Level**	**Emmean/ trend (HPD range)**	**N**	**Unique taxa**
**habitat**	**aquatic**	**0.835 (0.486 - 1.183)**	**879/163**	**86(59/63)**
**habitat**	**terrestrial**	**0.59 (0.185 - 1.009)**	**298/33**	**24(20/16)**

Contrasts		
**Factor**	**Contrast**	**Estimate (HPD range)**
habitat	aquatic - terrestrial	0.242 (-0.095 - 0.565)

N indicates the number of observations/ Number of independent studies. Unique taxa indicates the number of unique host-parasite-pairs (unique host taxa/unique parasite taxa). As far as possible, these estimates are based on species. However, in a limited number of cases, in which species level identification of parasites was not available, it is based on genus. Bold rows indicate estimates or contrasts whose HPD (High posterior density) range does not overlap with 0, i.e. that estimates for these levels differ from 0 and from each other respectively.

Table S11: Results of model comparisons using Leave-One-Out Cross-Validation (LOO). Behavior was retained in all models. Results were obtained through the Bayesian modelling framework (BRMS). Factors that clearly improved the model are indicated in bold (i.e. difference in ELPD larger than two times SE), those for which an improvement was observed, but was unclear due to the difference in ELPD being between one and two times SE are highlighted in italics. Comparisons are always to the model indicated in bold. For immature parasites predator was included for the basic model but not for the one without outliers since it did not clearly improve that model. All models included the following random effects: Paper ID, Host and parasite phylogeny and the interaction between host and parasite. Please refer to Table S5 for model diagnostics.

	basic models				outliers excluded			
model	ELPD loo	p loo	ΔELPD	Δse	ELPD loo	p loo	ΔELPD	Δse
			**A: Mature parasites**					
+ behavior:trophic link	-1454	323.4	2.26	9.17	-1117	322.9	-1.77	8.36
+ behavior:next host type	*-1449*	*284.1*	*7.24*	*6.37*	-1113	294.8	2.34	6.03
+ behavior:infection site	-1465	306.0	-8.54	4.76	-1121	306.2	-5.83	4.92
+ behavior:parasite type	-1455	301.4	1.12	7.43	*-1106*	*299.7*	*8.71*	*8.68*
+ behavior:host type	-1452	305.2	4.33	7.32	-1114	309.3	1.48	7.72
+ behavior:habitat	-1454	280.1	2.21	3.67	-1118	293.7	-2.63	3.58
**SE + behavior + predator**	**-1457**	**282**	**21.68**	**8.06**	**-1115**	**288.1**	**28.45**	**9.31**
			**B: Immature parasites**					
+ behavior:trophic link	-276	84.2	4.64	6.36	-235	75.7	1.02	6.03
+ behavior:next host type	*-271*	*79.9*	*9.09*	*4.82*	-232	72.6	3.86	4.24
+ behavior:infection site	*-274*	*80.9*	*6.25*	*4.68*	-235	74.0	1.10	3.33
+ behavior:parasite type	*-272*	*85.1*	*8.15*	*5.88*	-236	78.5	0.06	4.74
+ behavior:host type	*-272*	*81.2*	*8.19*	*5.55*	-233	74	2.73	5.02
**+ behavior:habitat**	**-270**	**77.8**	**10.61**	**4.30**	*-231*	*70.6*	*5.02*	*3.04*
**SE + behaviour + (predator)**	**-280**	**75.9**	**7.39**	**3.59**	**-236**	**68.4**	**1.64**	**4.19**

ELPD loo: Expected log predictive density estimated via LOO cross-validation; p loo: Effective number of parameters, indicating model complexity; ΔELPD: Difference in ELPD loo between models; Δse: Standard error of ΔELPD, indicating uncertainty in model differences.

Table S12: Estimated marginal means and contrasts for the interaction between behaviour and next host. Please note that only comparisons involving the same type of behaviour are included in the table. (see Table S8). Results were obtained through the Bayesian modelling framework (BRMS). Factors that clearly improved the model are indicated in bold (i.e. difference in ELPD larger than two times SE), those for which an improvement was observed, but was unclear due to the difference in ELPD being between one and two times SE are highlighted in italics. Comparisons are always to the preceding model that was clearly better (i.e. highlighted in bold). All models included the following random effects: Paper ID, Host and parasite phylogeny and the interaction between host and parasite.

Estimates for fixed factors			
**Level**	**Emmean/ trend (HPD range)**	**N**	**Unique taxa**
**activity aquatic vertebrate**	**0.464 (0.12 - 0.81)**	**223/34**	**21(15/18)**
**activity bird**	**0.583 (0.251 - 0.955)**	**75/33**	**22(20/15)**
activity terrestrial vertebrate	0.325 (-0.084 - 0.72)	68/17	14(12/7)
feeding aquatic vertebrate	0.234 (-0.308 - 0.776)	31/5	4(3/4)
**feeding bird**	**0.773 (0.438 - 1.128)**	**30/11**	**5(5/4)**
feeding terrestrial vertebrate	0.591 (-0.439 - 1.577)	2/1	2(2/1)
**impairment aquatic vertebrate**	**1.077 (0.695 - 1.477)**	**15/9**	**6(6/6)**
**impairment bird**	**1.29 (0.883 - 1.701)**	**46/16**	**18(13/16)**
**impairment terrestrial vertebrate**	**0.658 (0.198 - 1.117)**	**91/7**	**9(7/7)**
**position aquatic vertebrate**	**0.942 (0.582 - 1.27)**	**163/49**	**29(18/21)**
**position bird**	**0.893 (0.56 - 1.226)**	**194/67**	**38(32/27)**
**position terrestrial vertebrate**	**0.522 (0.128 - 0.927)**	**83/15**	**14(13/8)**
**predation aquatic vertebrate**	**0.883 (0.499 - 1.27)**	**34/19**	**11(8/9)**
**predation bird**	**0.711 (0.334 - 1.084)**	**62/28**	**12(12/10)**
predation terrestrial vertebrate	0.298 (-0.231 - 0.827)	9/4	3(3/3)
**social aquatic vertebrate**	**0.777 (0.334 - 1.239)**	**6/4**	**4(4/3)**
**social bird**	**1.336 (0.942 - 1.726)**	**31/11**	**11(10/10)**
social terrestrial vertebrate	0.423 (-0.046 - 0.932)	14/4	4(4/4)

Contrasts	
**Contrast**	**Estimate (HPD range)**
activity aquatic vertebrate - activity bird	-0.12 (-0.405 - 0.165)
activity aquatic vertebrate - activity terrestrial vertebrate	0.139 (-0.213 - 0.511)
**feeding aquatic vertebrate - feeding bird**	**-0.539 (-1.031 - -0.041)**
feeding aquatic vertebrate - feeding terrestrial vertebrate	-0.351 (-1.391 - 0.667)
impairment aquatic vertebrate - impairment bird	-0.217 (-0.61 - 0.21)
impairment aquatic vertebrate - impairment terrestrial vertebrate	0.419 (-0.031 - 0.89)
position aquatic vertebrate - position bird	0.046 (-0.209 - 0.3)
**position aquatic vertebrate - position terrestrial vertebrate**	**0.419 (0.05 - 0.767)**

predation aquatic vertebrate - predation bird	0.17 (-0.179 - 0.522)
**predation aquatic vertebrate - predation terrestrial vertebrate**	**0.582 (0.07 - 1.133)**
**social aquatic vertebrate - social bird**	**-0.562 (-1.007 - -0.101)**
social aquatic vertebrate - social terrestrial vertebrate	0.351 (-0.177 - 0.887)
activity bird - activity terrestrial vertebrate	0.26 (-0.116 - 0.653)
feeding bird - feeding terrestrial vertebrate	0.183 (-0.849 - 1.159)
**impairment bird - impairment terrestrial vertebrate**	**0.634 (0.142 - 1.107)**
**position bird - position terrestrial vertebrate**	**0.373 (0.013 - 0.738)**
predation bird - predation terrestrial vertebrate	0.412 (-0.104 - 0.97)
**social bird - social terrestrial vertebrate**	**0.912 (0.417 - 1.434)**

N indicates the number of observations/ Number of independent studies. Unique taxa indicates the number of unique host-parasite-pairs (unique host taxa/unique parasite taxa). As far as possible, these estimates are based on species. However, in a limited number of cases, in which species level identification of parasites was not available, it is based on genus. Bold rows indicate estimates or contrasts whose HPD (High posterior density) range does not overlap with 0, i.e. that estimates for these levels differ from 0 and from each other respectively.
